# Enhancing Structural Diversity of Lathyrane Derivatives through Biotransformation by the Marine-Derived Actinomycete *Streptomyces puniceus* BC-5GB.11

**DOI:** 10.3390/ijms25042289

**Published:** 2024-02-14

**Authors:** Felipe Escobar-Montaño, Victoria E. González-Rodríguez, Antonio J. Macías-Sánchez, José M. Botubol-Ares, Rosa Durán-Patrón, Rosario Hernández-Galán

**Affiliations:** 1 Departamento de Química Orgánica, Facultad de Ciencias, Universidad de Cádiz, Puerto Real, 11510 Cádiz, Spain; felipe.escobarmonta@gm.uca.es (F.E.-M.); antoniojose.macias@uca.es (A.J.M.-S.); rosario.hernandez@uca.es (R.H.-G.); 2 Laboratorio de Microbiología, Departamento de Biomedicina, Biotecnología y Salud Pública, Facultad de Ciencias del Mar y Ambientales, Universidad de Cádiz, Puerto Real, 11510 Cádiz, Spain; victoriaeugenia.gonzalez@uca.es; 3 Instituto Universitario de Investigación en Biomoléculas, Universidad de Cádiz, Puerto Real, 11510 Cádiz, Spain; 4 Instituto Universitario de Investigación Vitivinícola y Agroalimentaria, Universidad de Cádiz, Puerto Real, 11510 Cádiz, Spain

**Keywords:** marine-derived actynomicete, *Streptomyces*, diterpenoids, lathyranes, biotransformation

## Abstract

Lathyrane-type diterpenes have a wide range of biological activities. Among them, euphoboetirane A (**1**) exerts neurogenesis-promoting activity. In order to increase the structural diversity of this type of lathyrane and explore its potential use in neurodegenerative disorders, the biotransformation of **1** by *Streptomyces puniceus* BC-5GB.11 has been investigated. The strain BC-5GB.11, isolated from surface sediments collected from the intertidal zone of the inner Bay of Cadiz, was identified as *Streptomyces puniceus*, as determined by phylogenetic analysis using 16S rRNA gene sequence. Biotransformation of **1** by BC-5GB.11 afforded five products (**3**–**7**), all of which were reported here for the first time. The main biotransformation pathways involved regioselective oxidation at non-activated carbons (**3**–**5**) and isomerization of the ∆^12,13^ double bond (**6**). In addition, a cyclopropane-rearranged compound was found (**7**). The structures of all compounds were elucidated on the basis of extensive NMR and HRESIMS spectroscopic studies.

## 1. Introduction

Lathyranes are polyoxygenated macrocyclic diterpenoids with a 5/11/3-fused-ring skeleton that are widely distributed in plants from the genera Euphorbia and Jatropha [1,2,3,4,5]. They stand out for their high structural diversity as well as for the variety of biological activities that they display [6]. A great number of lathyrane diterpenoids have shown the capability to modulate MDR, cytotoxicity against cancer cell lines, anti-inflammatory activity, and the ability to induce cell proliferation or differentiation of neural stem cells (NSC) [6]. Some structural features, such as a fused epoxy ring, the *gem*-dimethylcyclopropane ring, the oxidation degree, and the nature of the ester chains attached to the main skeleton, could be involved in the substrate-target biological interactions.

**Figure 1:**
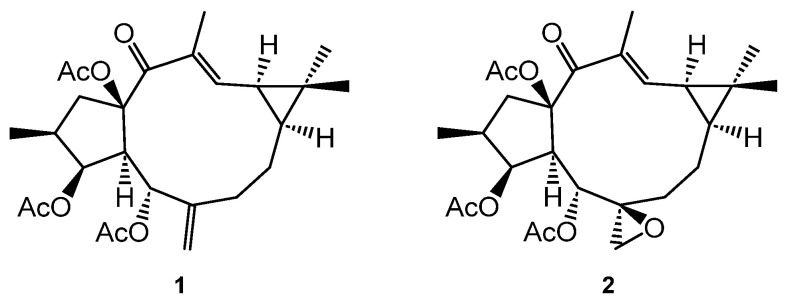
Lathyranes euphoboetirane A (**1**) and epoxyboetirane A (**2**).

In previous studies, we have described that the lathyranes euphoboetirane A (**1**) and epoxyboetirane A (**2**) (Figure 1), isolated from *Euphorbia boetica*, increased the size of neurospheres in a dose-dependent manner in cultures stimulated with a combination of two growth factors, epidermal (EGF) and basic fibroblast (bFGF), in a synergist way. The results suggested that they act on receptors different from epidermal growth factor receptor (EGFR) or fibroblast growth factor receptor (FGFR) [7]. On the other hand, it has been proven that lathyrane compounds can exert significantly different abilities to activate PKCs and promote NPC proliferation or differentiation [8,9] without knowing what the precise structural factors that determine the type of activity presented by these compounds are. Therefore, there is an interest in lathyrane-based library preparation with high structural diversity to explore its potential use in neurodegenerative disorders.

Microbial transformation is a really useful alternative to chemical approaches for performing highly regio- and stereoselective hydroxylations at non-activated carbons and rearrangement reactions [10,11,12,13]. To the best of our knowledge, only two precedents for the biotransformation of lathyrane diterpenoids have been described. Wu et al. reported the microbial transformation of three lathyrane diterpenoids (lathyrol, 7*β*-hydroxylathyrol, and Euphorbia factor L_3_) by *Mortierella ramanniana* CGMCC 3.03413, *Mucor circinelloides* CICC 40242, and *Nocardia iowensis* sp. nov. NRRL 5646. Eight new metabolites were obtained as a result of the regioselective hydroxylation at C-7, C-8, C-18, or C-19 positions, together with two 10,11-secolathyranes [11]. Recently, Euphorbia factor L_1_ and its deacylated derivative were biotransformed in their deoxy derivatives by *Mucor polymorphosporus* and *Cunninghamella elegans* [14]. Moreover, euphorbiasteroid was glycosylated, regioselectively hydroxylated, or methyl oxidized by another *C. elegans* strain, bio-110930 [15].

This paper reports on the isolation and identification of a *Streptomyces puniceus* strain isolated from sediment samples from the intertidal zone of the inner Bay of Cadiz (Cádiz, Spain). In order to broaden the structural diversity of lathyrane diterpenoids and further study the relationship between the functionalization presented by these diterpenes and their neurogenesis-promoting activity, we also described the biotransformation of euphoboetirane A (**1**), a lathyrane-type diterpenoid isolated from *E. boetica* by *Streptomyces puniceus* BC-5GB.11.

## 2. Results and Discussion

**Figure 2:**
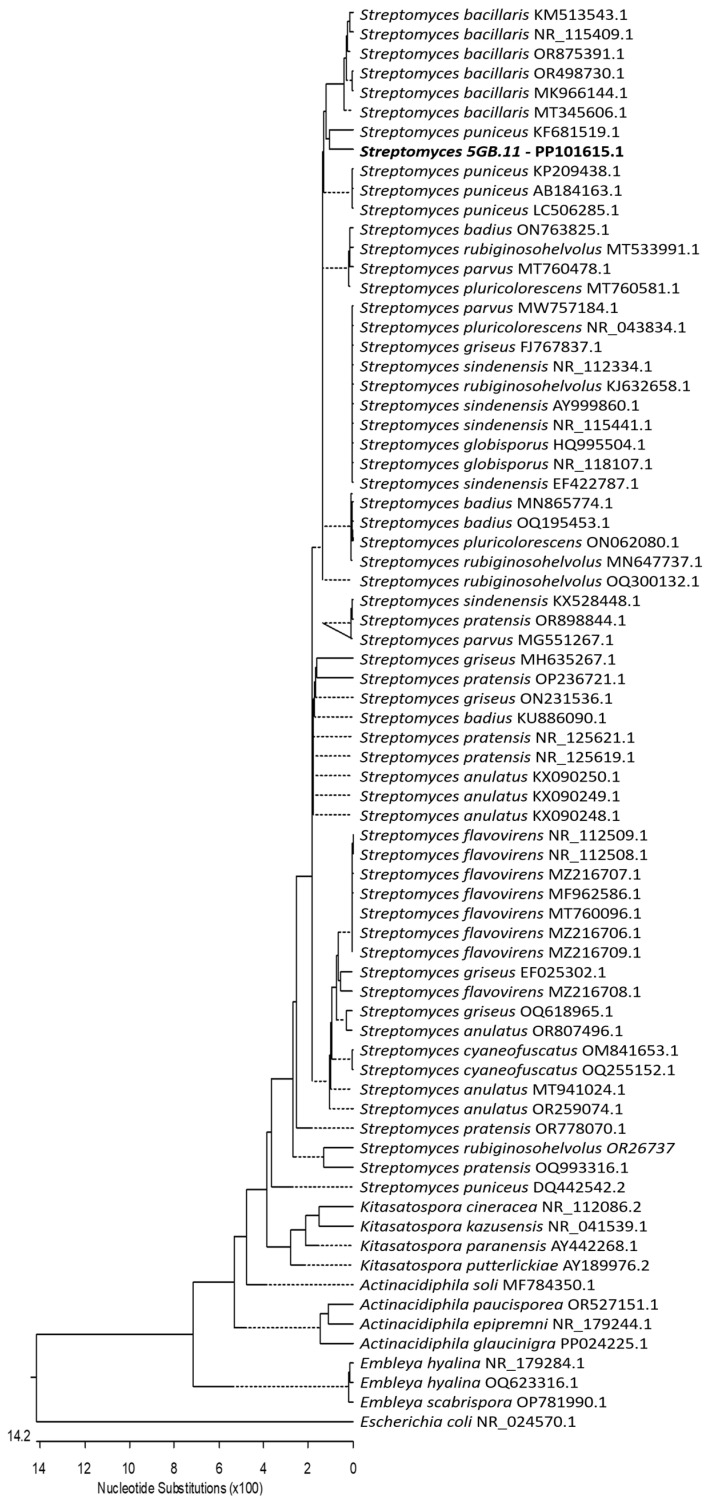
Neighbor-joining tree constructed using 16S-rRNA gene sequences, comprising sequences identified in this study (highlighted in bold) and published sequences obtained from the GenBank database. The length of each branch pair reflects the distance between the respective sequence pairs. A dotted line on the tree denotes a negative branch length, while the bar indicates the number of nucleotide substitutions.

Actinomycete strain BC-5GB.11 was isolated from surface sediments collected in the intertidal zone of the inner Bay of Cadiz (Cádiz, Spain). This strain was sent for sequencing and identification to the identification service of the Spanish Type Culture Collection (CECT, https://www.uv.es/cect, accessed on 28 December 2023). The 16S-rRNA gene was sequenced and compared with those in NCBI databases. As a result, CECT identified strain BC-5GB.11 as *Streptomyces*. Then, a neighbor-joining phylogenetic analysis was conducted using the Kimura two-parameter model and a bootstrap test with 5000 runs (MegAlign, DNASTAR^®^ La-Sergene package). A total of seventy-two sequences of the ribosomal DNA region comprising the 16S rRNA gene were downloaded from the Gen-Bank database. These sequences were selected from related species and genera in the family Streptomycetaceae and included five genera and twenty-four species. Based on all these studies, it was determined that strain BC-5GB.11 is grouped with the species *S. puniceus* (Figure 2).

*E. boetica* was collected at “El Pinar del Hierro” in Chiclana de la Frontera, Cádiz (Spain). The *n*-hexane-soluble fraction of the methanolic extract of the aerial parts of *E. boetica* was fractionated and purified by silica gel column chromatography to afford the lathyrane euphoboetirane A (**1**), as indicated in the Section 3.

**Figure 3:**
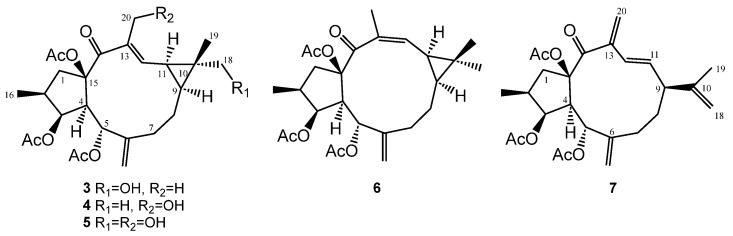
Biotransformation products of euphoboetirane A (**1**) by *Streptomyces puniceus* BC-5GB.11.

Preparative scale microbial transformation of **1** by *S. puniceus* BC-5GB.11 furnished three new hydroxylated lathyranes **3**–**5**, one derivative with an isomerization of the ∆^12,13^ double bond (**6**), and one product where a cleavage for the cyclopropane ring has occurred (**7**) (Figure 3).

The molecular formulas for compounds **3** and **4**, C_26_H_36_O_8_, were identical. They were determined from [M+Na]^+^ molecular ions in their HRESIMS spectra (*m/z* 499.2322 and 499.2298, respectively, calculated for C_26_H_36_O_8_Na 499.2308) (Figures S8 and S16) and were 16 mass units higher than that of **1**, suggesting that both were monohydroxylated derivatives of euphoboetirane A (**1**).

**Figure 4:**
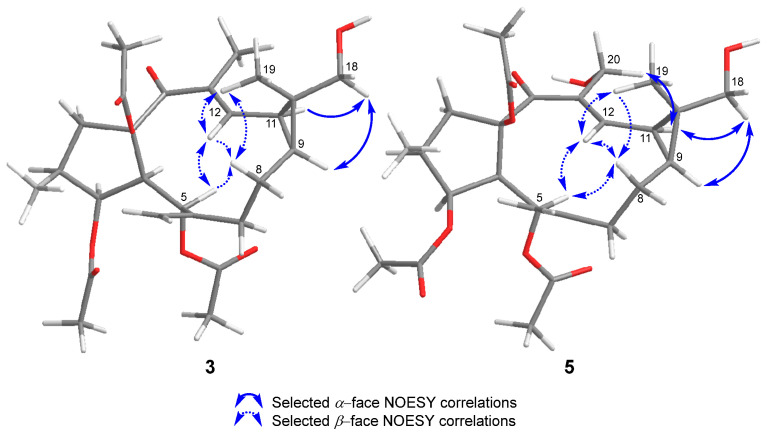
Selected NOESY correlations for compounds **3** and **5**.

Their ^1^H NMR data (Table 1 and Figures S1 and S10) showed a deshielding of the signals corresponding to H-9 and H-11 (from 1.15 and 1.39 ppm for **1** [16] to 1.25 and 1.53 ppm for **3**, and 1.27 and 1.59 ppm for **4**, respectively). In addition, each one showed the absence of one of the singlet methyl signals and the appearance of new signals corresponding to methylenes bound to oxygen (3.46 and 3.39 ppm, d, *J* = 11.1 Hz, and 4.32 and 4.17 ppm, d, *J* = 12.2 Hz for **3** and **4**, respectively). The disappearance of the signal corresponding to the methyl on the double bond (C-20) in compound **4** placed the new hydroxyl group at position C-20. In compound **3,** it must be located on one of the methyls of the *gem*-dimethyl group. The exact location was established through the nOe effects observed between this methylene group and H-9 and H-11 (Figure 4 and Figure S7c).

Based on the NOESY correlations (Figures S6, S7a–d, and S15a–d), the absolute configuration described for euphoboetirane A (**1**) [16,17,18,19], and the similarity of the electronic circular dichroism (ECD) spectra of **3** and **4** (Figures S9 and S17) to that of **1** [16], the structures of **3** and **4** were determined as (10*R*)-18-hydroxyeuphoboetirane A and 20-hydroxyeuphoboetirane A, respectively.

Compound **5** showed spectroscopic characteristics common to both **3** and **4** (Table 1). Its molecular formula, C_26_H_36_O_9_, deduced from a [M+Na]^+^ molecular ion in its HRESIMS (*m/z* 515.2260 [M+Na]^+^, calculated for C_26_H_36_O_8_Na 515.2257) (Figure S25), indicated the presence of 32 additional mass units to that of compound **1**, which was in agreement with the presence of two extra hydroxyl groups in the molecule. They were located on C-18 and C-20, as supported by the HMBC correlations between H_2_-18 and C-9 and between H-12 and C-20 (Figures S22 and S45).

The relative stereochemistry of the C-18-hydroxyl group was deduced as *α* by the NOESY correlations between H_2_-18 and H-9 and H-11 (Figure 4 and Figure S24c). The same configuration as **3** was inferred for **5** based on additional NOESY correlations between H-12 and H-5, H-8*β*, and H_3_-19; H-5 and H-8*β*; H_2_-20 and H-11; and between H_3_-19 and H-8*β* (Figures S23 and S24a,e,d). As a result, the structure of **5** was established as (10*R*)-18,20-dihydroxyeuphoboetirane A.

On the other hand, compound **6** showed a molecular formula, C_26_H_36_O_7_, determined by HRMS (*m/z* 483.2377 [M+Na]^+^, calculated for C_26_H_36_O_7_Na 483,2359) (Figure S34), which was identical to that of compound **1**. The HMBC correlations (Figures S31 and S45) observed indicated the same connectivity as **1**, therefore it might be an isomeric compound. The principal difference observed in its ^1^H NMR spectrum (Figure S27) was the large shielding of proton H-12 (from 6.48 ppm in **1** [16] to 5.28 ppm in **6**, Table 2), as well as its coupling constant (d, *J* = 11.4 Hz in **1** [16] and dd, *J* = 8.7, 1.6 Hz in **6**). Furthermore, the ^13^C NMR spectrum of **6** (Figure S28) showed a shift of the signals corresponding to C-12 and C-20 to upfield and downfield, respectively, in comparison to **1** (*δ*_C_ 146.7 for C-12 and *δ*_C_ 12.5 for C-20 in **1** [16]; *δ*_C_ 134.8 for C-12 and *δ*_C_ 22.2 for C-20 in **6**). These data indicate that **6** might be the *Z* isomer of **1** [20].

**Figure 5:**
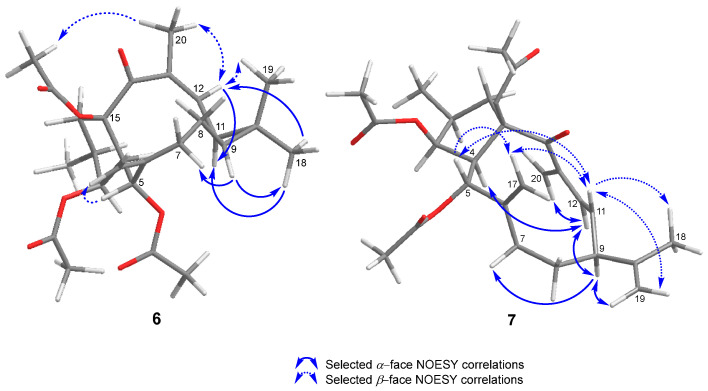
Selected NOESY correlations for compounds **6** and **7**.

The isomerization was supported by the NOESY correlation observed between H-12 and H_3_-20 (Figure 5, Figures S32 and S33b,c) and by the negative cotton effect (∆*ε* −4.06) observed in its ECD spectrum (Figure S35) at 228 nm, opposite to that shown by **1** [16]. Accordingly, the structure of **6** was assigned as (12*Z*)-euphoboetirane A.

Finally, compound **7** showed a molecular ion at *m/z* 481.2193 [M+Na]^+^ (calc. for C_26_H_34_O_7_Na 481.2202) (Figure S43), which allowed us to deduce its molecular formula as C_26_H_34_O_7_. The ^1^H NMR spectrum (Figure S36) of compound **7** showed similar main features for ring A to those of the previous compounds, but a significant change in rings B-C could be inferred from it. The characteristic signals for H-9 and H-11, as well as the signals corresponding to the *gem*-dimethylcyclopropane fragment, were missing (Table 2). Conversely, a spin system H_2_-7/H_2_-8/H-9/H-11/H-12 was identified in its ^1^H-^1^H COSY spectrum (Figure S38), which pointed to a C-10/C-11 bond cleavage of the cyclopropane ring. Indeed, proton and carbon resonances characteristic of an isopropenyl system were present. Moreover, olefinic protons located at C-10 (19), C-11 (12), and C-13 (20) were also observed in their NMR spectra (Figures S36 and S37).

HMBC correlations from H-11 to C-8, C-9, and C-13 and from H-12 to C-9, C-13, C-14, and C-20 (Figures S40 and S45) confirmed the cyclopropane rearrangement to a secolathyrane skeleton. Additional HMBC correlations from H-9 to C-8, C-10, C-11, C-12, C-18, and C-19, and from H_2_-18 to C-9, C-10, and C-19, indicated the presence of a conjugated double bond system between C-11/C-12 and C-13/C-20.

The configuration of the ∆^11,12^ doubled bond was assigned as *E* because of the large coupling constant between H-11 and H-12 (*J* = 15.8 Hz), as well as the NOESY correlations observed between H-11 and H-5, H_2_-17, H_3_-18, and H_2_-19; and between H-12 and H-4, H-9, and H_2_-20 (Figure 5, Figures S41 and S42a,c). On the other hand, the stereochemistry of the isopropenyl group at C-9 was assigned as *β* based on the NOESY correlations observed between H-9, H-7α, and H-12 (Figures S42a and S42h). As a result, the structure of **7** was established as (2*S*,3*S*,4*R*,5*S*,9*S*,15*R*)-3,5,15-tri-*O*-acetyl-10,11-secolathyra-6(17),10(19),11*E*,13(20)-tetraen-14-one. This cyclopropane rearrangement has been previously reported for the biotransformation of lathyrol and 7*β*-lathyrol by *M. ramanniana* [11].

## 3. Materials and Methods

### 3.1. General Experimental Procedures

Optical rotations were determined with a JASCO P-2000 polarimeter (JASCO, Tokyo, Japan). Infrared spectra were recorded on a PerkinElmer Spectrum BX FT-IR spectrophotometer (PerkinElmer, Waltham, CA, USA) and reported as wave number (cm^−1^). ECD spectra were recorded on a JASCO J-1500 CD spectrometer (JASCO, Tokyo, Japan). ^1^H and ^13^C NMR measurements were recorded on an Agilent 400 MHz NMR spectrometer (Agilent, Santa Clara, CA, USA) with SiMe_4_ as the internal reference. Chemical shifts are expressed in ppm (*δ*), referenced to CDCl_3_ (Eurisotop, Saint-Aubiu, France, *δ*_H_ 7.25, *δ*_C_ 77.0). COSY, HSQC, HMBC, and NOESY experiments were performed using a standard Agilent pulse sequence. Spectra were assigned using a combination of 1D and 2D techniques. HRMS was performed in a Q-TOF mass spectrometer (Xevo-G2-S QTOF; Waters, Manchester, UK) in the positive-ion ESI mode. TLC was performed on Merck Kiesegel 60 Å F_254_, 0.25 mm layer thickness. Silica gel 60 (60−200 µm, VWR) was used for column chromatography. Purification using HPLC was performed with a Merck-Hitachi Primade apparatus equipped with a UV−vis detector (Primaide 1410) and a refractive index detector (RI-5450), and a Merck-Hitachi LaChrom apparatus equipped with a UV−vis detector (L 4250) and a differential refractometer detector (RI-7490) (Merck, Darmstadt, Germany). LiChroCART LiChrospher Si 60 (5 µm, 250 mm × 4 mm), LiChroCART LiChrospher Si 60 (10 µm, 250 mm × 10 mm), and ACE 5 SIL (5 µm, 250 mm × 4.6 mm id) columns were used for isolation experiments.

### 3.2. Plant Material

The whole plants of *Euphorbia boetica* were collected at El Pinar del Hierro (Chiclana de la Frontera, Cádiz, Spain) in March 2020 with the permission of the national competent authorities (Dirección General de Biodiversidad, Bosques y Desertificación, Secretaría de Estado de Medio Ambiente, Ministerio para la Transición Ecológica y Reto Demográfico, reference number ESNC64, and Consejería de Agricultura, Ganadería Pesca y Desarrollo Sostenible-Delegación Territorial de Cádiz, Junta de Andalucía, reference number 201999901092011).

### 3.3. Microorganisms and Their Identification

The actinomycete *Streptomyces puniceus* BC-5GB.11 was isolated from intertidal sediments collected in the inner Bay of Cadiz (Cádiz, Spain) within a *Spartina* spp. bed with the permission of the national competent authority (ABSCH-CNA-ES-240784-3, reference number ESNC84). Surface sediment samples were collected aseptically in the field, stored in sterile packaging, kept on ice, brought to the laboratory, and immediately processed. Sediment was diluted with sterile seawater (SSW), and aliquots were grown on potato dextrose agar (PDA) plates and marine agar plates (Condalab S.L.) and incubated at 25 °C for 5–10 d. Bacterial colonies were selected and streaked on PDA plates under axenic conditions. The isolates were maintained on PDA at 25 °C for routine experiments, and spores were stored in 60% (*v*/*v*) glycerol at −20 °C for later studies.

The BC-5GB.11 bacterial strain isolated was identified using the service of the Spanish Type Culture Collection (CECT, https://www.uv.es/cect, accessed on 28 December 2023) based on molecular techniques. Amplification and sequencing (with readings in both directions) of the 16S-rRNA gene were carried out. The sequencing of this region was compared with that in NCBI databases. The sequence was submitted to the NCBI database with the accession number PP101615.1. To study the phylogenetic relationship of our isolate, other sequences of related genera and species (72 sequences) from the family Streptomycetaceae were downloaded from the GenBank database and included in the phylogenetic trees (Figure 2).

This microorganism was preserved on PDA plugs of 1 cm diameter in H_2_O at 4 °C, and it is deposited in the Bacteriological Collection of the University of Cadiz.

### 3.4. Extraction and Isolation of Euphoboetirane A (***1***)

Compound **1** was isolated from the aerial parts of *E. boetica* following the procedure previously described in the literature [7]. The aerial parts of the fresh plant (3.0 kg) were frozen with liquid nitrogen, powdered, and extracted with MeOH (2.5 L × 3) at room temperature for 24 h. The MeOH extract was evaporated under reduced pressure to yield a crude extract, which was suspended in water (1 L) and then extracted with *n*-hexane (1.5 L × 3) and CH_2_Cl_2_ (1.5 L × 3), sequentially. Evaporation of the solvent at reduced pressure yielded 33.3 g and 8.9 g of the *n*-hexane- and CH_2_Cl_2_-soluble extracts, respectively. The *n*-hexane extract was fractionated through silica gel column chromatography, using an increasing gradient of EtOAc in *n*-hexane (10–100%) to afford 21 fractions, according to TLC analysis. Fractions 4–7 were combined and further purified by column chromatography using a gradient mixture of CH_2_Cl_2_ and acetone of increasing polarity (0–2%) to yield **1** (2.0 g).

### 3.5. Biotransformation of Euphoboetirane A (***1***)

*Streptomyces puniceus* BC-5GB.11 was grown in seventeen 500-mililiter Erlenmeyer flasks containing 200 mL of ISP2 medium (4 g of yeast extract, 10 g of malt extract, and 4 g of dextrose per liter of sea water) at pH 7.3. Each flask was inoculated with 1300 µL of a fresh conidial suspension obtained by adding 25 mL of sterile ISP2 medium to seven Petri dishes (9 cm in diameter) cultured with the strain BC-5GB.11 for 17 d. The flasks were shaken at 200 rpm and 25 °C under continuous white light (i.e., daylight lamp) for 3 d. A solution of euphoboetirane A (**1**) in DMSO (300 μL) was then added to each flask to a final concentration of 88 ppm. The culture control consisted of a fermentation flask to which 300 µL of DMSO was added. The substrate control contained sterile ISP2 medium and the same concentration of **1** dissolved in 300 µL of DMSO. All flasks were incubated under identical conditions as described above for an additional period of 11 d. The optimal biocatalysis time was established as a result of a preliminary study to determine the time at which all euphoboetirane A (**1**) is consumed in order to achieve maximum production of the biotransformation products.

### 3.6. Extraction, Isolation, and Characterization of Biotransformation Products

Once the biotransformation process was finished, the broth was vacuum filtered using a 200 µm pore-size Nytal filter. The mycelium was discarded, and the culture medium was saturated with NaCl and extracted with EtOAc (×3). The organic phase was washed with H_2_O (×3), dried over anhydrous Na_2_SO_4,_ and filtered. Evaporation of the solvent under reduced pressure at a rotary evaporator yielded 522.5 mg of crude extract. This extract was purified by column chromatography using a gradient of increasing polarity of *n*-hexane/ethyl acetate. The fractions obtained were analyzed by CCF, combining those with the highest affinity. Successive purifications by semipreparative and analytical HPLC led to the obtaining of the following compounds:

*(10*R*)-18-Hydroxyeuphoboetirane A (**3**)*: Purified through semipreparative HPLC (*n*-hexane:EtOAc 70:30, flow 3.0 mL/min, t_R_ = 57 min), 87.2 mg, 28.1% yield. Amorphous solid; ${\left[\alpha \right]}_{D\;}^{20}$ = +160.0 (c 1.2, MeOH); ECD (MeOH) *λ* (∆ε) 312 (+0.12) nm (Figure S9); UV (MeOH) *λ*_max_ (log ɛ) 313 (3.1970) nm; IR (KBr) *ν*_max_ 2932, 1739, 1372, 1258, 1022 cm^−1^; ^1^H and ^13^C NMR data see Table 1 and Figures S1–S5; HRMS (ESI+) 499.2322 [M+Na]^+^ (calc. for C_26_H_36_O_8_Na 499.2308) (Figure S8).

*20-Hydroxyeuphoboetirane* A *(**4**)*: Purified through semipreparative HPLC (*n*-hexane:EtOAc 65:35, flow 3.0 mL/min, t_R_ = 46 min), 8.3 mg, 2.7% yield. Amorphous solid; ${\left[\alpha \right]}_{D\;}^{20}$ = +154.0 (c 0.6, MeOH); ECD (MeOH) *λ* (∆ε) 318 (+1.06) nm (Figure S17); UV (MeOH) *λ*_max_ (log *ɛ*) 309 (3.180) nm; IR (KBr) *ν*_max_ 2933, 1740, 1372, 1257, 1019 cm^−1^; ^1^H and ^13^C NMR data see Table 1, Figures S10–S14; HRMS (ESI^+^) 499.2298 [M+Na]^+^ (calc. for C_26_H_36_O_8_Na 499.2308).

*(10*R*)-18,20-Dihydroxyeuphoboetirane A (**5**)*: Purified through analytical HPLC (CHCl_3_:MeOH 97:3, flow 1.00 mL/min, t_R_ = 9 min), 4.5 mg, 1.4% yield. Amorphous solid; ${\left[\alpha \right]}_{D\;}^{20}$ = +41.0 (c 0.4, MeOH); ECD (MeOH) *λ* (∆ε) 225 (-2.88), 249 (+2.25), 295 (+2.14) nm (Figure S26); UV (MeOH) *λ*_max_ (log *ɛ*) 300 (2.137) nm; IR (KBr) *ν*_max_ 2931, 1739, 1372, 1260, 1020 cm^−1^; ^1^H and ^13^C NMR data see Table 1, Figures S18–S22; HRMS (ESI^+^) 515.2260 [M+Na]^+^ (calc. for C_26_H_36_O_9_Na 515.2257).

*(12*Z*)-Euphoboetirane A (**6**)*: Purified through analytical HPLC (*n*-hexane:EtOAc 90:10, flow 1.0 mL/min, t_R_ = 84 min), 8.3 mg, 2.7% yield. Amorphous solid; ${\left[\alpha \right]}_{D\;}^{20}$ = −41 (c 0.2, MeOH); ECD (MeOH) *λ* (∆ε) 227 (−4.06) nm (Figure S35); UV (MeOH) *λ*_max_ (log *ɛ*) 298 (1.850) nm; IR (KBr) *ν*_max_ 2932, 1740, 1373, 1250, 1022 cm^−1^; ^1^H and ^13^C NMR data see Table 2, Figures S27–S31; HRMS (ESI^+^) 483.2377 [M+Na]^+^ (calc. for C_26_H_36_O_7_Na 483.2359).

*(2*S*,3*S*,4*R*,5*S*,9*S*,15*R*)-3,5,15-Tri-*O*-acetyl-10,11-secolathyra-6(17),10(19),11*E*,13(20)-tetraen-14-one (**7**)*: Purified through semipreparative HPLC (*n*-hexane:EtOAc 7:3, flow 3.0 mL/min, t_R_ = 13 min), 3.2 mg, 1.0%, yield. Amorphous solid; ${\left[\alpha \right]}_{D\;}^{20}$ = +76.0 (c 0.07, MeOH); ECD (MeOH) *λ* (∆ε) 242 (+10.70) nm (Figure S44); UV (MeOH) *λ*_max_ (log *ɛ*) 295 (1.5246) nm; IR (KBr) *ν*_max_ 2933, 1741, 1372, 1252, 1022 cm^−1^; ^1^H and ^13^C NMR data see Table 2, Figures S36–S40; HRMS (ESI^+^) 481.2193 [M+Na]^+^ (calc. for C_26_H_34_O_7_Na 481.2202).

## 4. Conclusions

Five new lathyrane-type diterpenoids were obtained by microbial transformation of euphoboetirane A (**1**) with *Streptomyces puniceus* BC-5GB.11. This strain, isolated from sediment samples from the intertidal zone of the inner Bay of Cadiz, catalyzed the regioselective hydroxylation of **1** at C-18 and C-20, as well as the isomerization of the ∆^12,13^ double bond. In addition, a rearranged cyclopropane compound was produced (**7**).

These derivatives have contributed to increasing the structural diversity of our lathyrane-based library, which will allow us to further investigate the relationship between the functionalization presented by these diterpenes and their neurogenesis-promoting activity. Studies are in progress to evaluate the ability of major metabolites to promote the release of transforming growth factor *α* (TFG*α*) and neuregulin 1 (NRG1) in cell cultures isolated from the subventricular zone (SVZ) of adult mice, which is an indirect measure to predict the role of these compounds as neurorregenerative agents.

## Figures and Tables

**Table 1:** ^1^H (400 MHz) and ^13^C (100 MHz) NMR spectroscopic data for compounds **3**–**5** in CDCl_3_.

	3		4		5	
Position	*δ*_H_, Mult (*J* in Hz)	*δ*_C_, Type	*δ*_H_, Mult (*J* in Hz)	*δ*_C_, Type	*δ*_H_, Mult (*J* in Hz)	*δ*_C_, Type
1α	3.40, dd (14.6, 8.4)	48.3, CH_2_	3.41, dd (14.5, 8.7)	48.0, CH_2_	3.40, dd (14.5, 8.7)	48.0, CH_2_
1β	1.56, dd (14.6, 11.4)		1.63–1.56, m		1.60, dd (14.5, 11.5)	
2	2.30–2.19, m	37.1, CH	2.33–2.24, m	37.2, CH	2.30–2.20, m	37.2, CH
3	5.53, t (3.5)	80.2, CH	5.57, t (3.5)	80.1, CH	5.57, t (3.5)	80.1, CH
4	2.74, dd (10.4, 3.5)	52.2, CH	2.83, dd (10.3, 3.5)	52.2, CH	2.83, dd (10.3, 3.5)	52.3, CH
5	6.05, d (10.4)	65.7, CH	6.06, d (10.3)	65.6, CH	6.07, d (10.3)	65.5, CH
6	-	144.2, C	-	143.2, C	-	143.2, C
7α	2.12–2.04, m	34.9, CH_2_	2.15–2.09, m	34.7, CH_2_	2.16–2.10, m	34.7, CH_2_
7β	2.30–2.19, m		2.24–2.17, m		2.30–2.20, m	
8α	1.95–1.87, m	21.2, CH_2_	2.05–1.99, m	21.6, CH_2_	2.06–1.99, m	21.2, CH_2_
8β	1.75, m		1.72, tdd (14.0, 11.9, 2.0)		1.84–1.71, m	
9	1.25, ddd (12.2, 8.6, 3.9)	30.8 CH	1.27, ddd (14.0, 8.8, 4.0)	36.4, CH	1.39, ddd (12.1, 8.4, 3.9)	31.8 CH
10	-	30.8, C	-	26.8, C	-	32.0, C
11	1.53, dd (11.3, 8.6)	24.7 CH	1.59, dd (11.6, 8.2)	28.3, CH	1.77, dd (11.6, 8.4)	24.7 CH
12	6.46, dd (11.3, 1.3)	144.9, CH	6.52, d (11.6)	150.8, CH	6.53, d (11.6)	149.0, CH
13	-	135.0, C	-	135.5, C	-	136.4, C
14	-	196.9 C	-	198.7, C	-	198.7 C
15	-	92.3, C	-	92.1, C	-	92.0, C
16	0.88, d (6.7)	14.1, CH_3_	0.91, d (6.7)	14.1, CH_3_	0.91, d (6.7)	14.1, CH_3_
17a	4.98, s	115.6, CH_2_	5.07, d (1.2)	117.6, CH_2_	5.09, s	117.7, CH_2_
17b	4.71, s		4.84, s		4.85, s	
18a	3.46, d (11.1)	71.7, CH_2_	1.16, s	16.8, CH_3_	3.51, d (11.1)	71.7, CH_2_
18b	3.39, d (11.1)				3.40, d (11.1)	
19	1.20, s	12.5, ^a^ CH_3_	1.14, s	28.9, CH_3_	1.22, s	12.5, CH_3_
20a	1.67, d (1.2)	12.4, ^a^ CH_3_	4.32, d (12.2)	58.3, CH_2_	4.31, d (12.4)	58.2, CH_2_
20b			4.17, dd (12.2, 5.9)		4.20, d (12.4)	
20-OH	-	-	2.42, brs	-	-	-
3-OCOCH_3_	2.02, s	20.9, CH_3_	2.03, s	20.9, CH_3_	2.04, ^b^ s	20.9, ^c^ CH_3_
3-OCOCH_3_	-	170.7, C	-	170.7, C	-	170.6, C
5-OCOCH_3_	1.96, s	21.2, CH_3_	1.98, s	21.2, CH_3_	1.99, ^b^ s	21.2, ^c^ CH_3_
5-OCOCH_3_	-	170.6, C	-	170.6, C	-	170.6, C
15-OCOCH_3_	2.08, s	22.0, CH_3_	2.08, s	21.9, CH_3_	2.10, s	22.0, CH_3_
15-OCOCH_3_	-	169.8, C	-	169.8, C	-	169.8, C

^a–c^ Interchangeable signals.

**Table 2:** ^1^H (400 MHz) and ^13^C (100 MHz) NMR spectroscopic data for compounds **6** and **7** in CDCl_3_.

	6		7	
Position	*δ*_H_, Mult (*J* in Hz)	*δ*_C_, Type	*δ*_H_, Mult (*J* in Hz)	*δ*_C_, Type
1α	3.08, dd (13.6, 6.7)	45.6, CH_2_	2.96, dd (14.5, 6.4)	45.9, CH_2_
1β	1.76, t (13.6)		2.24–2.17, m	
2	2.18–2.10, m	37.5, CH	2.18–2.07, m	38.2, CH
3	5.51, t (4.0)	78.5, CH	5.46, t (3.7)	77.2, CH
4	2.77, dd (9.0, 4.0)	51.6, CH	2.87, dd (10.2, 3.7)	51.2, CH
5	5.83, d (9,0)	73.0, CH	5.77, d (10.2)	73.2, CH
6	-	145.1, C	-	142.8, C
7α	1.97–1.84, m	29.7, CH_2_	1.76–1.67, m	26.5, CH_2_
7β	2.29, m		2.09–1.99, m	
8α	1.97–1.84, m	23.0, CH_2_	1.91–1.76, m	26.2, CH_2_
8β	1.11, m		1.91–1.76, m	
9	0.62, ddd (11.0, 8.7, 1.9)	34.8, CH	2.63, td (10.5, 5.7)	52.4, CH
10	-	20.8, C	-	146.5, C
11	1.50, t (8.7)	23.9, CH	5.59, dd (15.8, 10.5)	138.5, CH
12	5.28, dd (8.7, 1.6)	134.8, CH	5.94, d (15.8)	124.5, CH
13	-	138.0, C	-	144.3, C
14	-	204.4, C	-	198.0, C
15	-	92.0, C	-	91.4, C
16	0.91, d (6.6)	13.3, CH_3_	0.91, d (6.4)	13.4, CH_3_
17a	5.33, s	115.5, CH_2_	5.31, d (2.7)	114.5, CH_2_
17b	4.92, s		4.99, bs	
18	1.03, s	15.5, CH_3_	1.71, s	21.1, CH_3_
19	1.04, s	28.2, CH_3_	4.72, d (5.9)	110.2, CH_2_
20a	1.83, s	22.2, CH_3_	5.29, s	115.4, CH_2_
20b			5.25, s	
3-OCOCH_3_	2.04, s	20.9, CH_3_	2.02, s	20.7, CH_3_
3-OCOCH_3_	-	170.4, C	-	170.4, C
5-OCOCH_3_	1.98, s	21.1, CH_3_	1.96, s	20.9, CH_3_
5-OCOCH_3_	-	169.5, C	-	169.5, C
15-OCOCH_3_	2.14, s	21.9, CH_3_	2.23, s	21.7, CH_3_
15-OCOCH_3_	-	170.3, C	-	169.1, C

## Data Availability

The data presented in this study are available in Supplementary Material.

## Supplementary Materials

The following supporting information can be downloaded at: https://www.mdpi.com/article/10.3390/ijms25042289/s1.
