# Supplementary material for: Enhancing Structural Diversity of Lathyrane Derivatives through Biotransformation by the Marine-Derived Actinomycete Streptomyces puniceus BC-5GB.11
Source: Int J Mol Sci. 2024 Feb 14;25(4):2289. doi: 10.3390/ijms25042289 (PMC10889386; doi:10.3390/ijms25042289)
Supplement: Supplementary file 1 [file ijms-25-02289-s001.zip › ijms-2866010-supplementary.pdf]

Enhancing structural diversity of lathyrane derivatives through biotransformation by the marine-derived actinomycete *Streptomyces puniceus* BC-5GB.11

Felipe Escobar-Montaño [1], Victoria E. González-Rodríguez [2], Antonio J. Macías-Sánchez [1,3], José M. Botubol-Ares [1,4,*], Rosa Durán-Patrón [1,3,*], Rosario Hernández-Galán [1,3]

[1] Departamento de Química Orgánica, Facultad de Ciencias, Universidad de Cádiz, Puerto Real, 11510 Cádiz, Spain.

[2] Departamento de Biomedicina, Biotecnología y Salud Pública, Laboratorio de Microbiología, Facultad de Ciencias del Mar y Ambientales, Universidad de Cádiz, Puerto Real, 11510 Cádiz, Spain.

[3] Instituto Universitario de Investigación en Biomoléculas, Universidad de Cádiz, Puerto Real, 11510 Cádiz, Spain.

[4] Instituto Universitario de Investigación Vitivinícola y Agroalimentaria, Universidad de Cádiz, Puerto Real, 11510 Cádiz, Spain.

* Correspondence: josemanuel.botubol@uca.es (J.M.B.-A.); rosa.duran@uca.es (R.D.-P.)

Table of Contents

Figure S1. ^{1}H NMR spectrum (400 MHz) of compound **3** in $CDCl_3$ S4

Figure S2. ^{13}C NMR spectrum (100 MHz) of compound **3** in $CDCl_3$ S5

Figure S3. gCOSY spectrum of compound **3** S6

Figure S4. gHSQC spectrum of compound **3** S7

Figure S5. gHMBC spectrum of compound **3** S8

Figure S6. 2D NOESY spectrum of compound **3** S9

S4

Figures S7a-d. 1D NOESY spectra of compound **3** S10-13

Figure S8. HRMS of compound **3** S14

Figure S9. ECD spectrum of compound **3** S15

Figure S10. ^{1}H NMR spectrum (400 MHz) of compound **4** in $CDCl_3$ S16

Figure S11. ^{13}C NMR spectrum (100 MHz) of compound **4** in $CDCl_3$ S17

Figure S12. gCOSY spectrum of compound **4** S18

Figure S13. gHSQC spectrum of compound **4** S19

Figure S14. gHMBC spectrum of compound **4** S20

Figures S15a-d. 1D NOESY spectra of compound **4** S21-24

Figure S16. HRMS of compound **4** S25

Figure S17. ECD spectrum of compound **4** S26

Figure S18. ^{1}H NMR spectrum (400 MHz) of compound **5** in $CDCl_3$ S27

Figure S19 ^{13}C NMR spectrum (100 MHz) of compound **5** in $CDCl_3$ S28

Figure S20. gCOSY spectrum of compound **5** S29

Figure S21. gHSQC spectrum of compound **5** S30

Figure S22. gHMBC spectrum of compound **5** S31

Figure S23. 2D NOESY spectrum of compound **5** S32

Figures S24a-e. 1D NOESY spectra of compound **5** S33-37

Figure S25. HRMS of compound **5** S38

Figure S26. ECD spectrum of compound **5** S39

Figure S27. ^{1}H NMR spectrum (400 MHz) of compound **6** in $CDCl_3$ S40

Figure S28. ^{13}C NMR spectrum (100 MHz) of compound **6** in $CDCl_3$ S41

Figure S29. gCOSY spectrum of compound **6** S42

Figure S30. gHSQC spectrum of compound **6** S43

Figure S31. gHMBC spectrum of compound **6** S44
Figure S32. 2D NOESY spectrum of compound **6** S45
Figures S33a-h. 1D NOESY spectra of compound **6** S46-53
Figure S34. HRMS of compound **6** S54
Figure S35. ECD spectrum of compound **6** S55
Figure S36. ^{1}H NMR spectrum (400 MHz) of compound **7** in $CDCl_3$ S56
Figure S37. ^{13}C NMR spectrum (100 MHz) of compound **7** in $CDCl_3$ S57
Figure S38. gCOSY spectrum of compound **7** S58
Figure S39. gHSQC spectrum of compound **7** S59
Figure S40. gHMBC spectrum of compound **7** S60
Figure S41. 2D NOESY spectrum of compound **7** S61
Figures S42a-h. 1D NOESY spectra of compound **7** S62-69
Figure S43. HRMS of compound **7** S70
Figure S44. ECD spectrum of compound **7** S71
Figure S45. Selected HMBC correlations (arrow) for compounds **3-7** S72

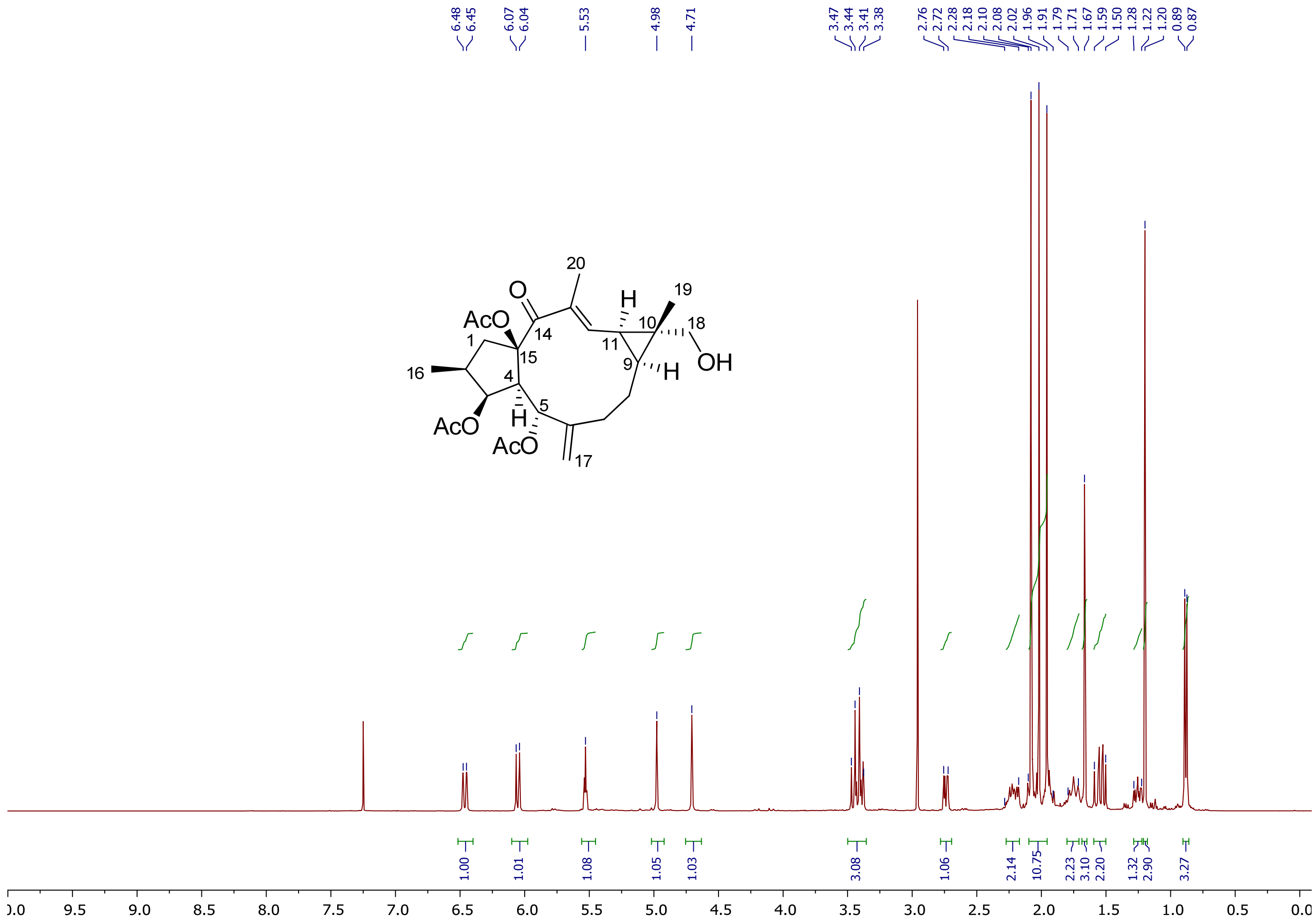

Figure S1. ^{1}H NMR spectrum (400 MHz) of compound **3** in $CDCl_3$.

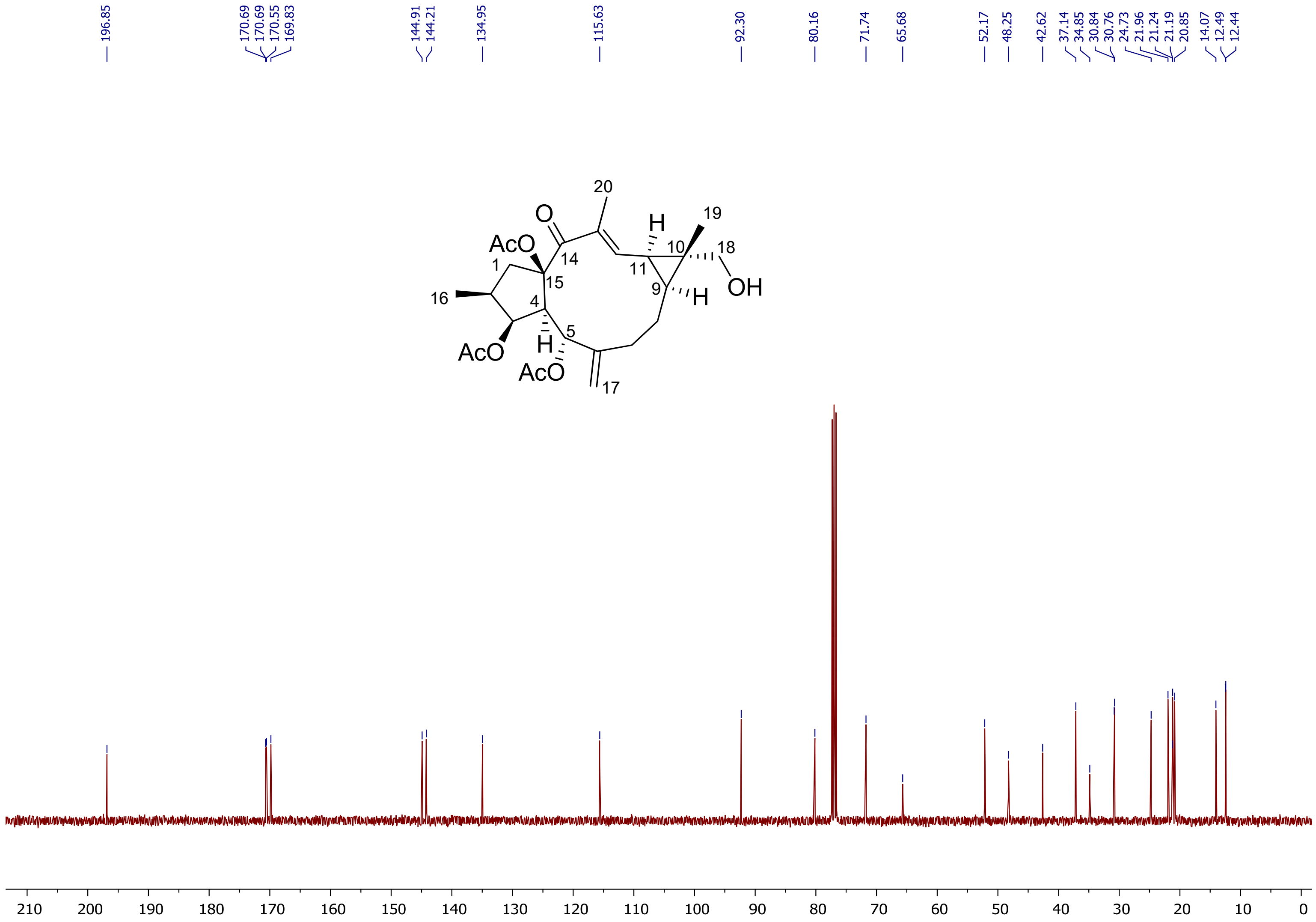

Figure S2. ^{13}C NMR spectrum (100 MHz) of compound **3** in $CDCl_3$.

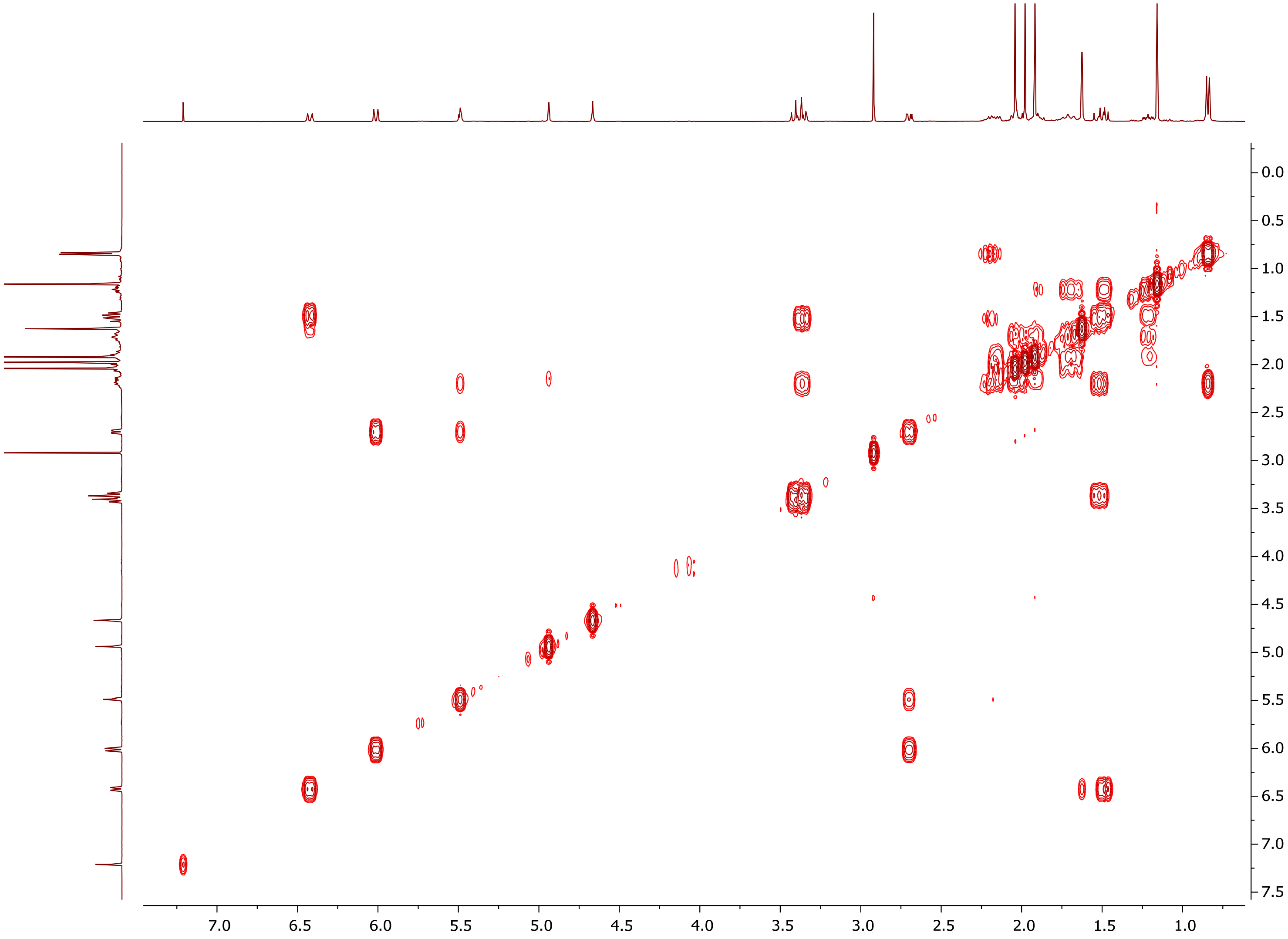

Figure S3. gCOSY spectrum of compound **3**.

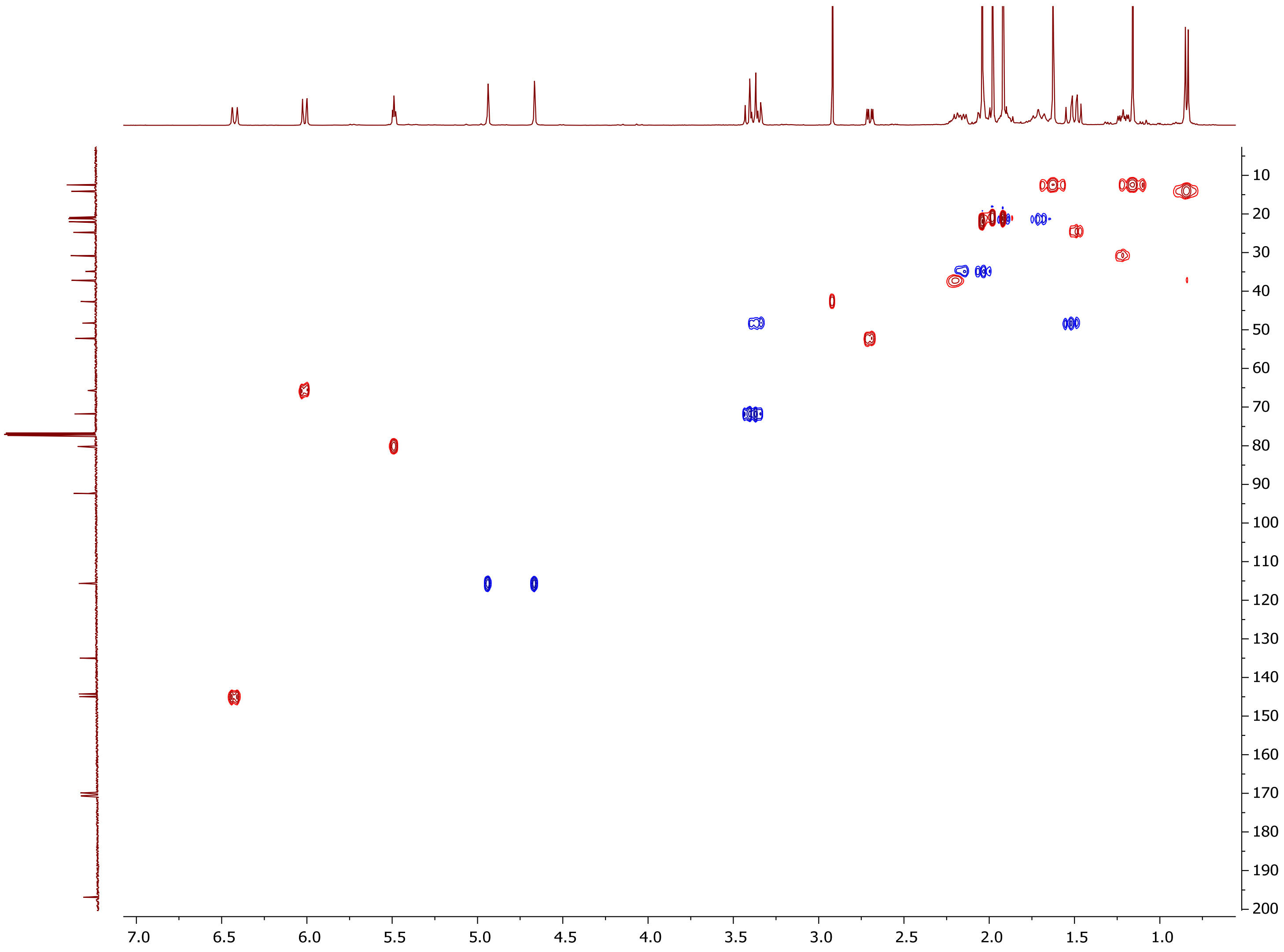

Figure S4. gHSQC spectrum of compound **3**.

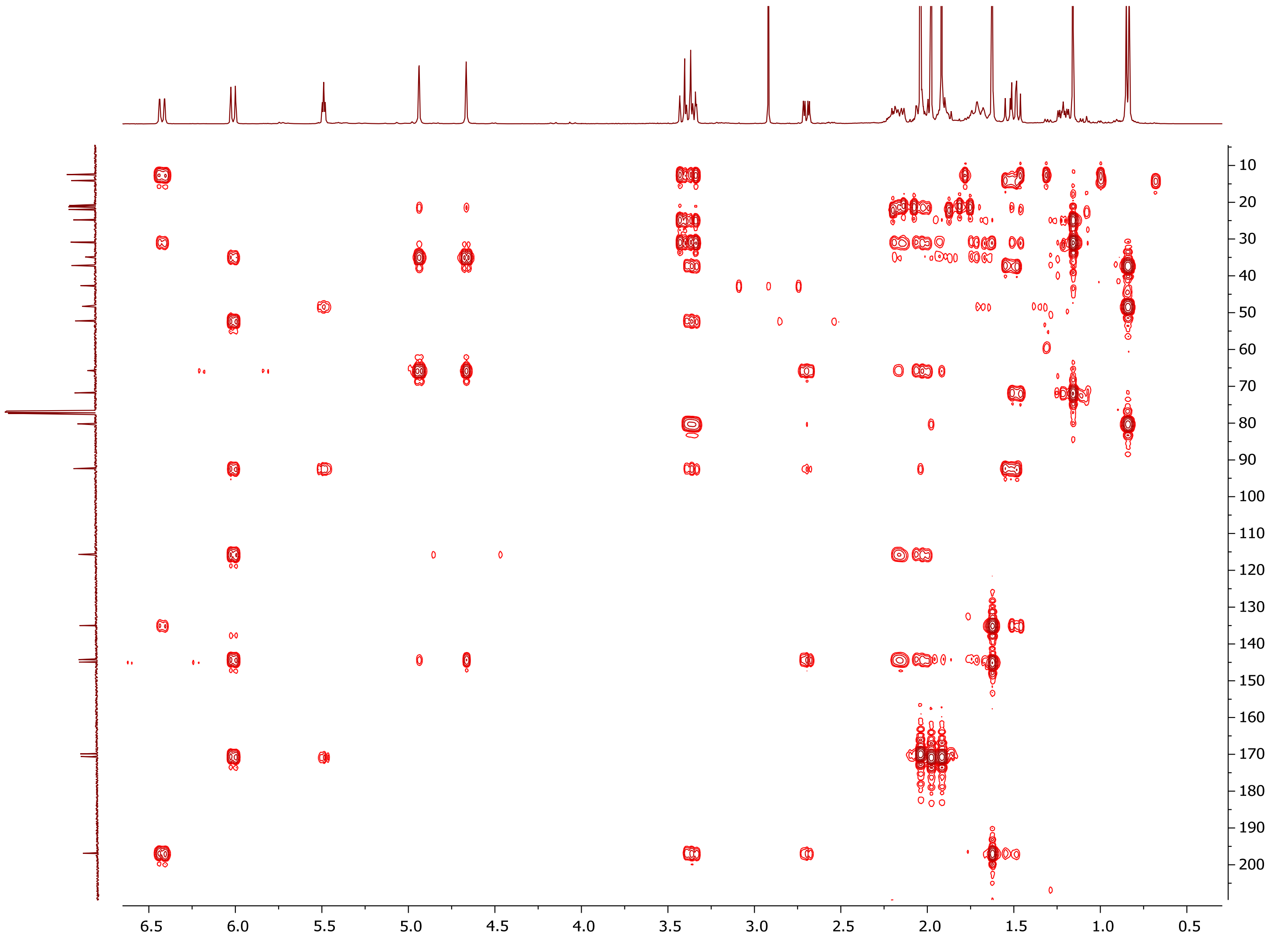

Figure S5. gHMBC spectrum of compound **3**.

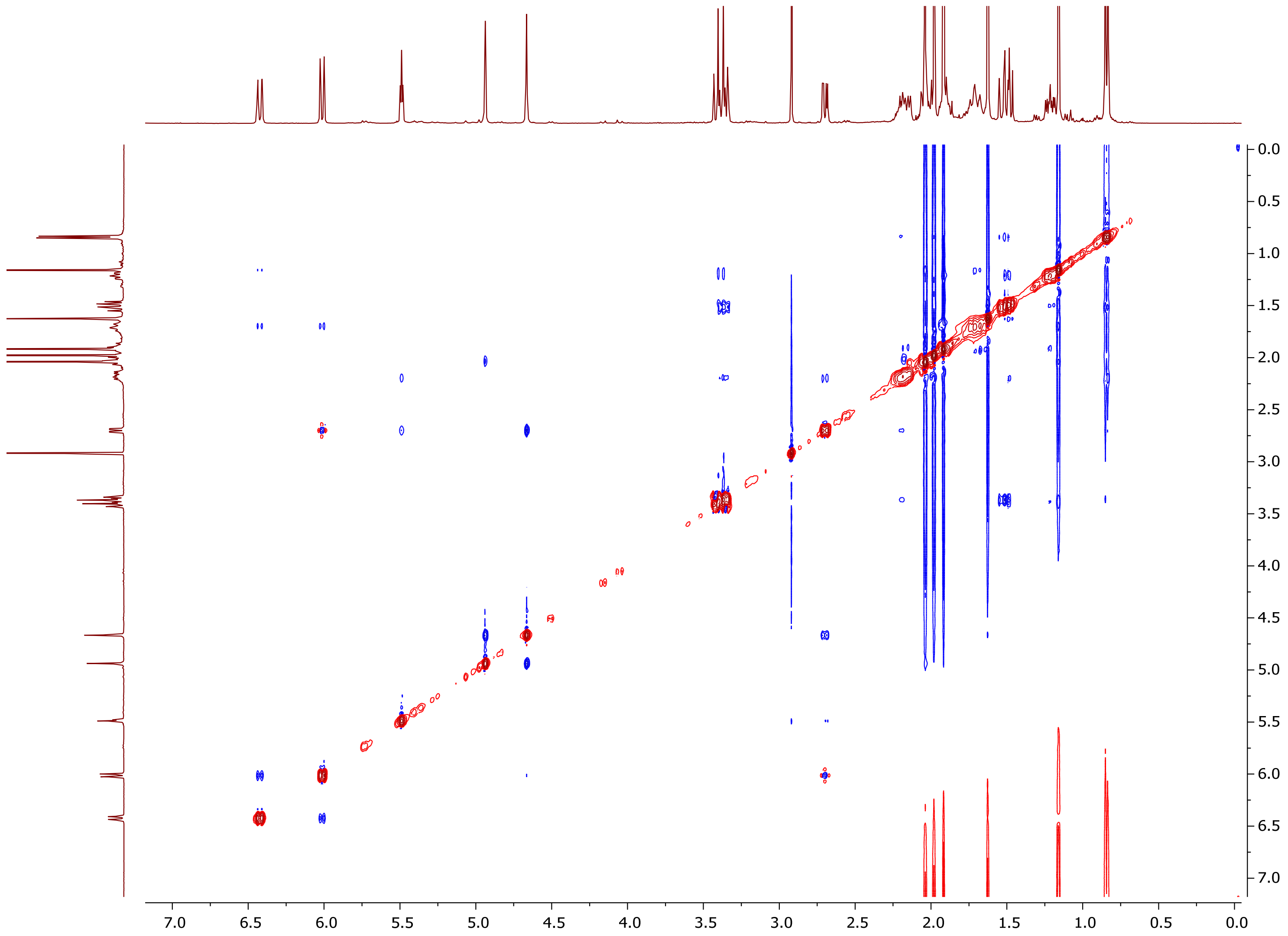

Figure S6. 2D NOESY spectrum of compound **3**.

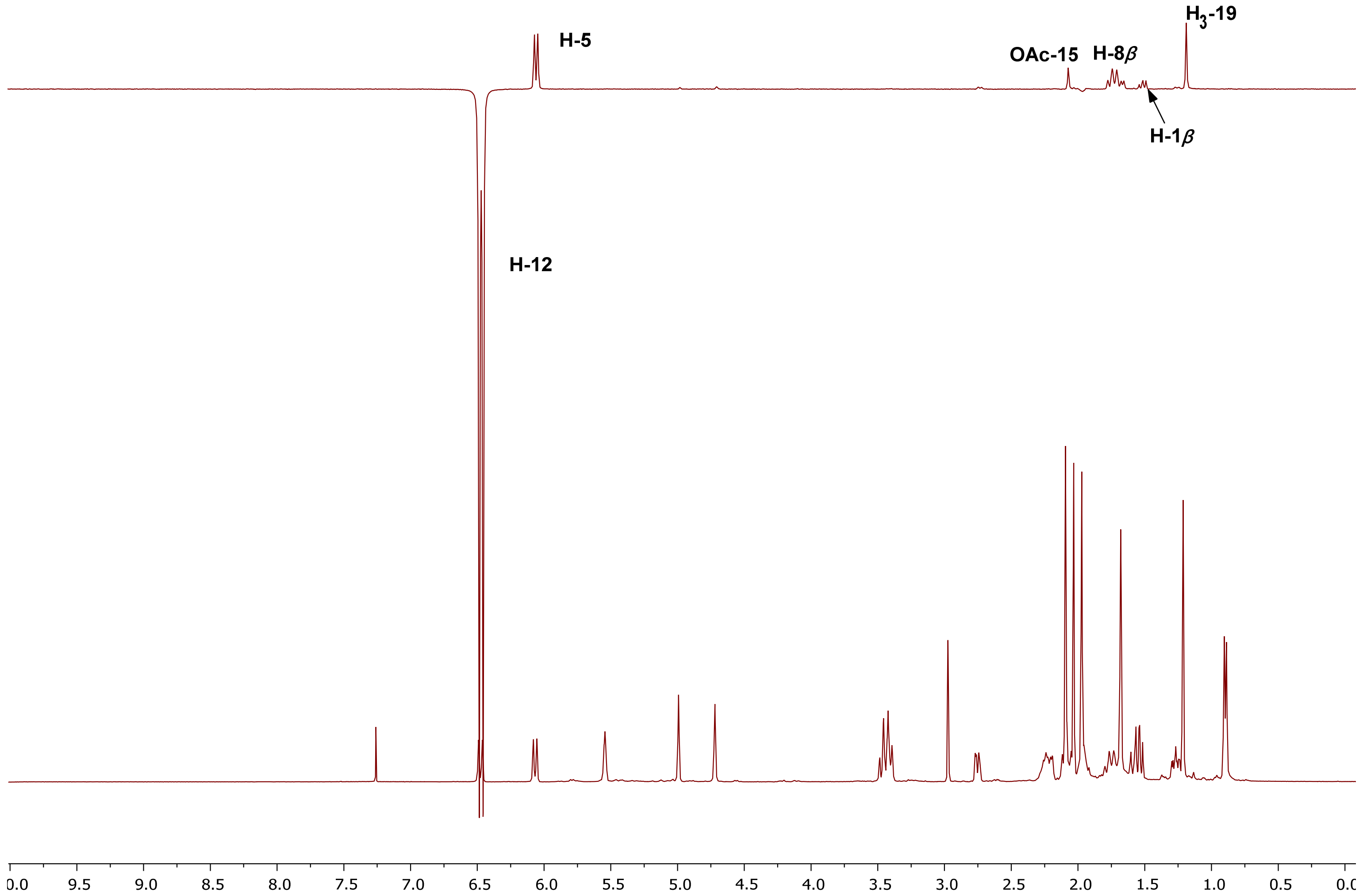

Figure S7a. 1D NOESY pectrum of compound **3**.

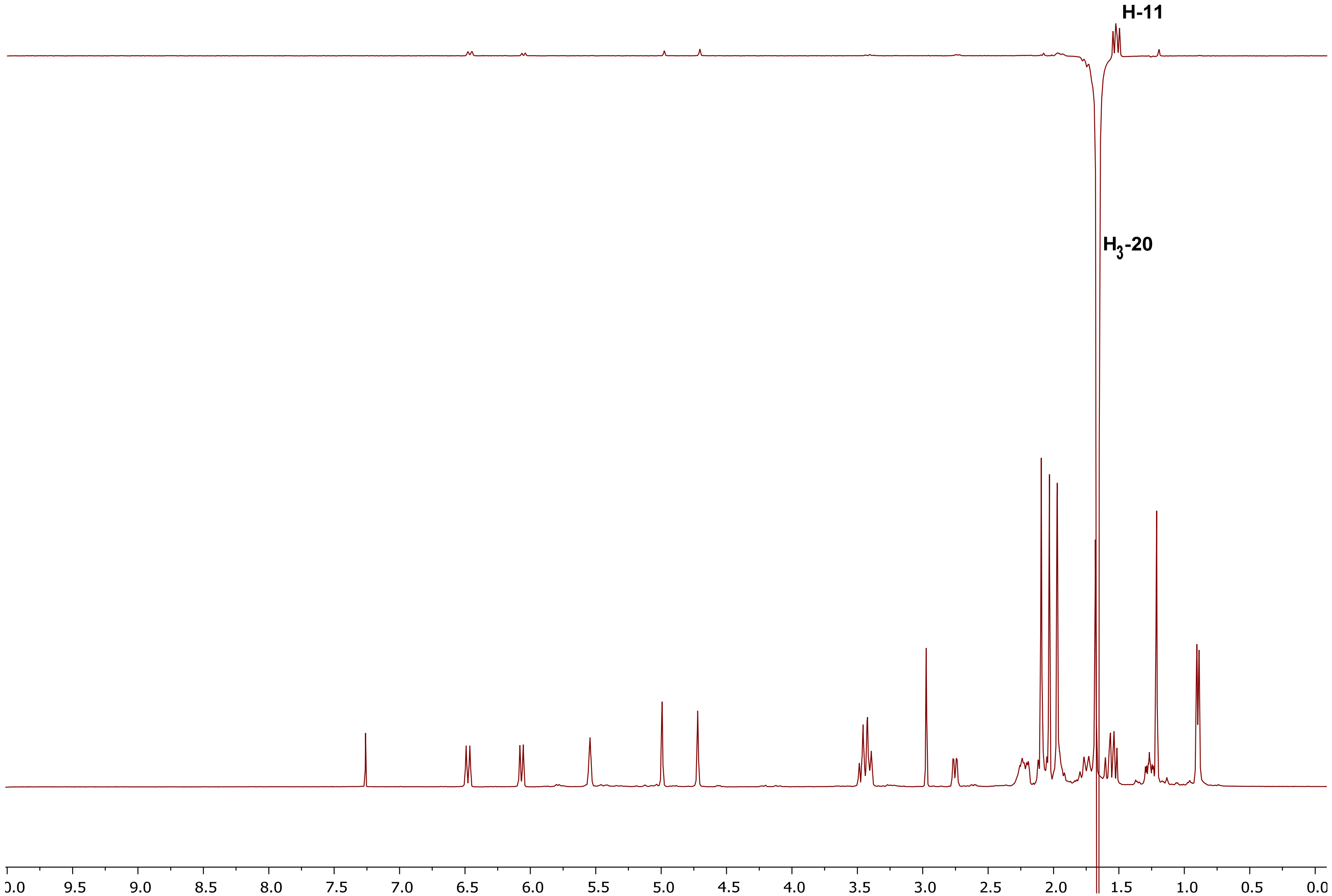

Figure S7b. 1D NOESY spectrum of compound **3**.

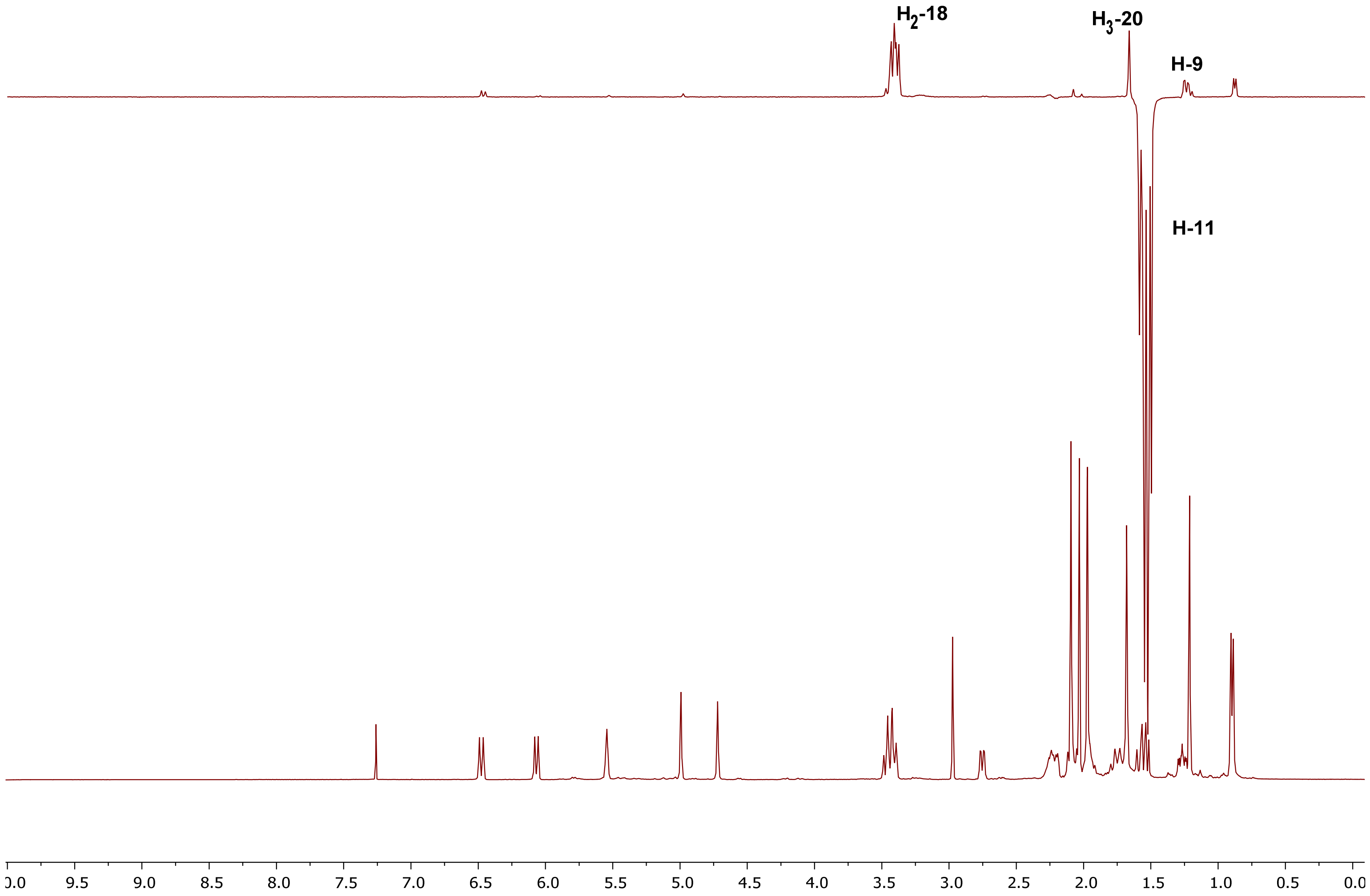

Figure S7c. 1D NOESY spectrum of compound **3**.

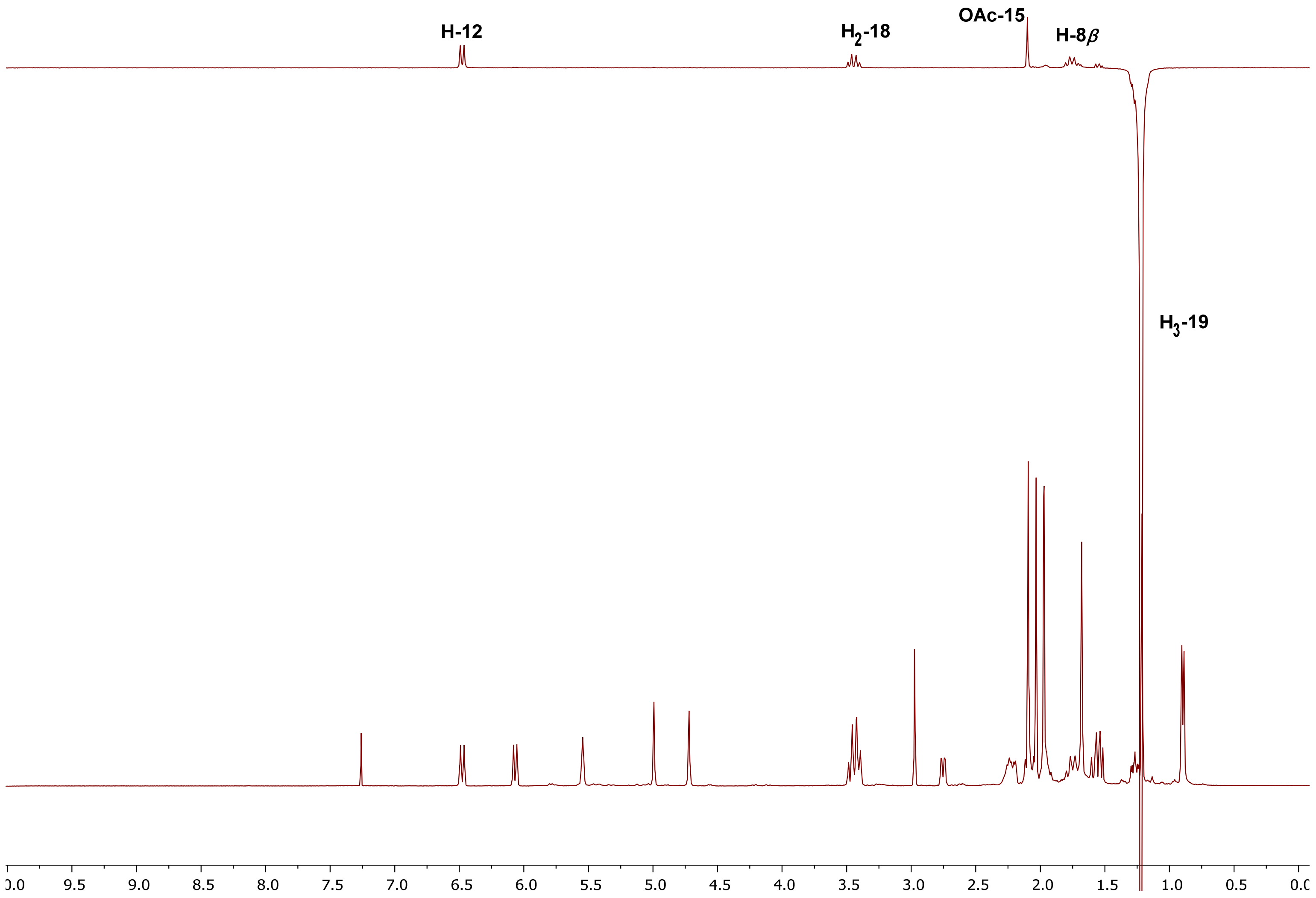

Figure S7d. 1D NOESY spectrum of compound **3**.

Elemental Composition Report

Single Mass Analysis

Tolerance = 5.0 mDa / DBE: min = -1.5, max = 80.0
Element prediction: Off
Number of isotope peaks used for i-FIT = 5

Monoisotopic Mass, Even Electron Ions
87 formula(e) evaluated with 3 results within limits (all results (up to 1000) for each mass)
Elements Used:
C: 0-30 H: 0-50 O: 0-15 23Na: 0-1
243_953_Strep-EB-12-MSe3pos 65 (1.215)

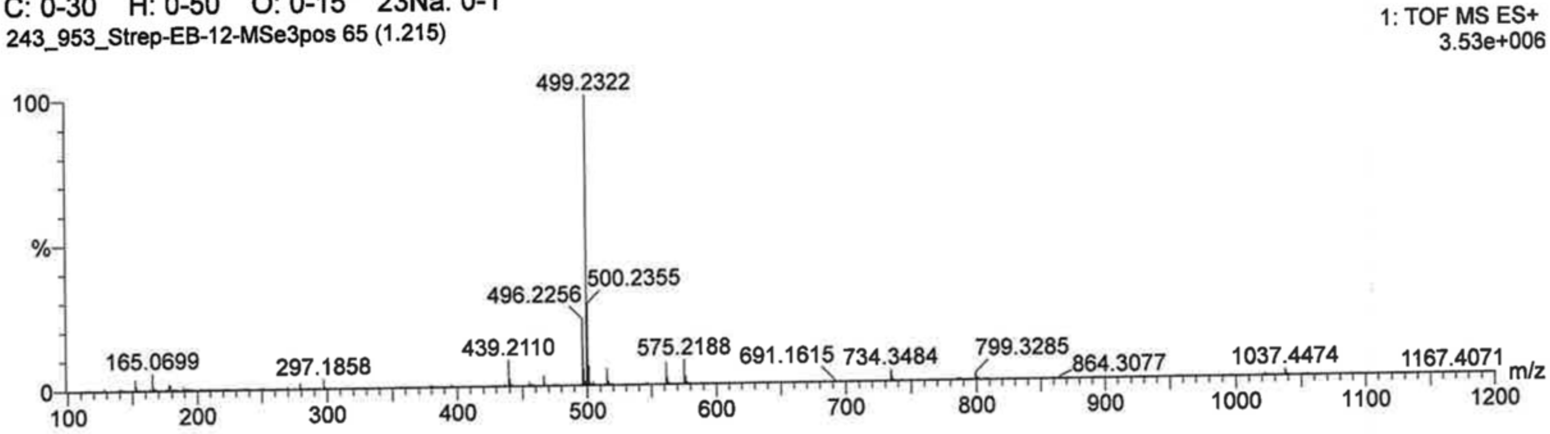

		mDa	PPM	DBE				
Minimum:				-1.5				
Maximum:		5.0	10.0	80.0				

Mass	Calc. Mass	mDa	PPM	DBE	i-FIT	Norm	Conf(%)	Formula
499.2322	499.2308	1.4	2.8	8.5	1690.9	0.198	82.03	C26 H36 O8 23Na
	499.2332	-1.0	-2.0	11.5	1692.4	1.719	17.92	C28 H35 O8
	499.2367	-4.5	-9.0	-0.5	1698.2	7.489	0.06	C19 H40 O13 23Na

Figure S8. HRMS of compound **3**.

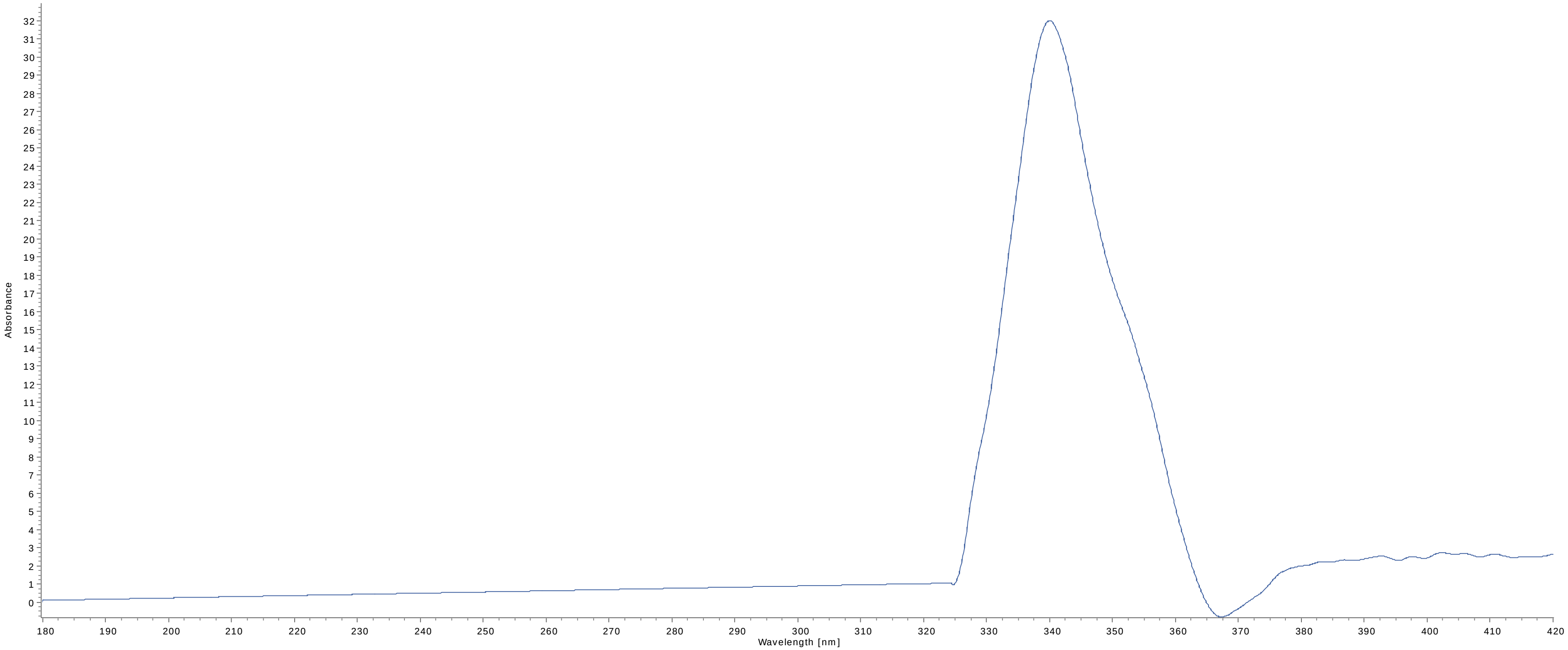

Figure S9. ECD of compound **3**.

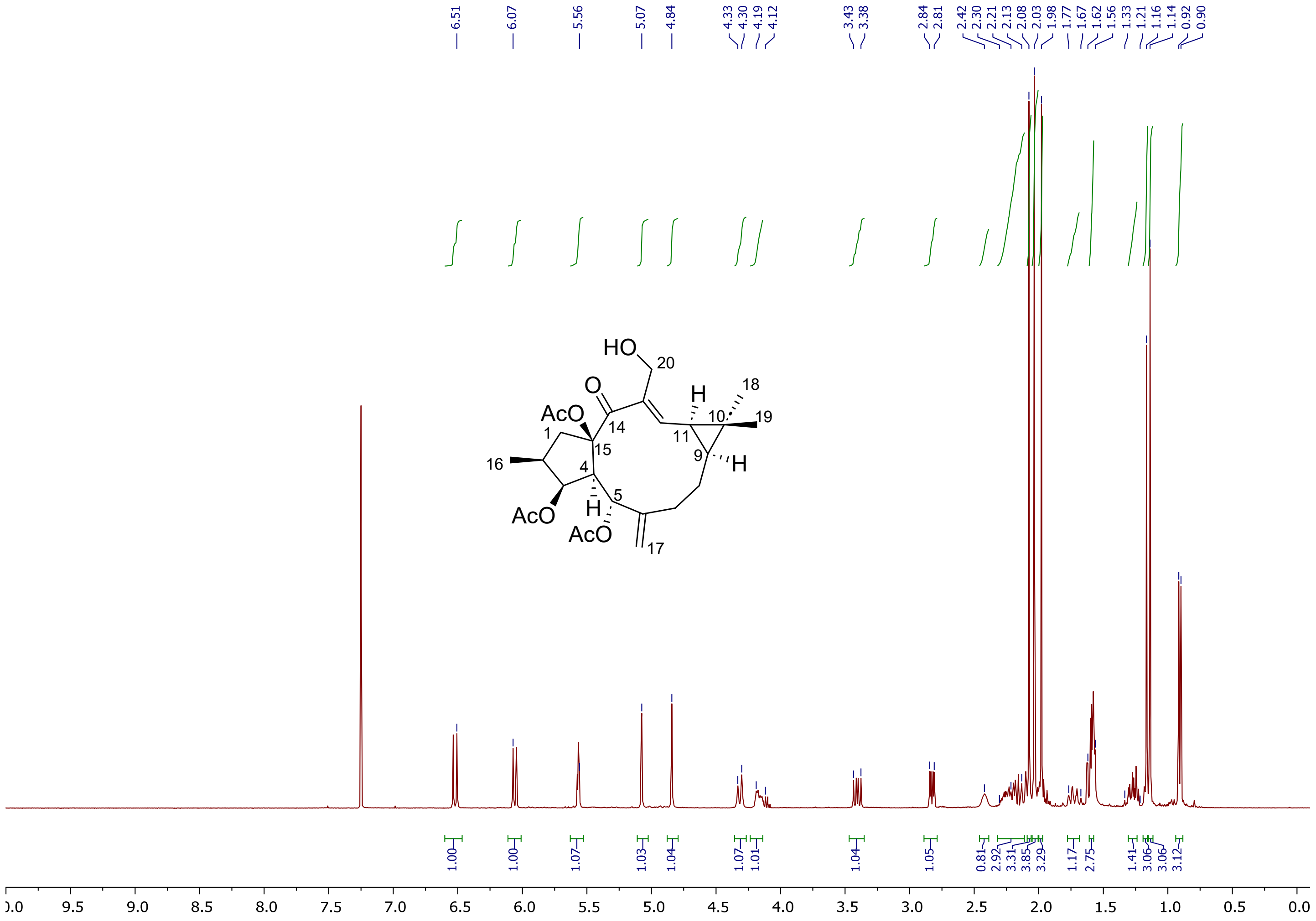

Figure S10. ^{1}H NMR spectrum (400 MHz) of compound **4** in $CDCl_3$.

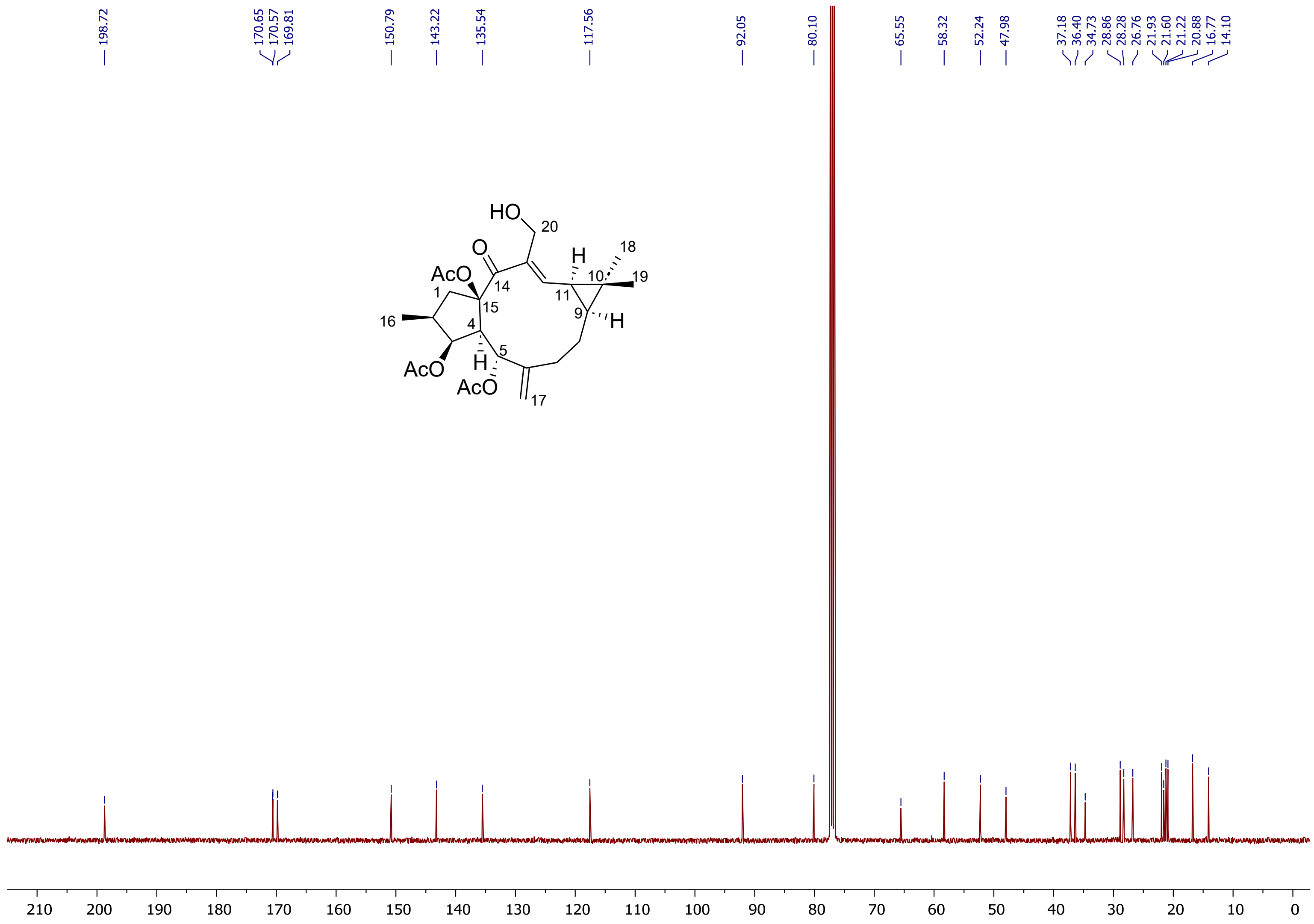

Figure S11. ^{13}C NMR spectrum (100 MHz) of compound **4** in $CDCl_3$.

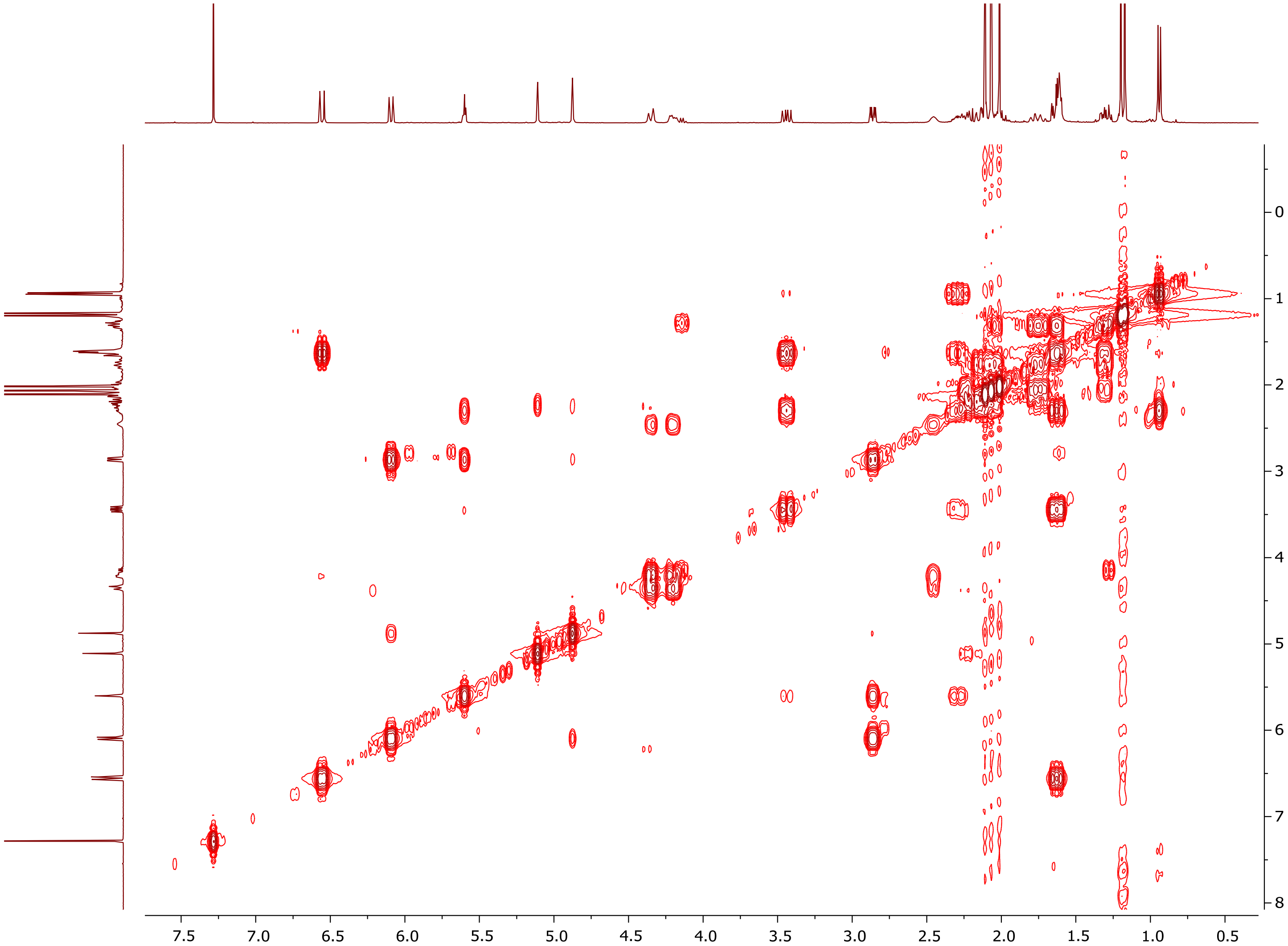

Figure S12. gCOSY spectrum of compound **4**.

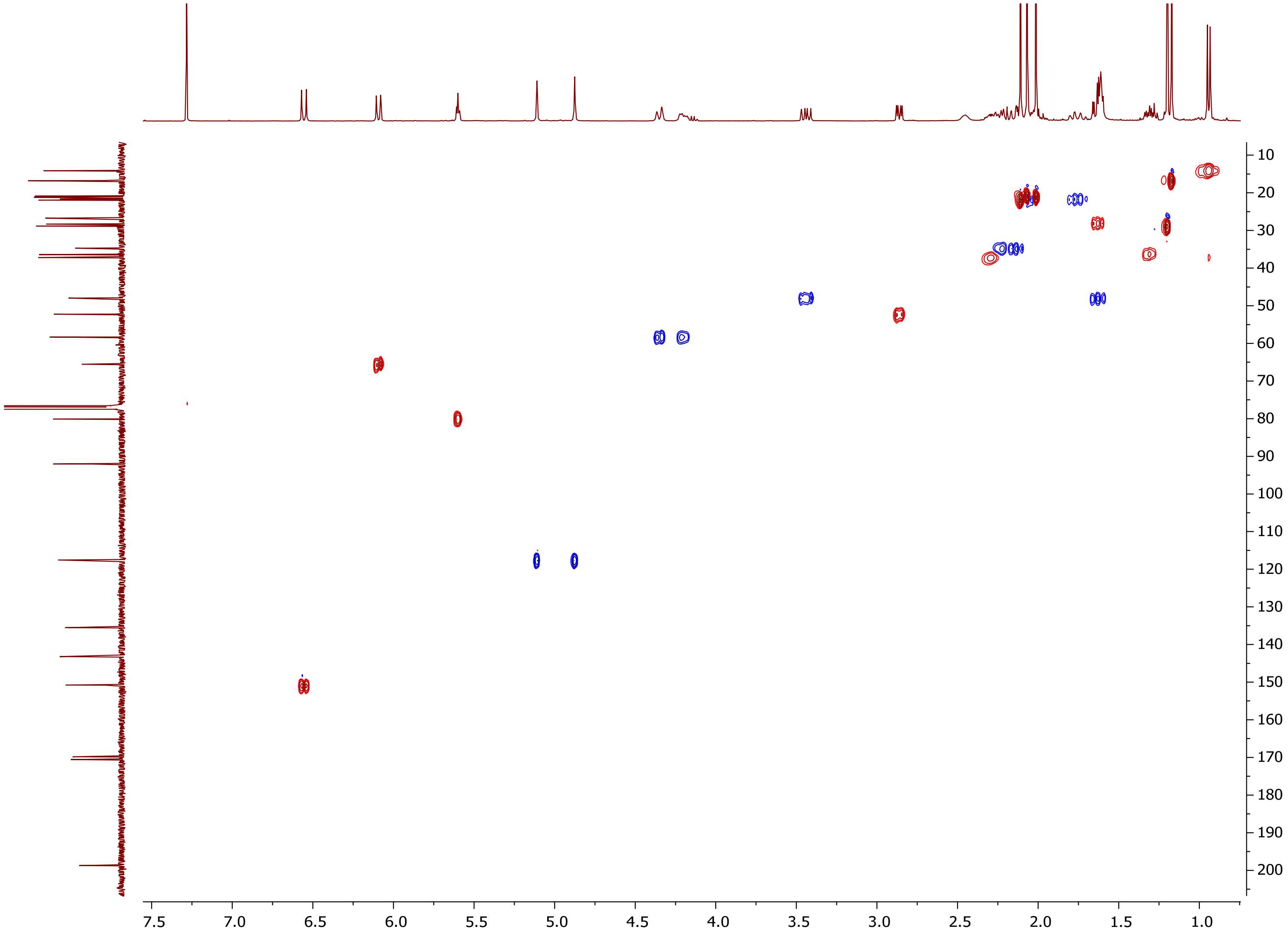

Figure S13. gHSQC spectrum of compound **4**.

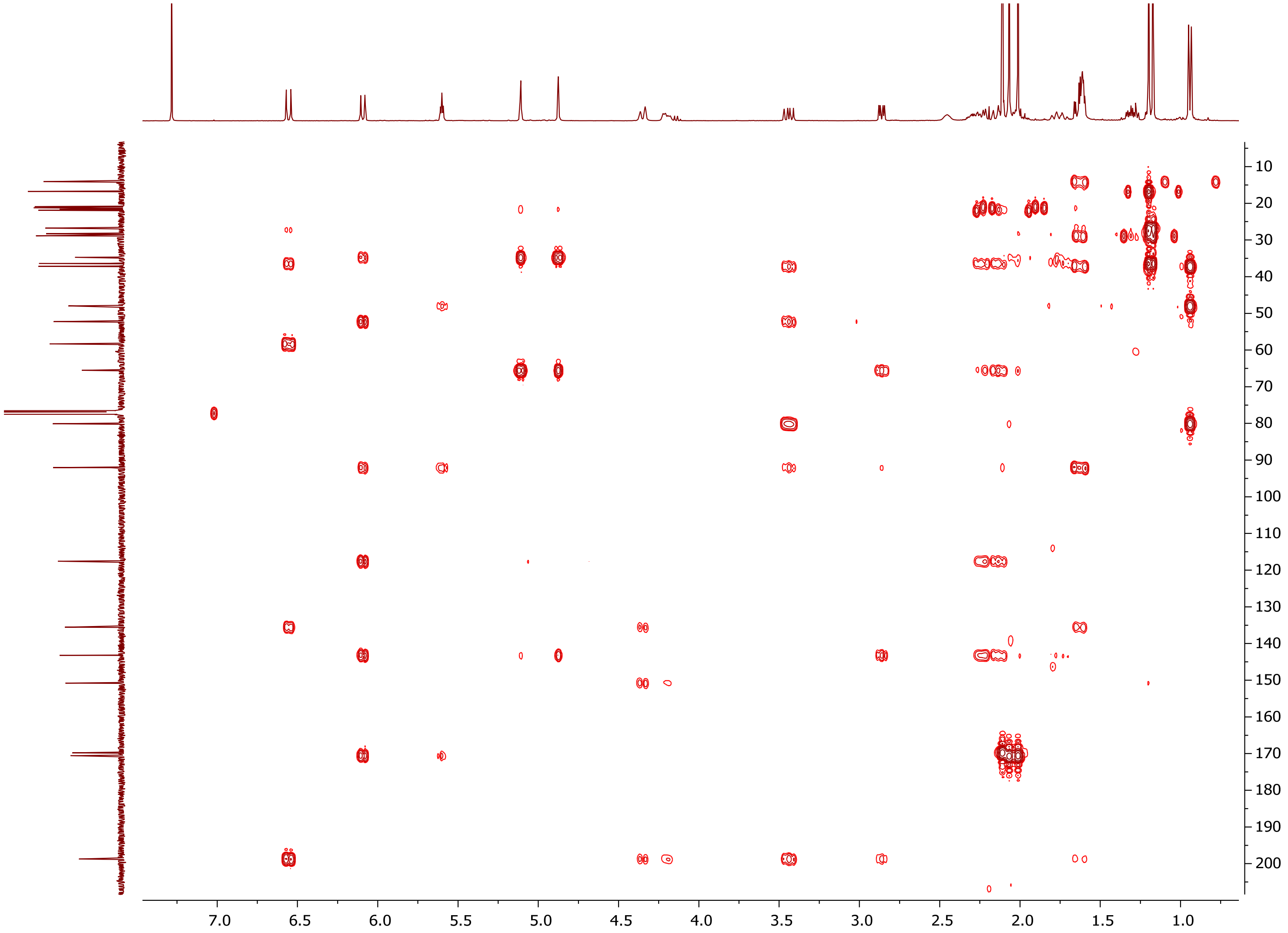

Figure S14. gHMBC spectrum of compound **4**.

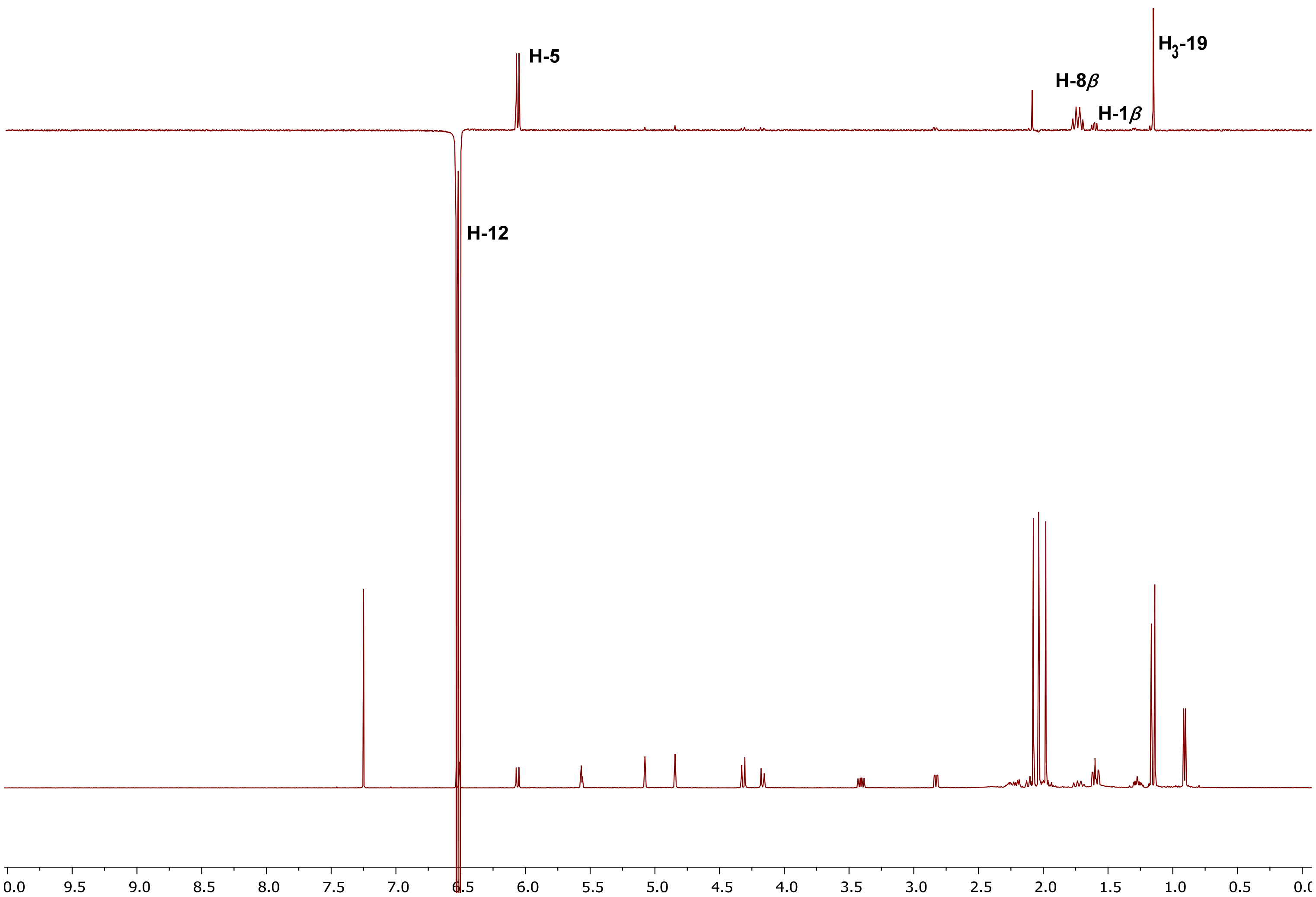

Figure S15a. 1D NOESY spectrum of compound **4**.

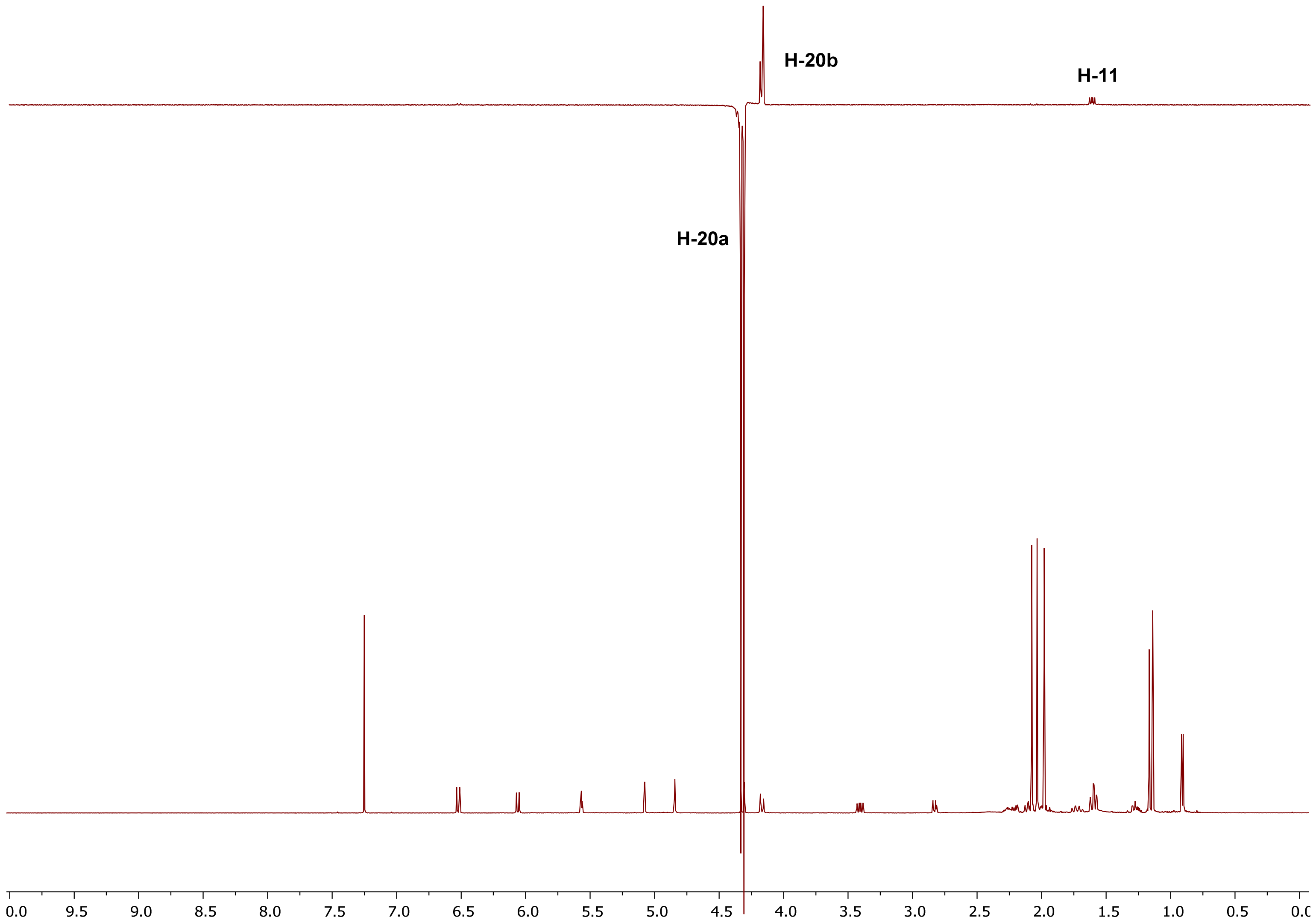

Figure S15b. 1D NOESY spectrum of compound **4**.

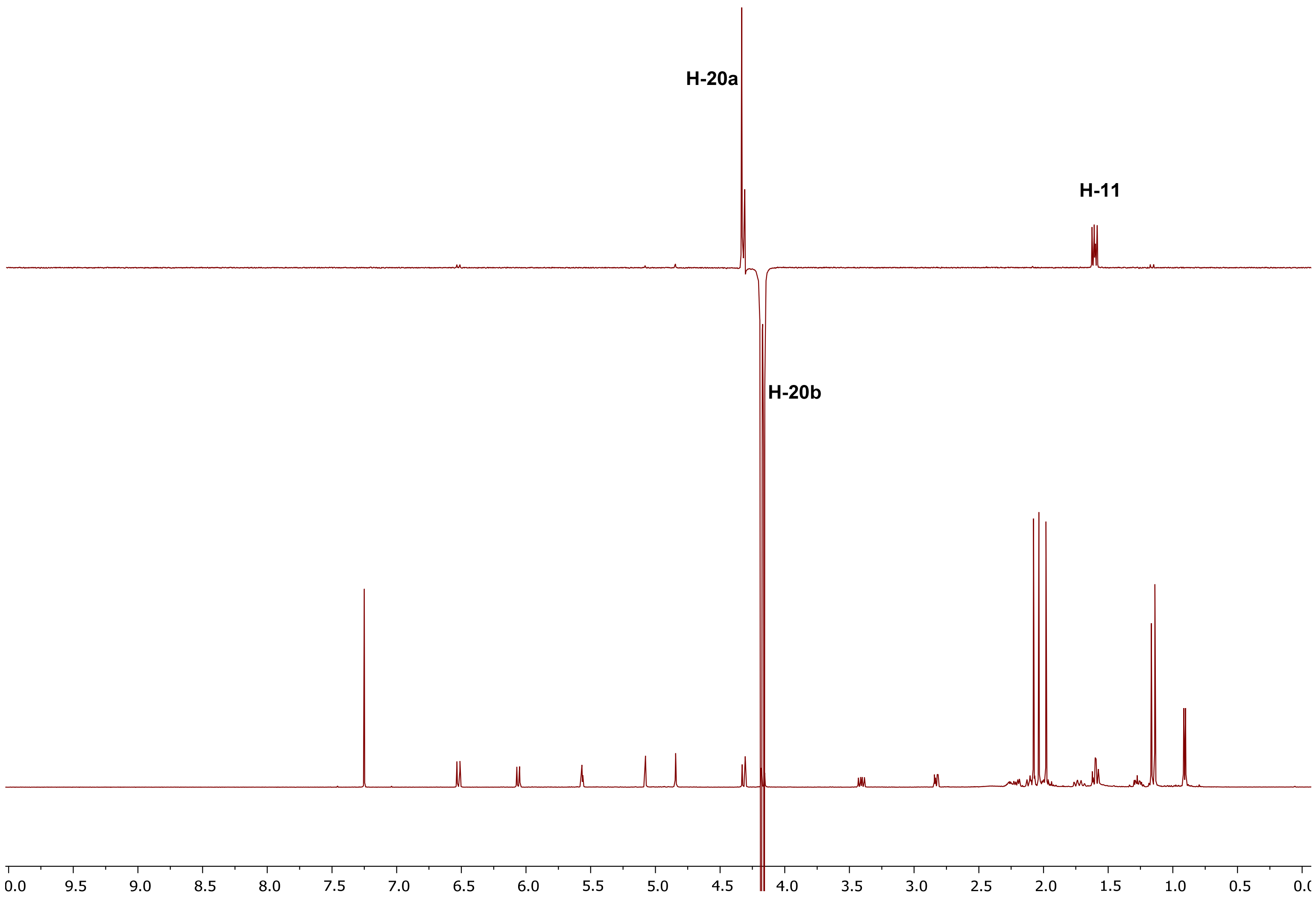

Figure S15c. 1D NOESY spectrum of compound **4**.

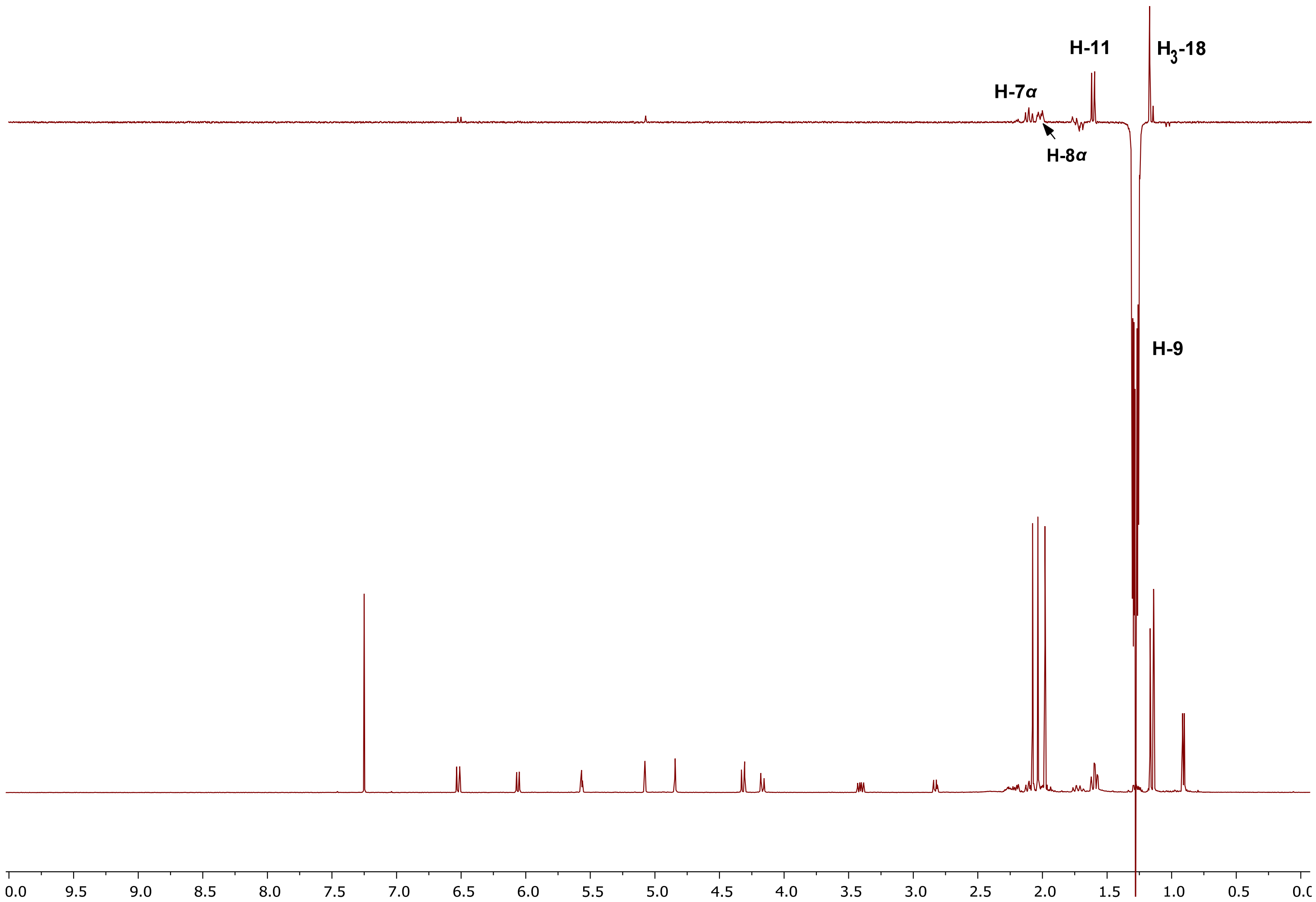

Figure S15d. 1D NOESY spectrum of compound **4**.

Elemental Composition Report

Single Mass Analysis

Tolerance = 5.0 mDa / DBE: min = -1.5, max = 80.0
Element prediction: Off
Number of isotope peaks used for i-FIT = 5

Monoisotopic Mass, Even Electron Ions
87 formula(e) evaluated with 2 results within limits (all results (up to 1000) for each mass)
Elements Used:
C: 0-30 H: 0-50 O: 0-15 23Na: 0-1

246_1009_Strep-EB12- MSe3pos 148 (2.741)

1: TOF MS ES+
3.42e+005

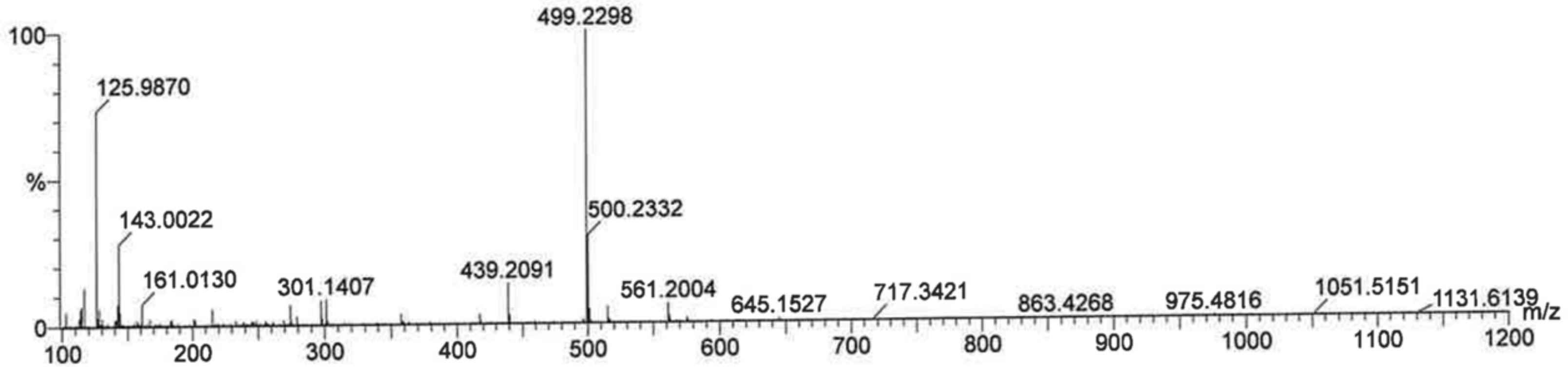

Mass	Calc. Mass	mDa	PPM	DBE	i-FIT	Norm	Conf(%)	Formula
Minimum:				-1.5				
Maximum:		5.0	10.0	80.0				
499.2298	499.2308	-1.0	-2.0	8.5	1285.1	0.063	93.85	C26 H36 O8 23Na
	499.2332	-3.4	-6.8	11.5	1287.8	2.789	6.15	C28 H35 O8

Figure S16. HRMS of compound **4**.

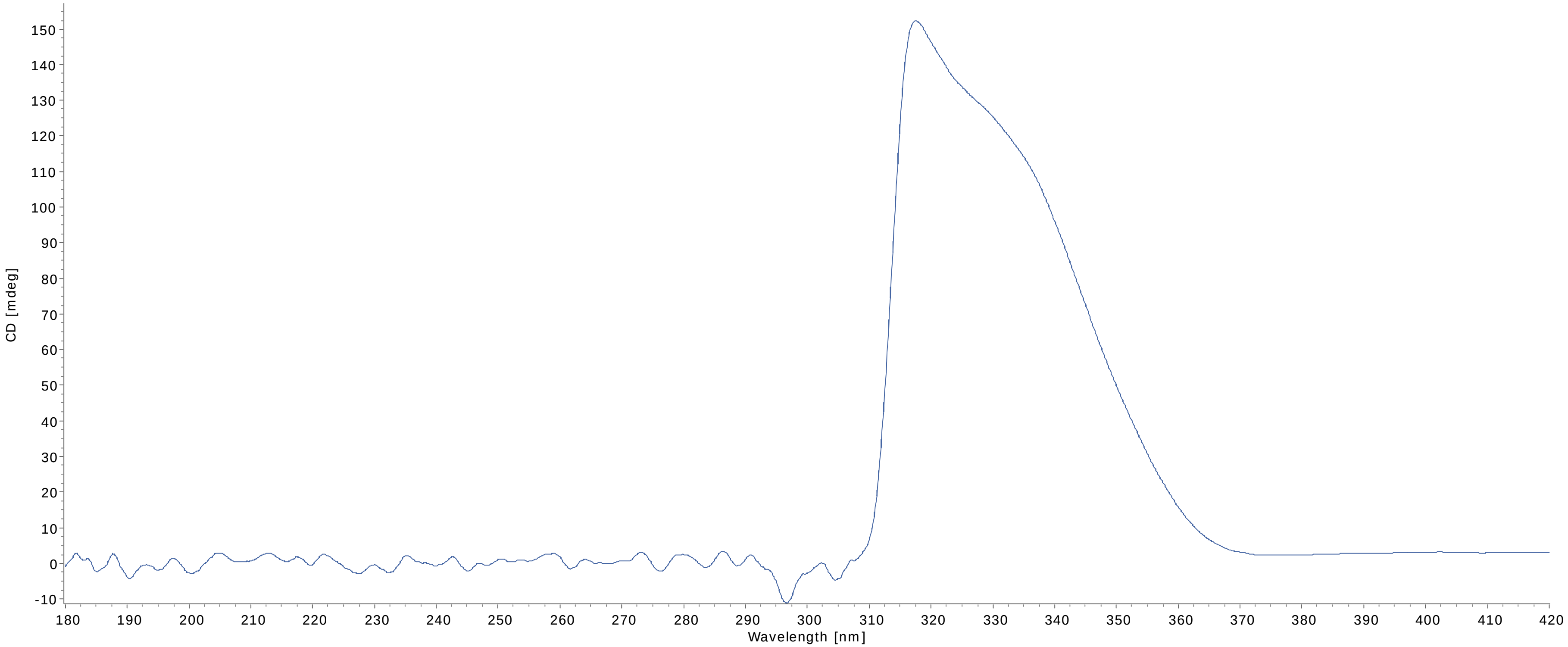

Figure S17. ECD of compound **4**.

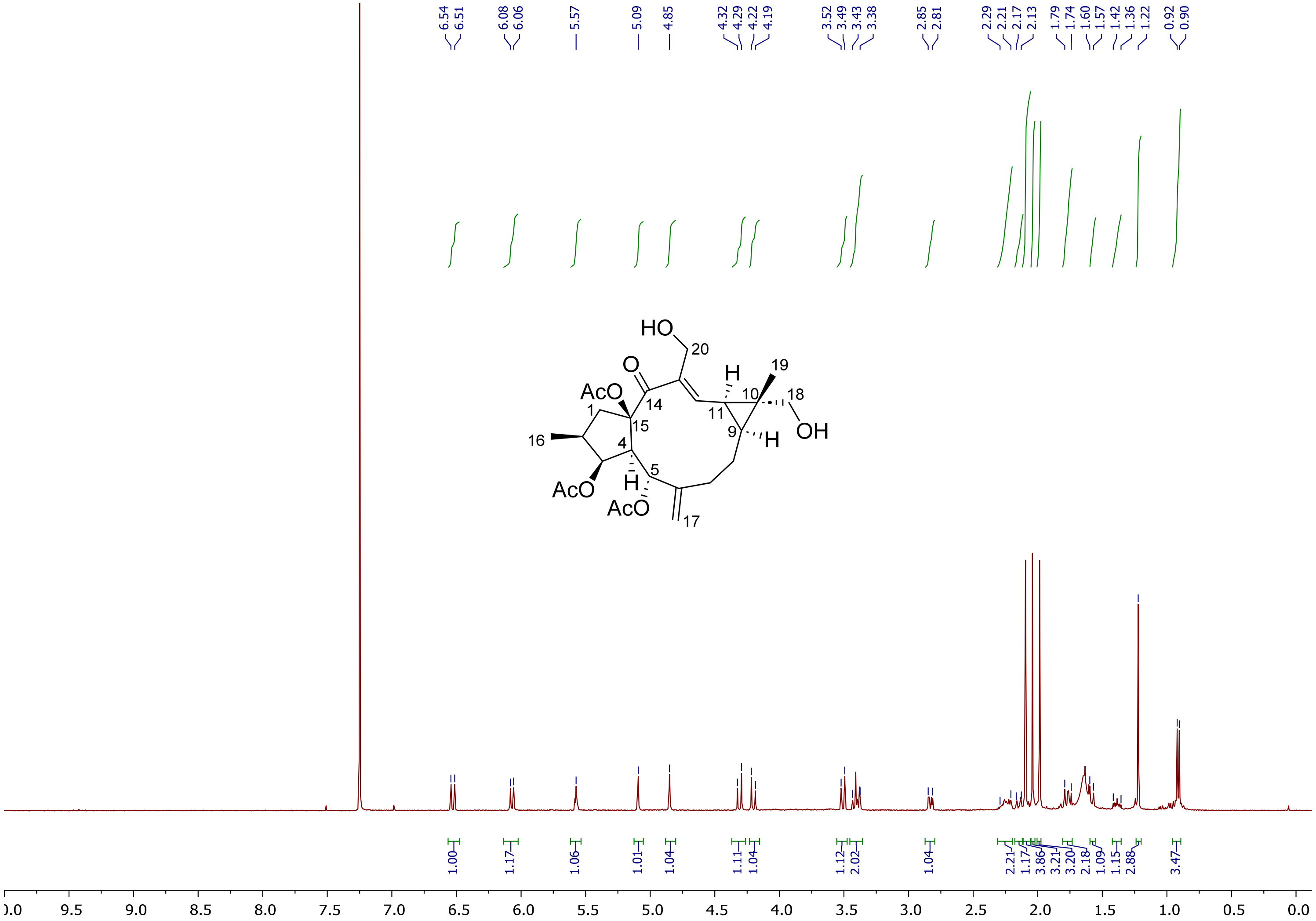

Figure S18. ^{1}H NMR spectrum (400 MHz) of compound **5** in $CDCl_3$.

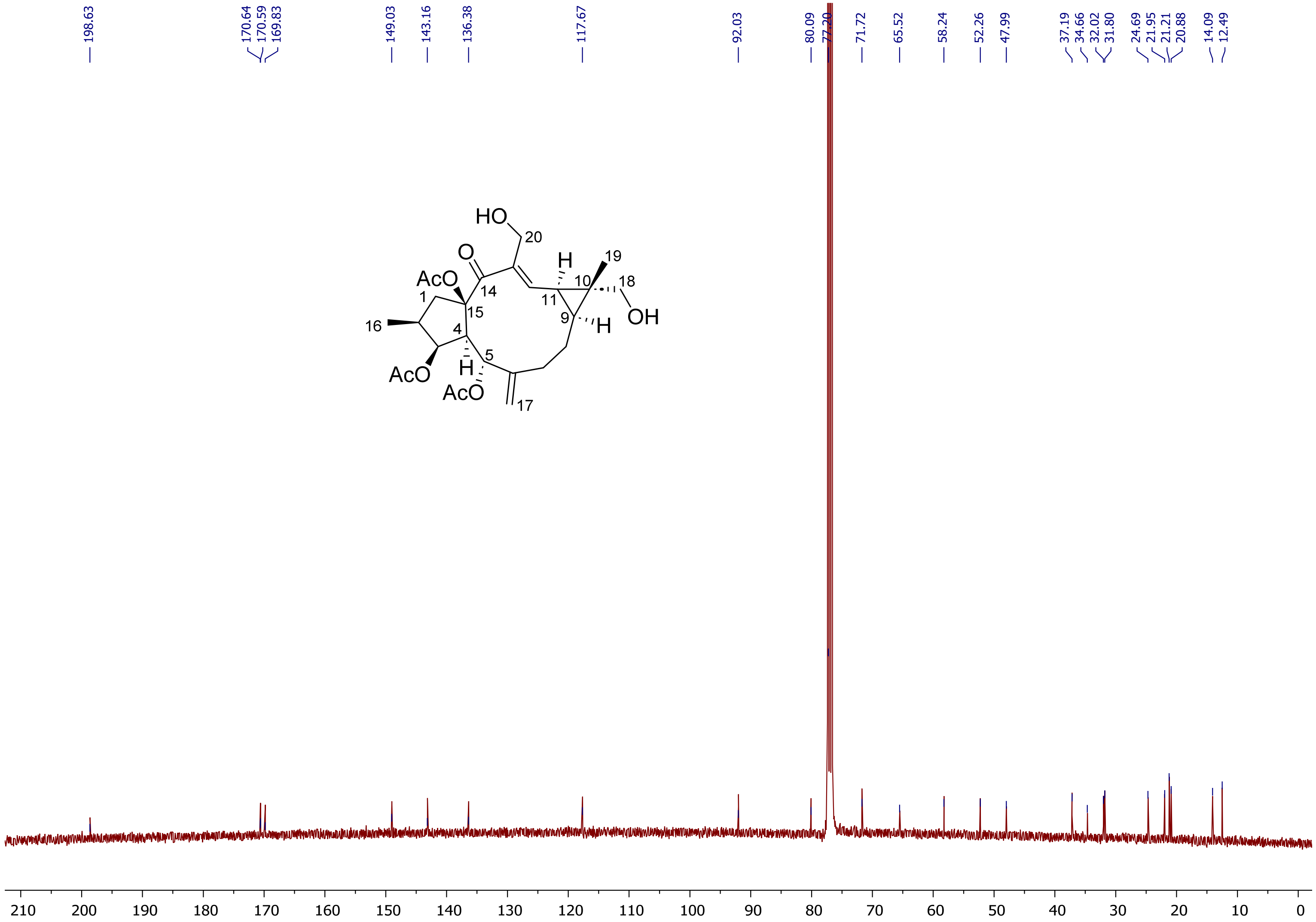

Figure S19. ^{13}C NMR spectrum (100 MHz) of compound **5** in $CDCl_3$.

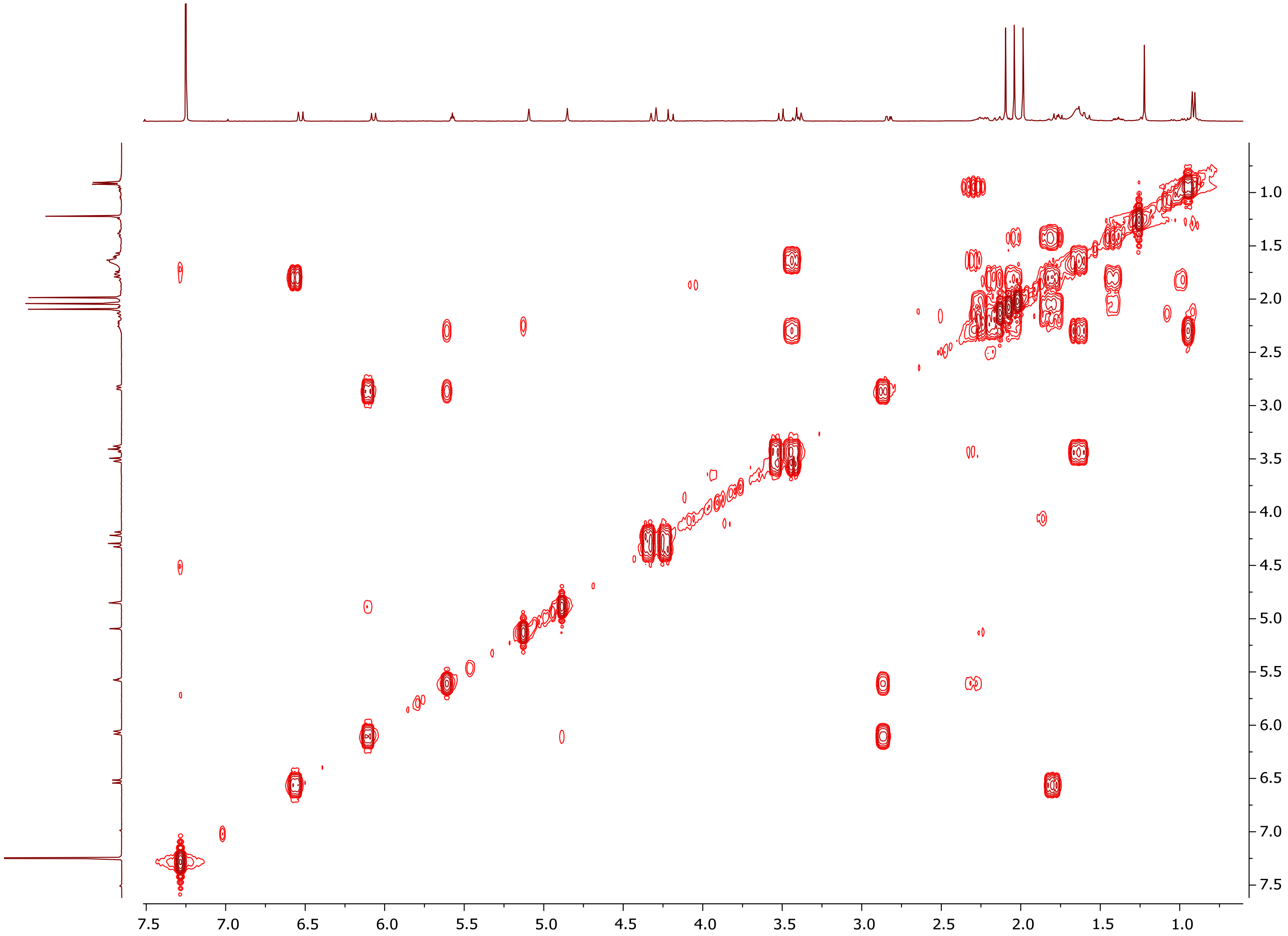

Figure S20. gCOSY spectrum of compound **5**.

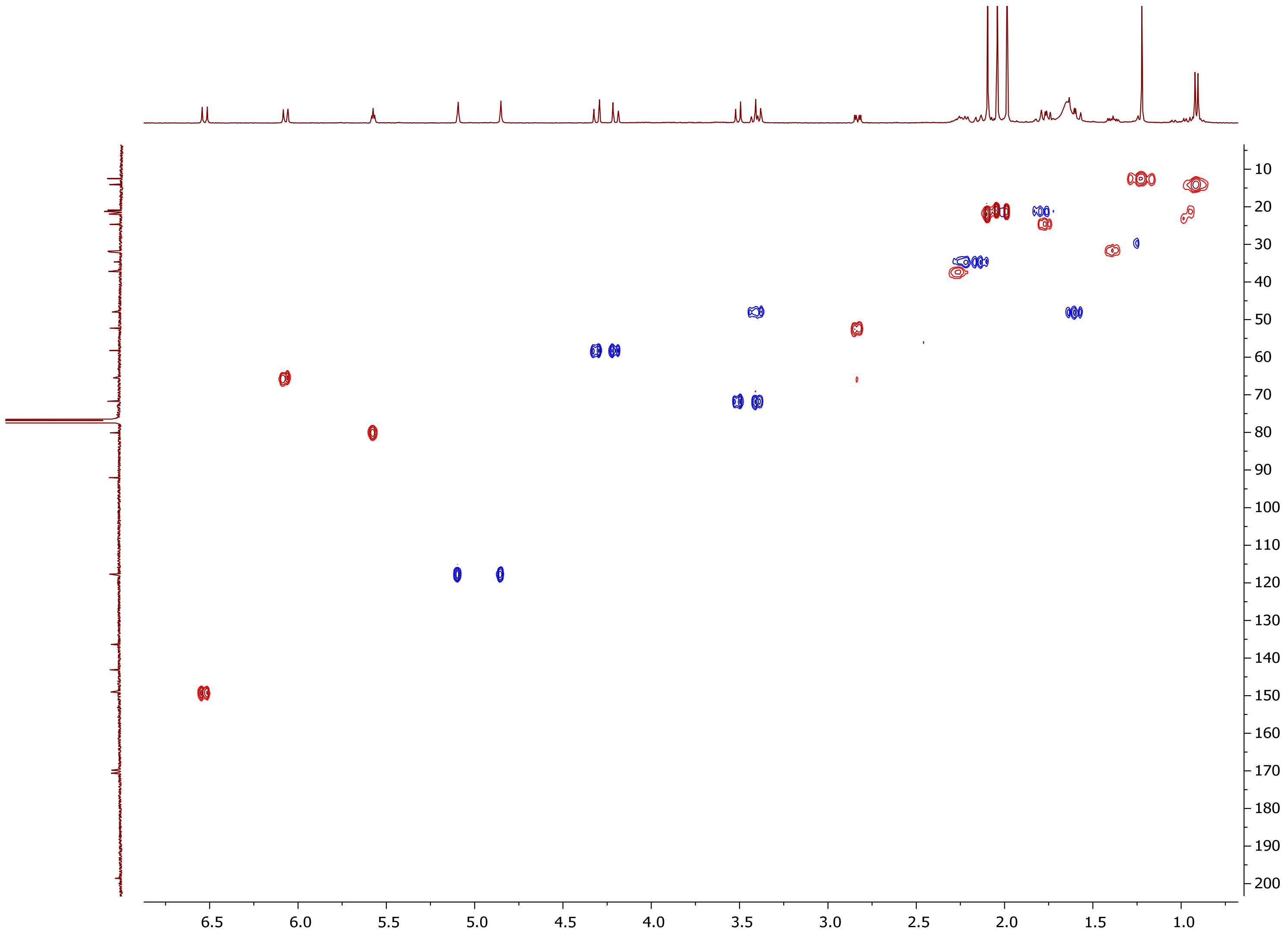

Figure S21. gHSQC spectrum of compound **5**.

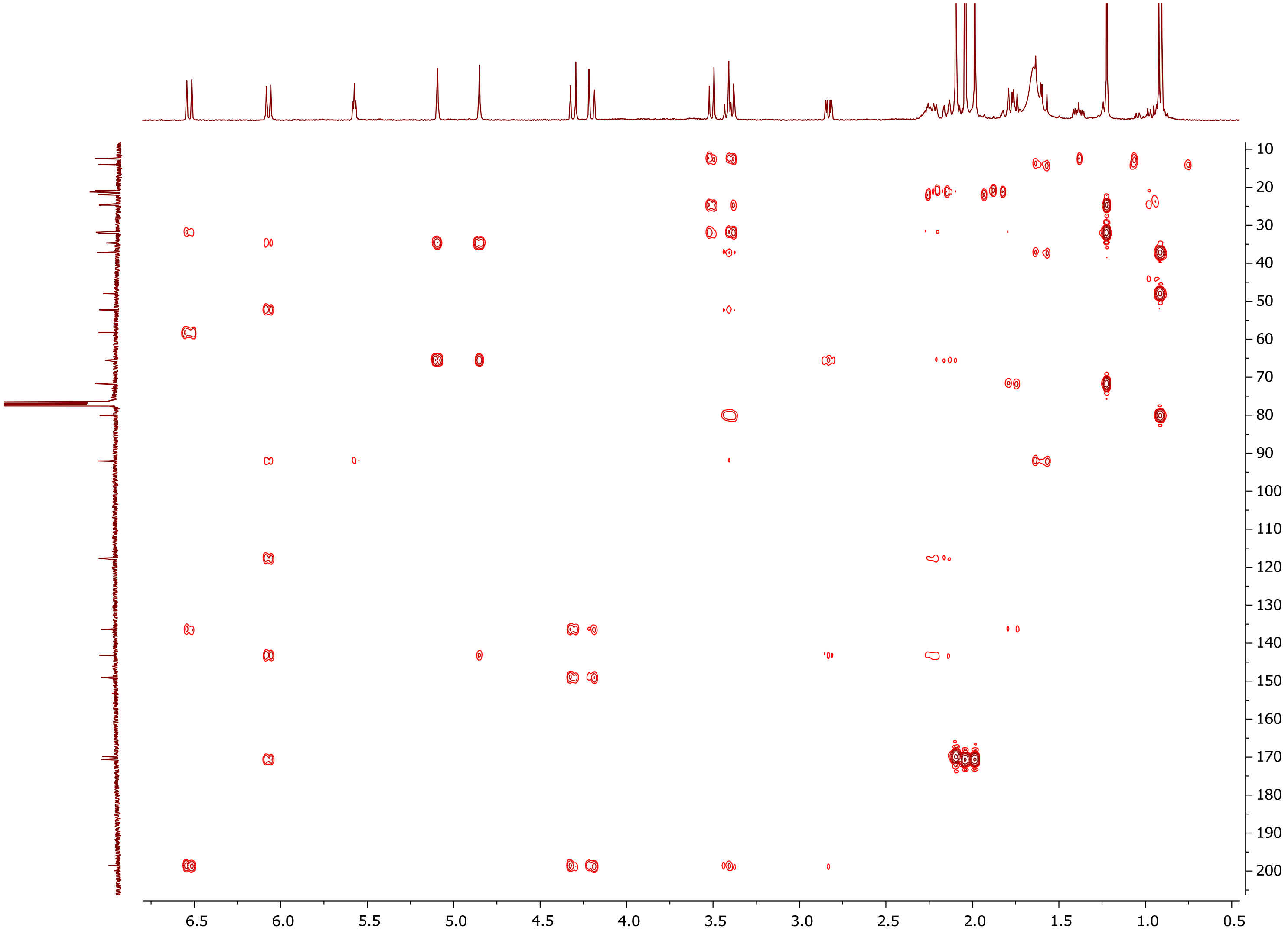

Figure S22. gHMBC spectrum of compound **5**.

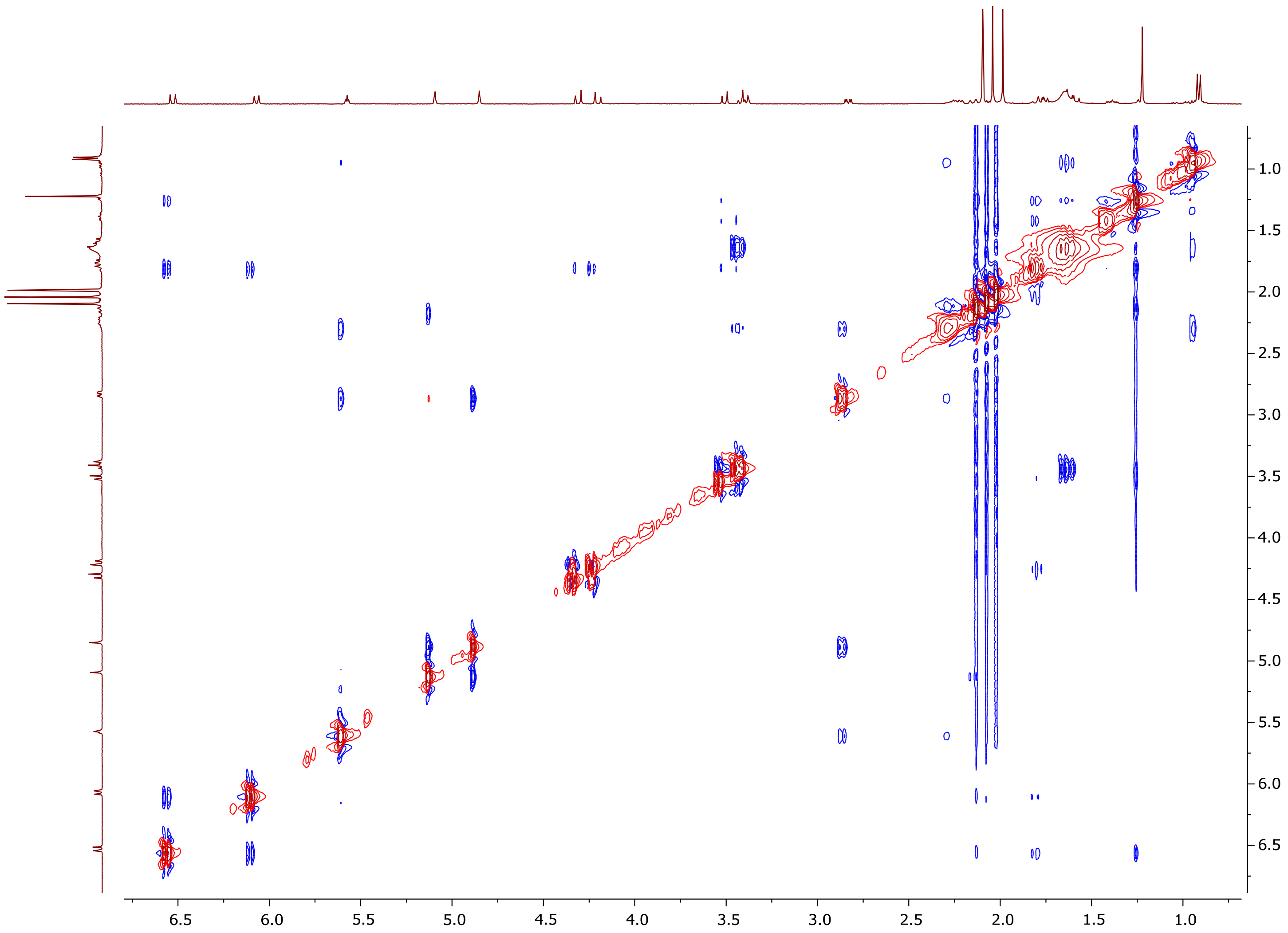

Figure S23. 2D NOESY spectrum of compound **5**.

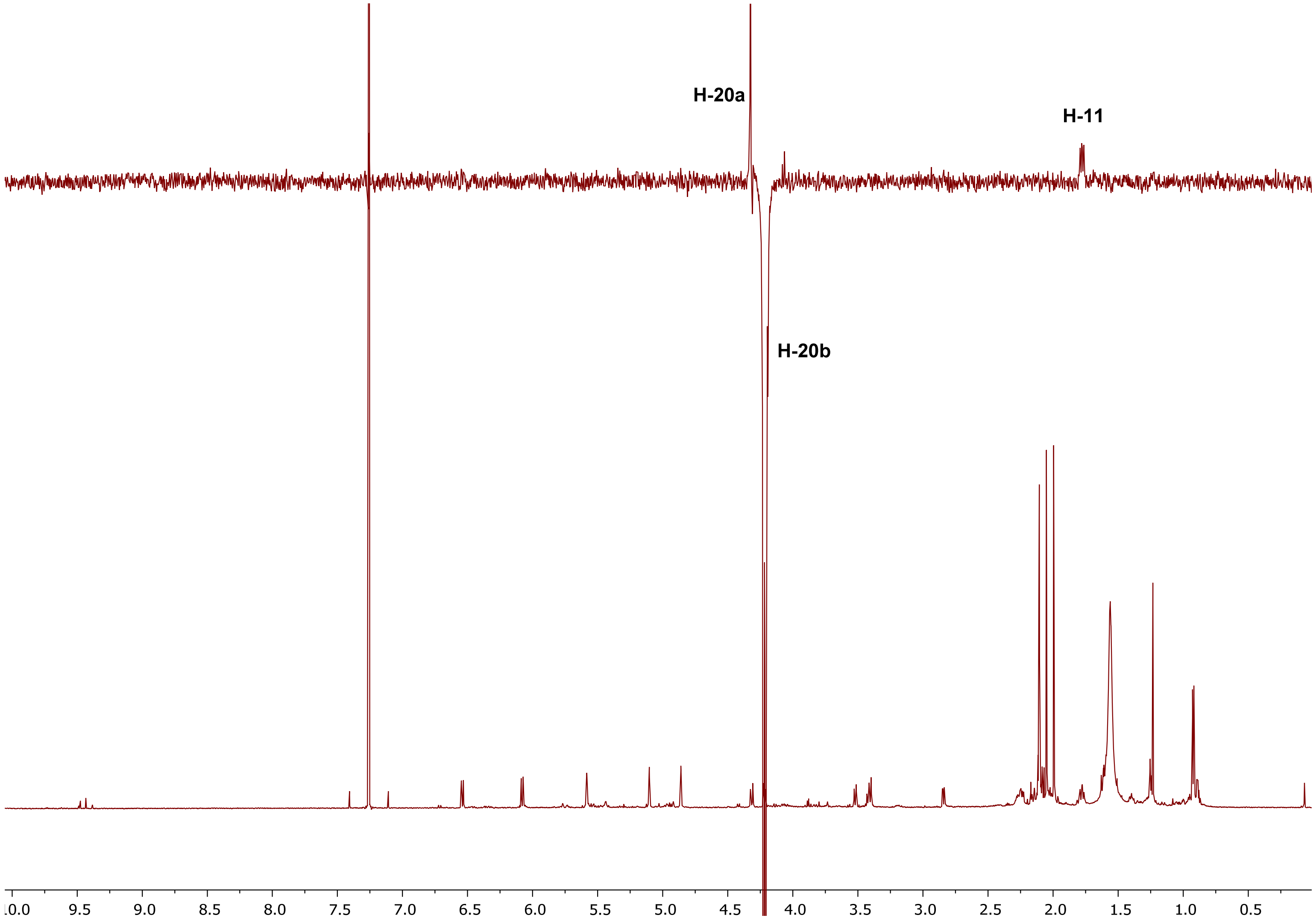

Figure S24a. 1D NOESY spectrum of compound **5**.

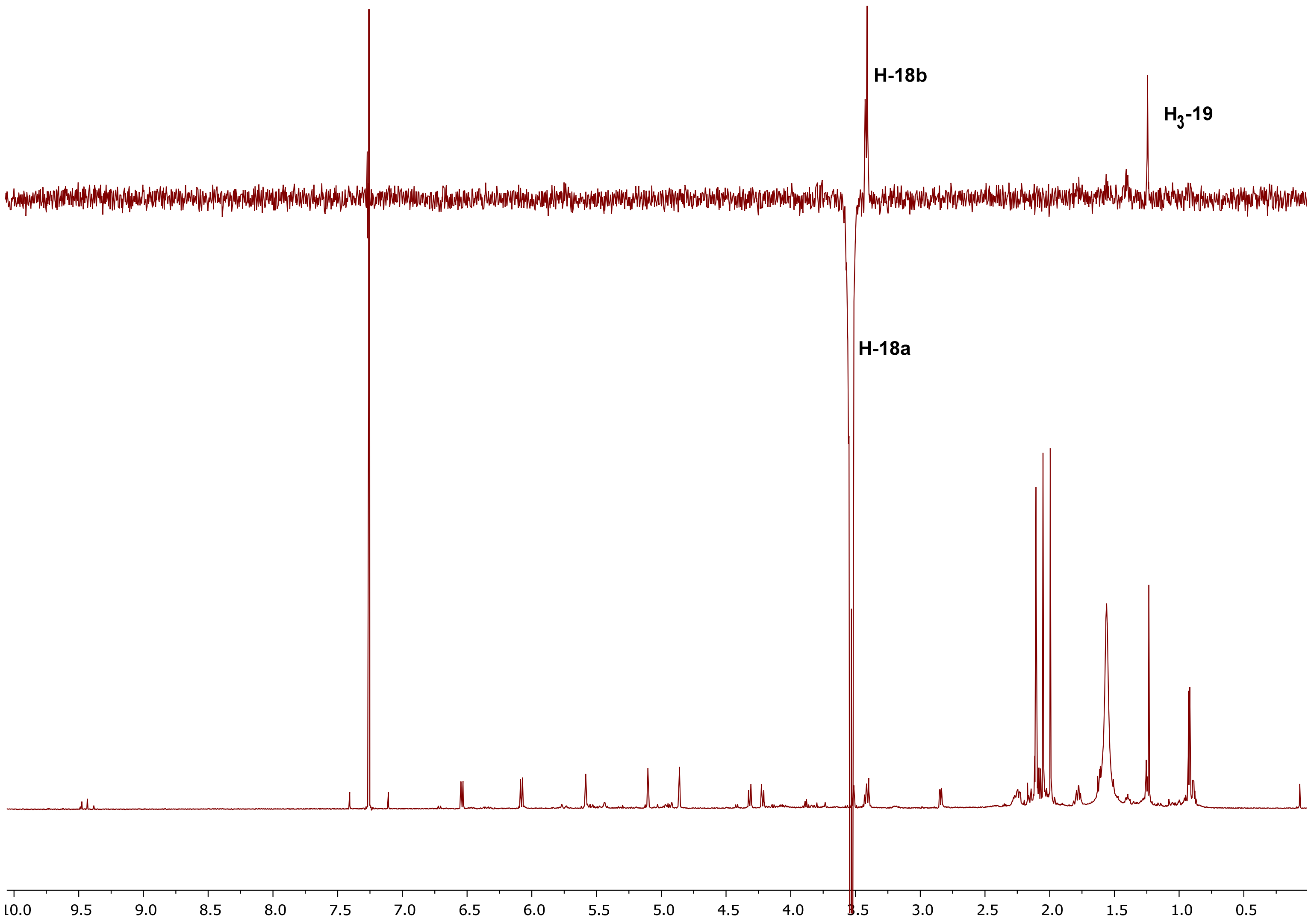

Figure S24b. 1D NOESY spectrum of compound **5**.

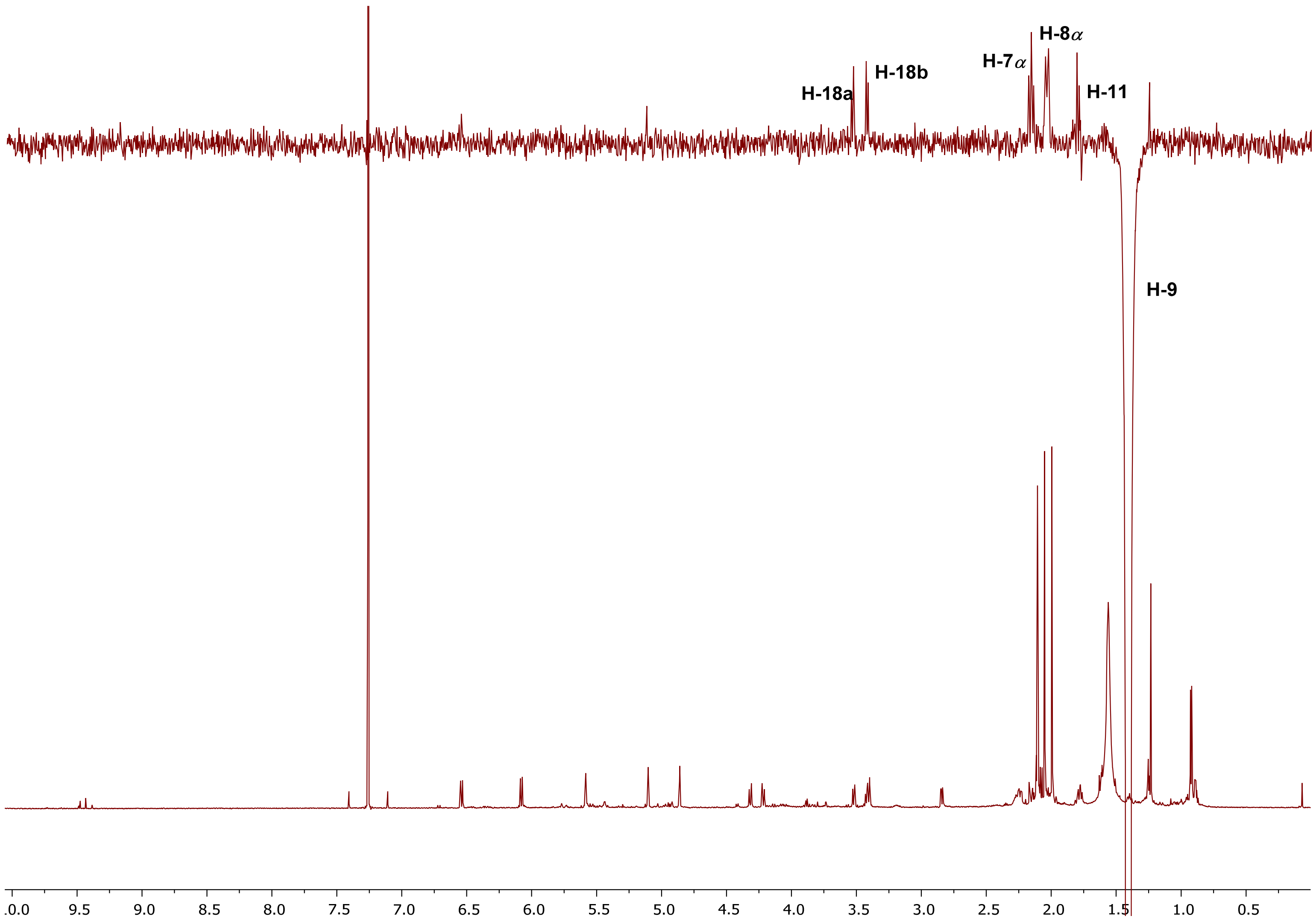

Figure S24c. 1D NOESY spectrum of compound **5**.

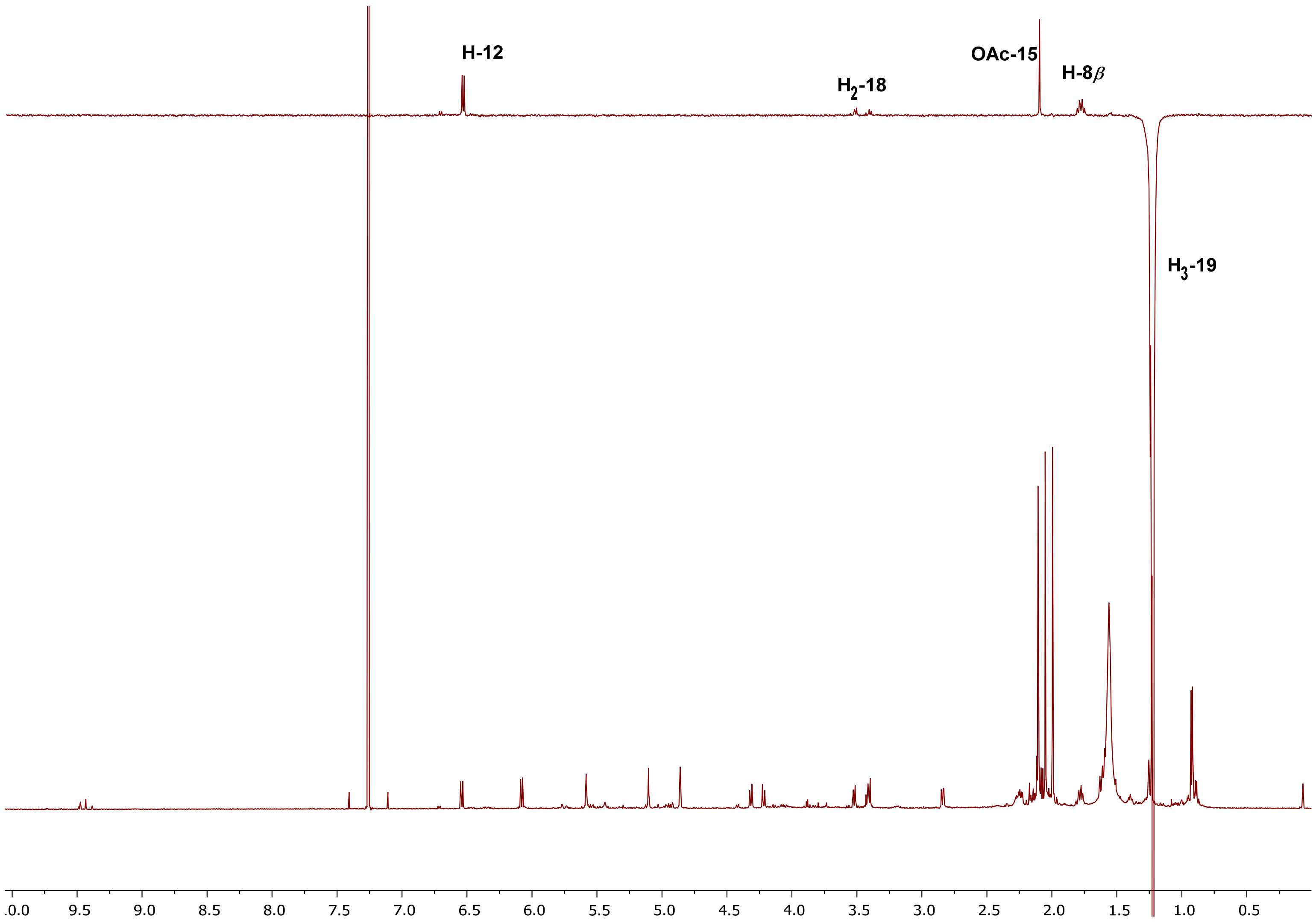

Figure S24d. 1D NOESY spectrum of compound **5**.

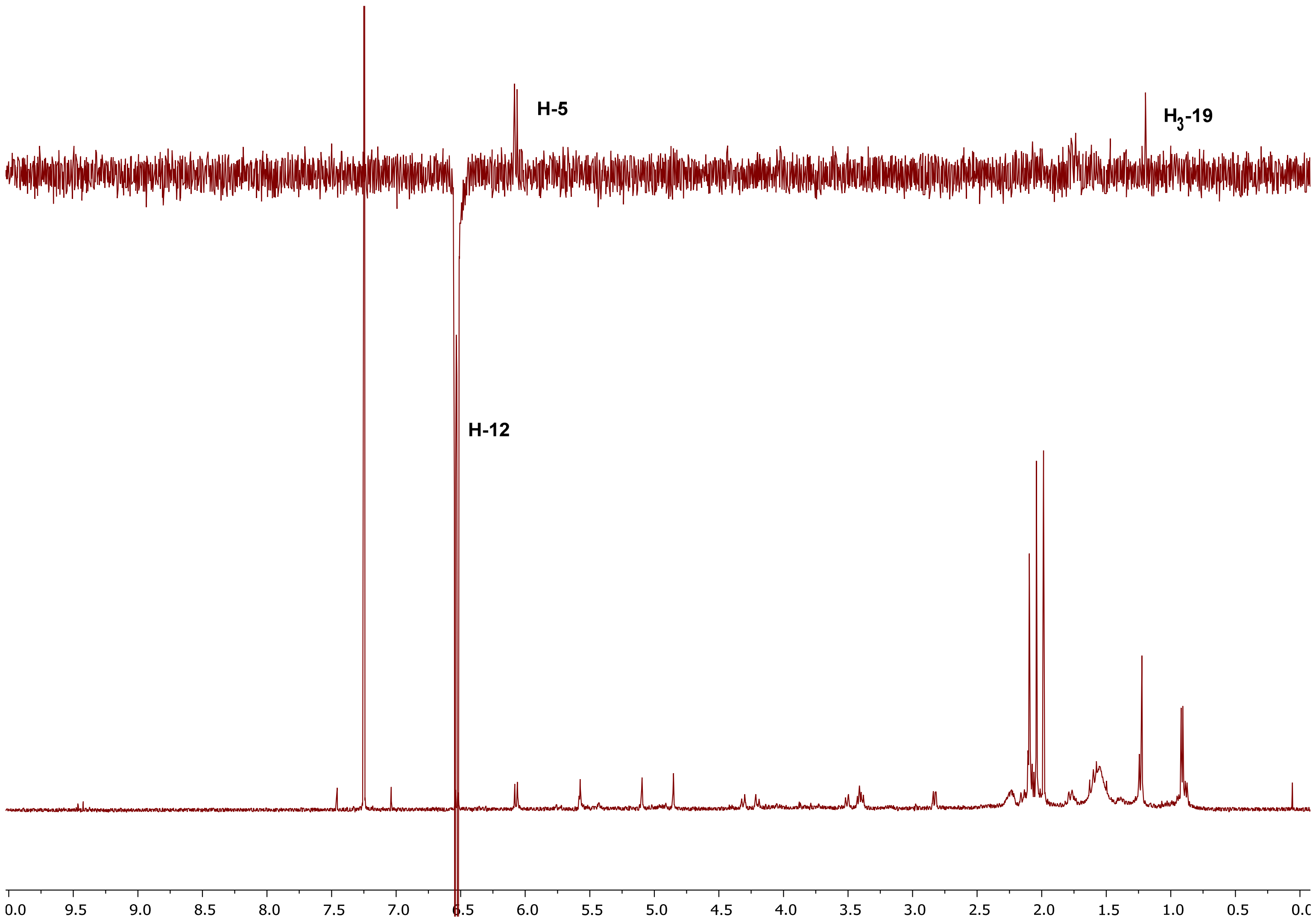

Figure S24e. 1D NOESY spectrum of compound **5**.

Elemental Composition Report

Single Mass Analysis

Tolerance = 5.0 mDa / DBE: min = -1.5, max = 50.0
Element prediction: Off
Number of isotope peaks used for i-FIT = 3

Monoisotopic Mass, Even Electron Ions
215 formula(e) evaluated with 3 results within limits (all results (up to 1000) for each mass)
Elements Used:
C: 0-50 H: 0-200 O: 0-20 Na: 0-1
C26H36O9 FELIPE
267 191 (0.749)

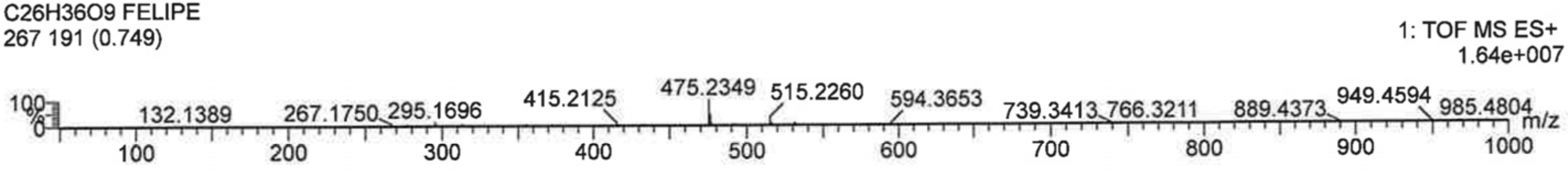

		mDa	PPM	DBE				
Minimum:				-1.5				
Maximum:		5.0	10.0	50.0				

Mass	Calc. Mass	mDa	PPM	DBE	i-FIT	Norm	Conf(%)	Formula
515.2260	515.2257	0.3	0.6	8.5	717.7	0.042	95.89	C26 H36 O9 Na
	515.2281	-2.1	-4.1	11.5	720.9	3.197	4.09	C28 H35 O9
	515.2222	3.8	7.4	20.5	725.9	8.264	0.03	C35 H31 O4

Figure S25. HRMS of compound **5**.

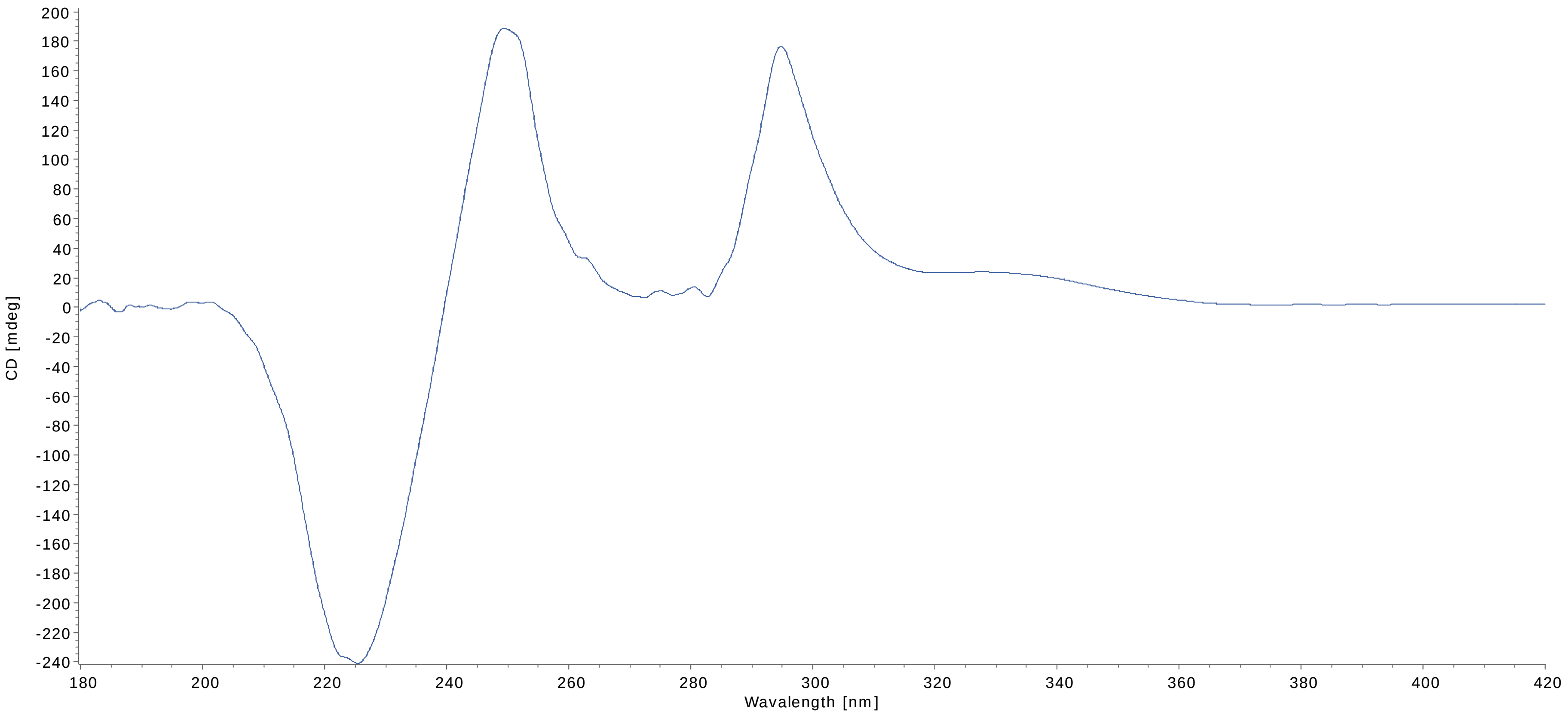

Figure S26. ECD of compound **5**.

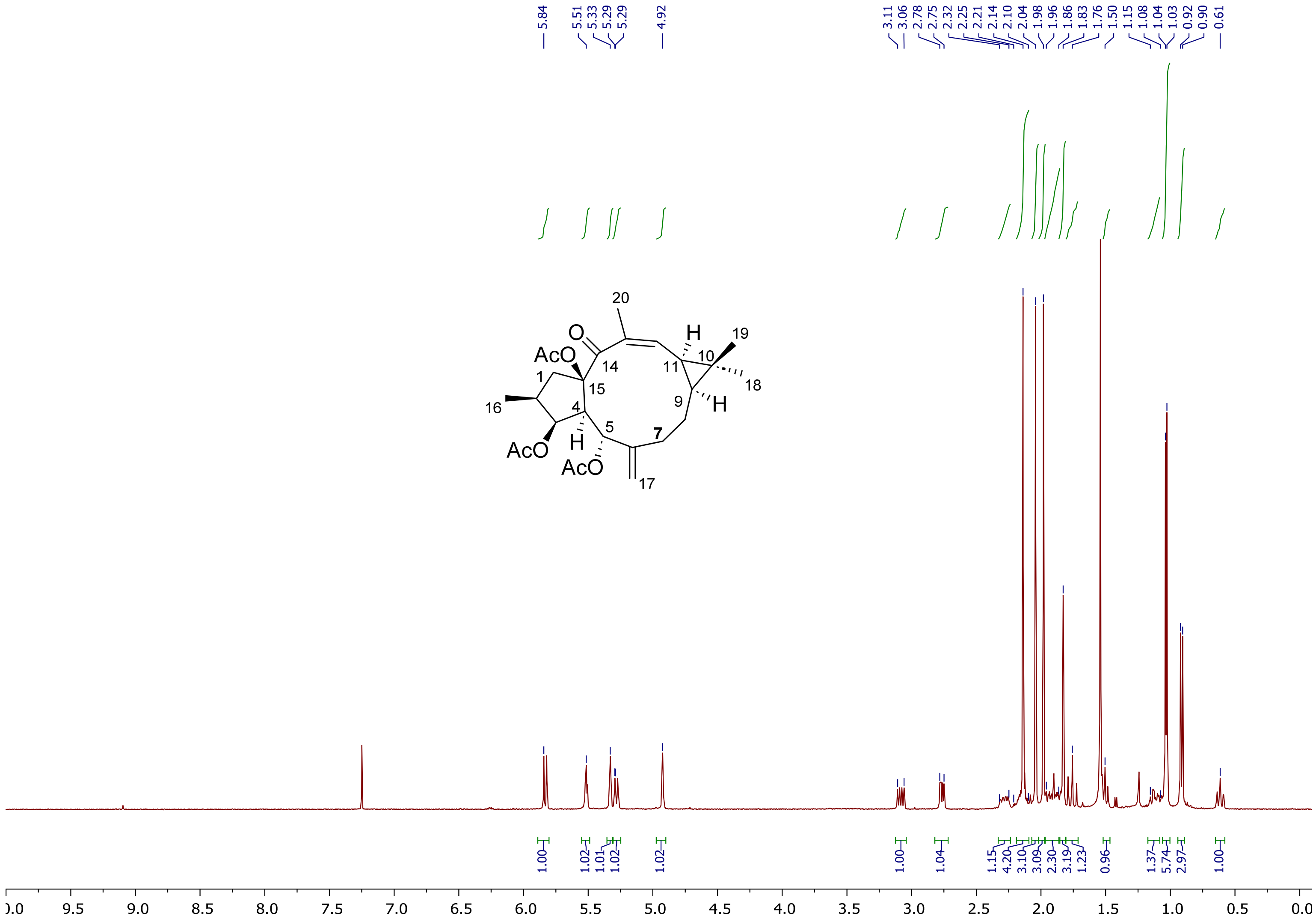

Figure S27. ^{1}H NMR spectrum (400 MHz) of compound **6** in $CDCl_3$.

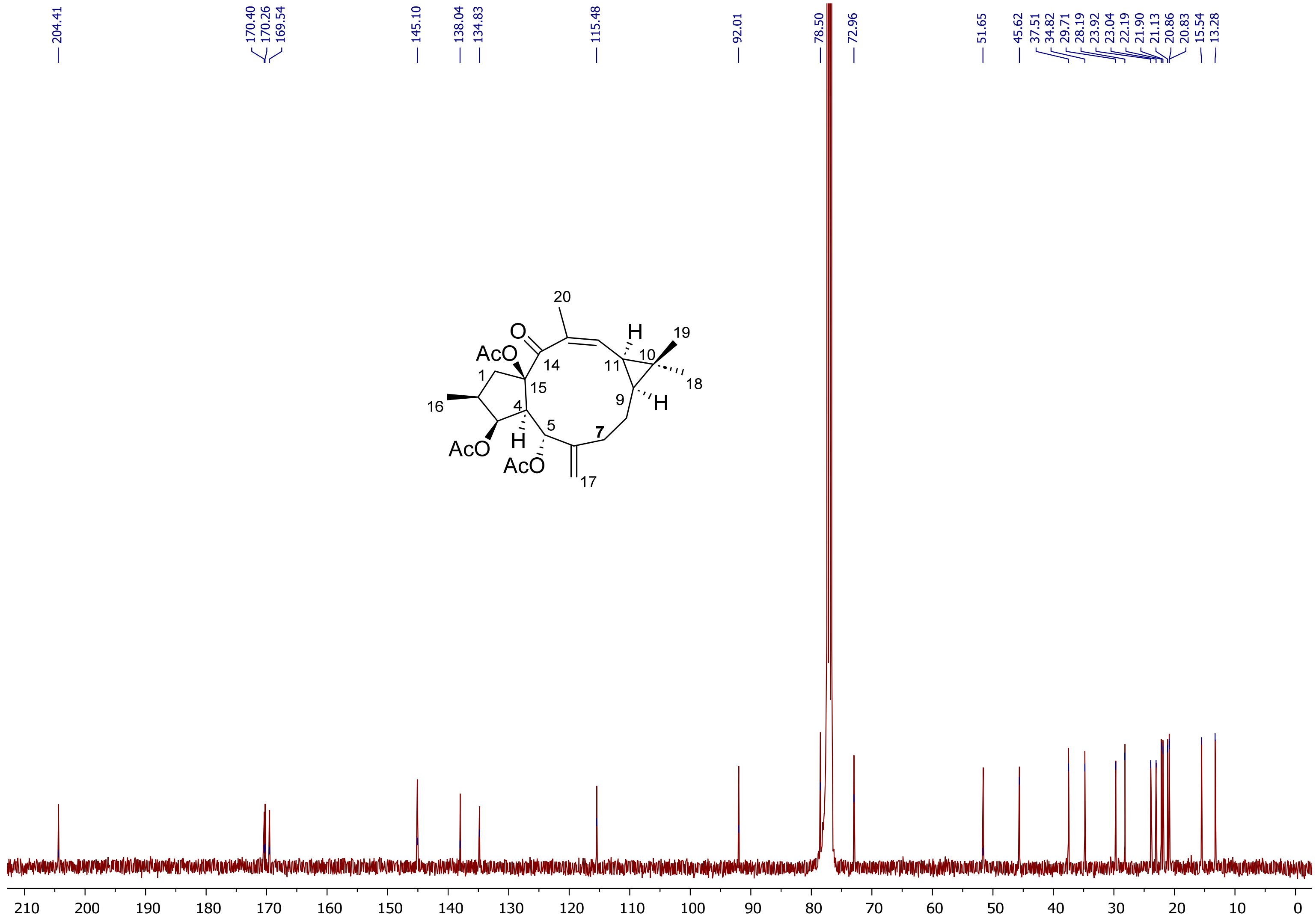

Figure S28. ^{13}C NMR spectrum (100 Mz) of compound **6** in $CDCl_3$.

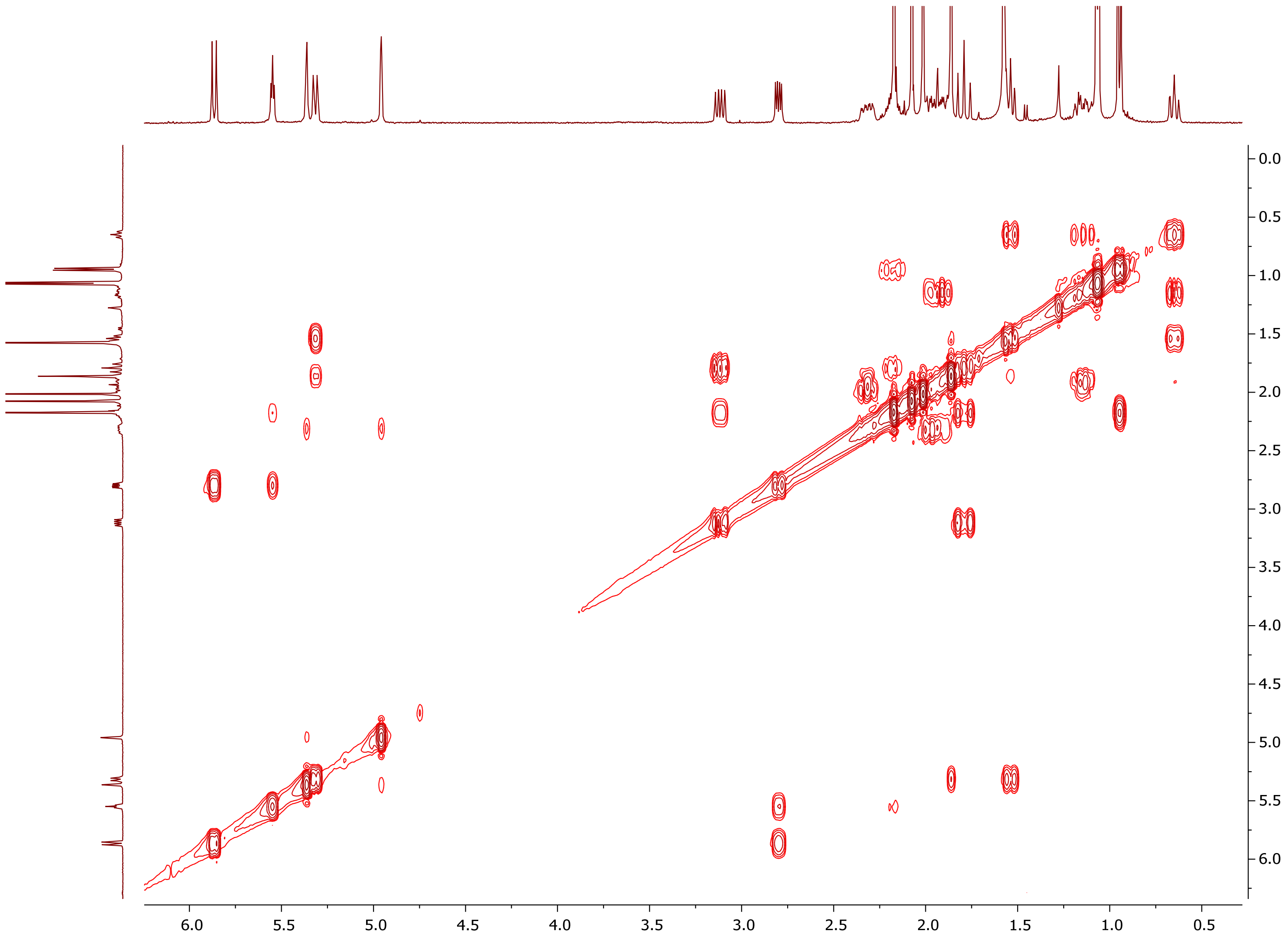

Figure S29. gCOSY spectrum of compound **6**.

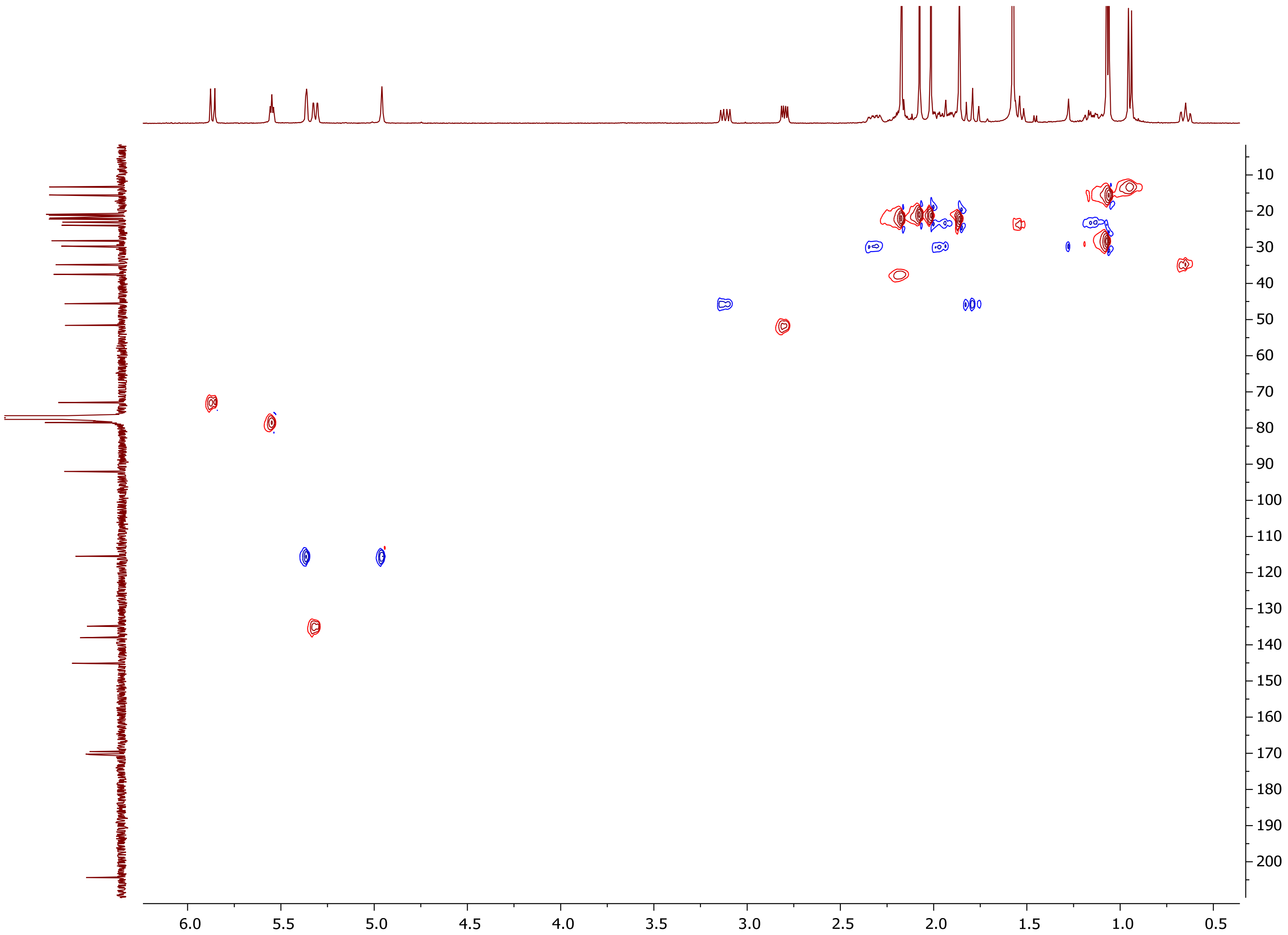

Figure S30. gHSQC spectrum of compound **6**.

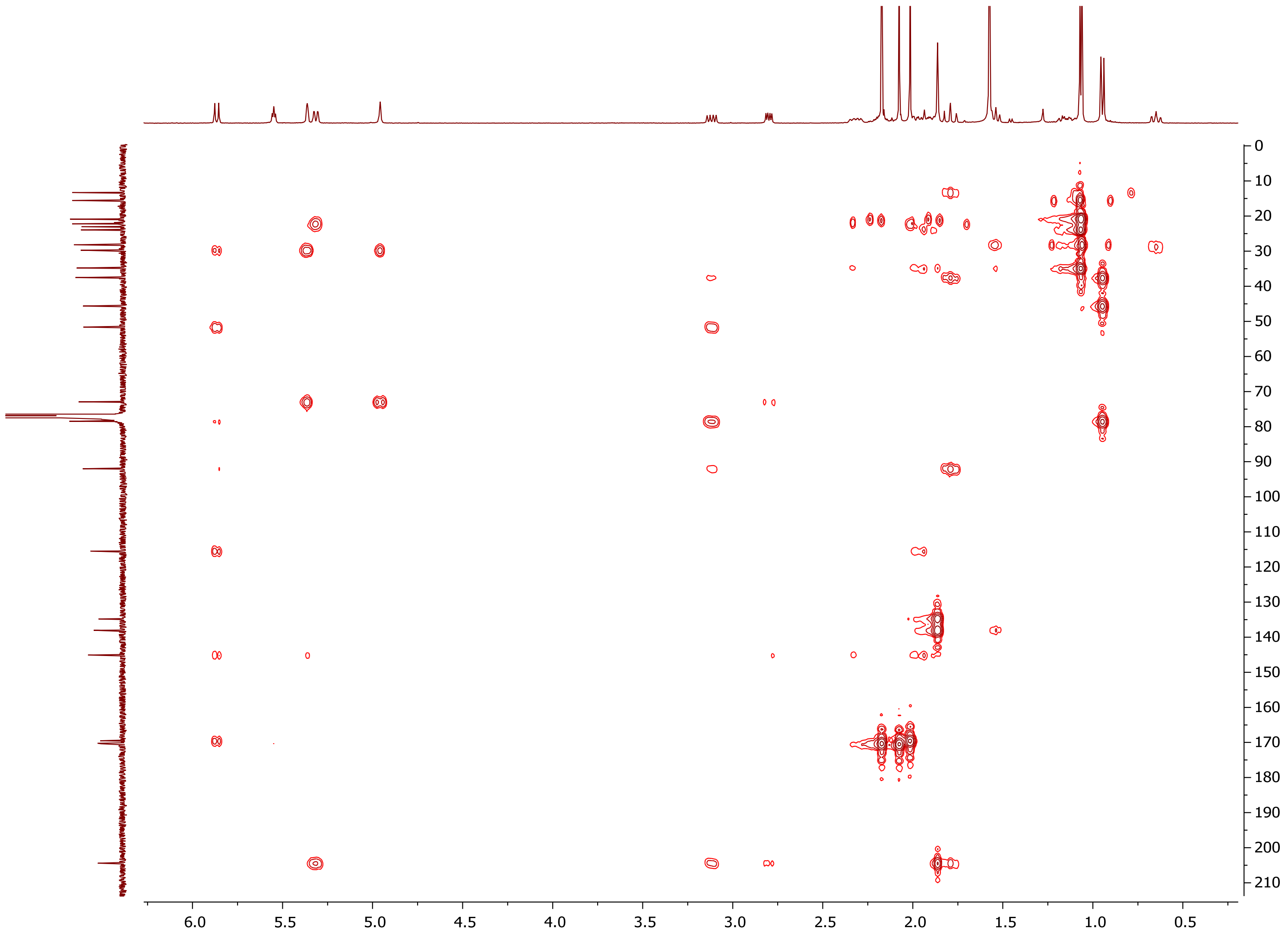

Figure S31. gHMBC spectrum of compound **6**.

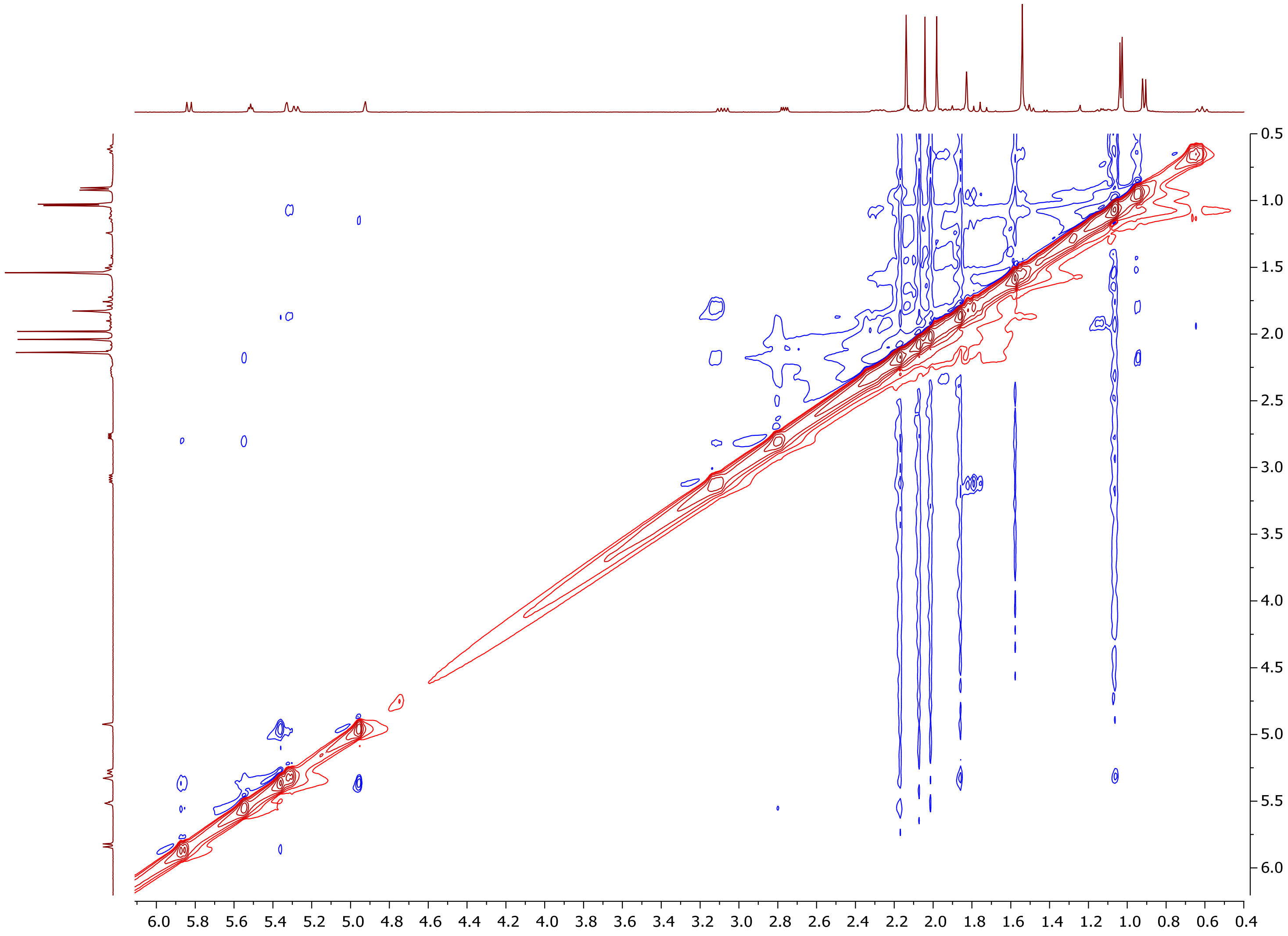

Figure S32. 2D NOESY spectrum of compound **6**.

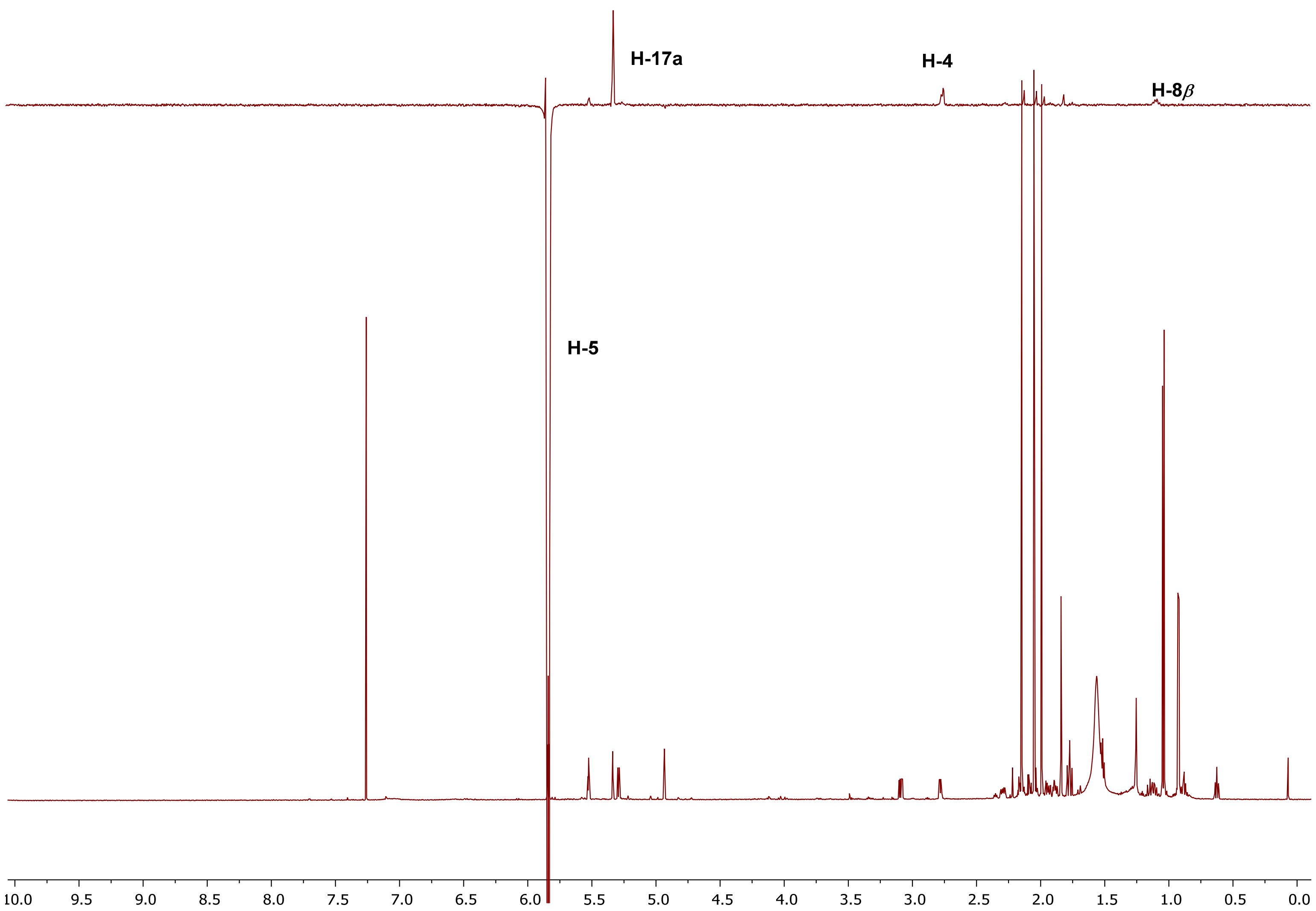

Figure S33a. 1D NOESY spectrum of compound **6**.

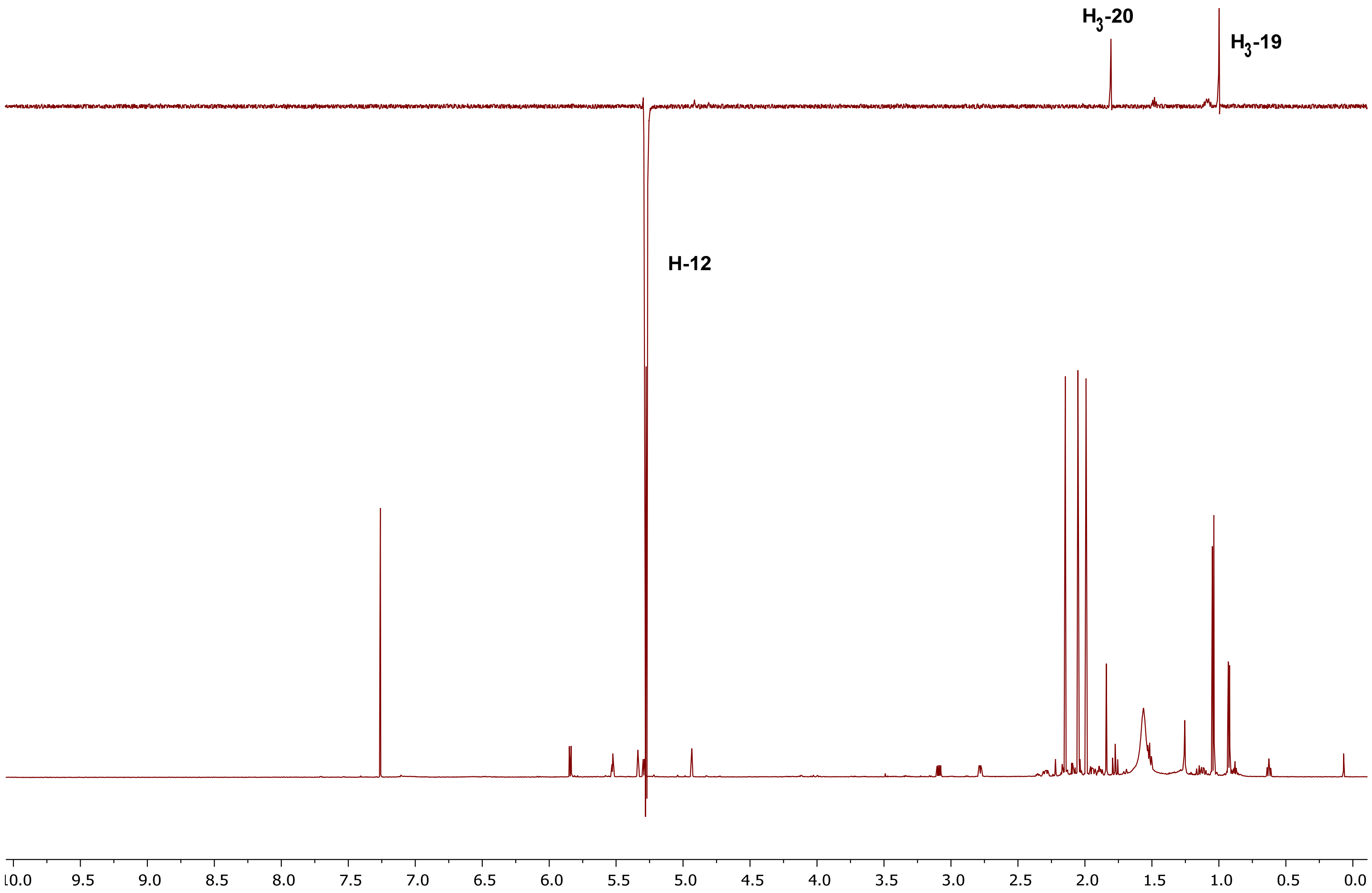

Figure S33b. 1D NOESY spectrum of compound **6**.

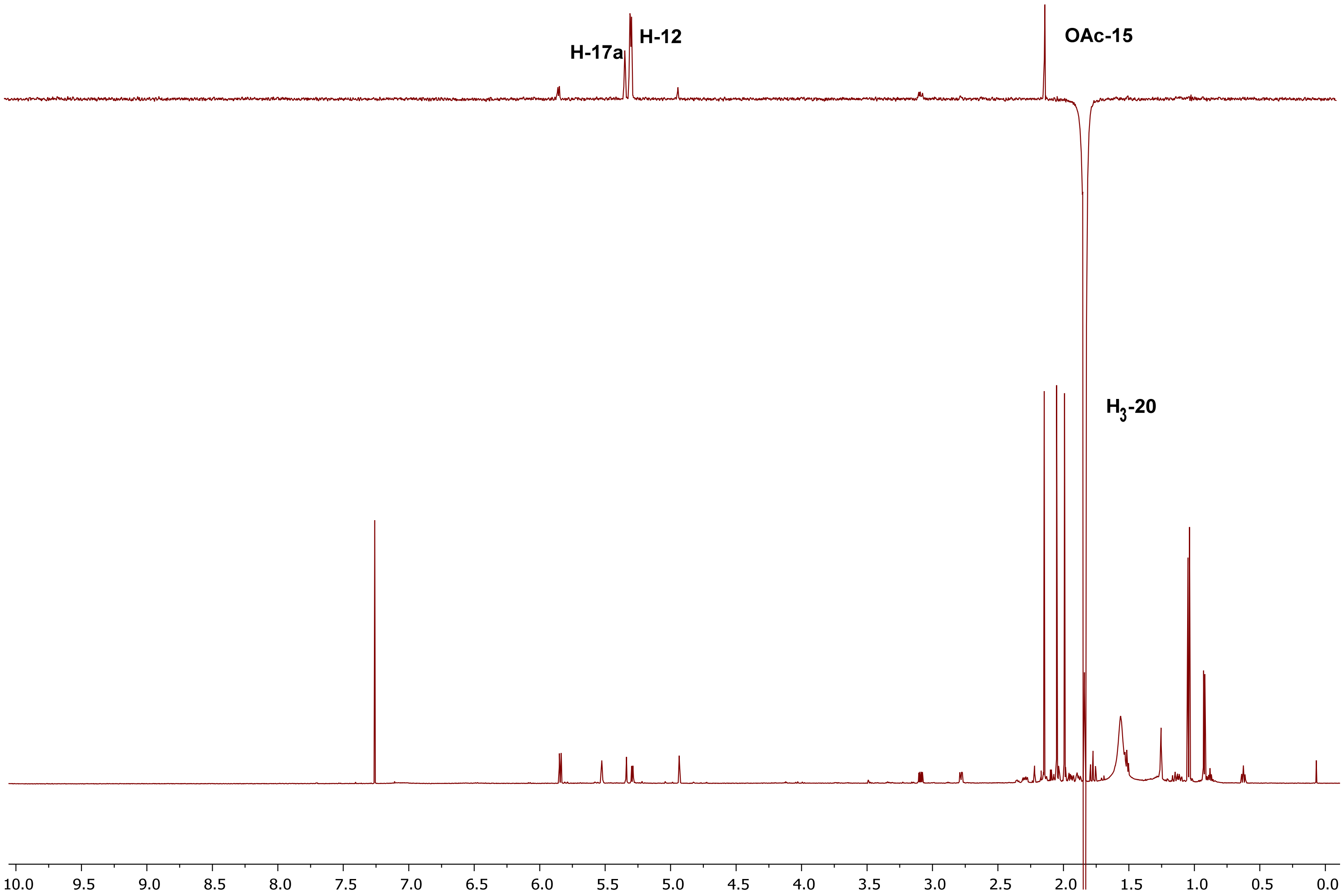

Figure S33c. 1D NOESY spectrum of compound **6**.

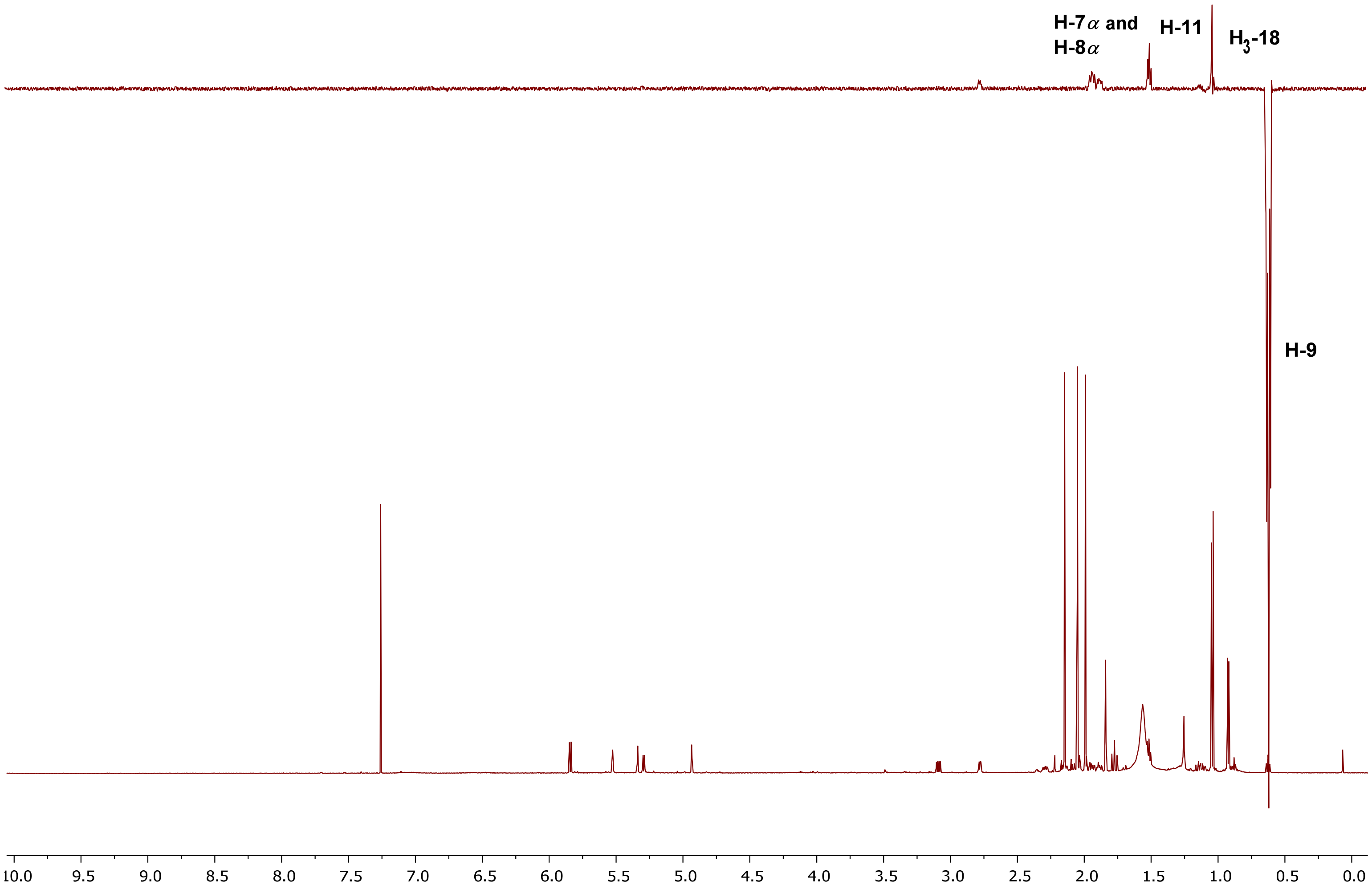

Figure S33d. 1D NOESY spectrum of compound **6**.

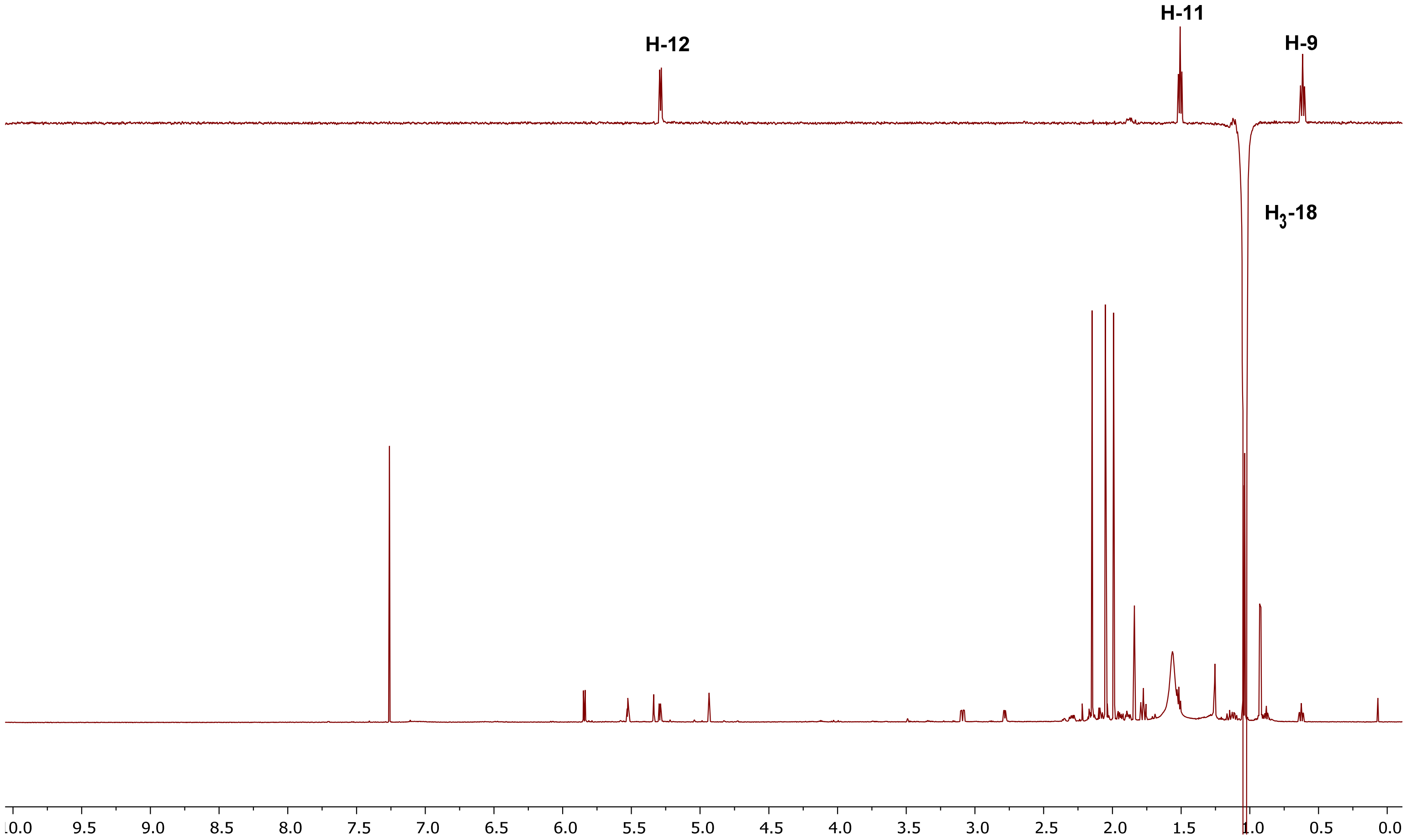

Figure S33e. 1D NOESY spectrum of compound **6**.

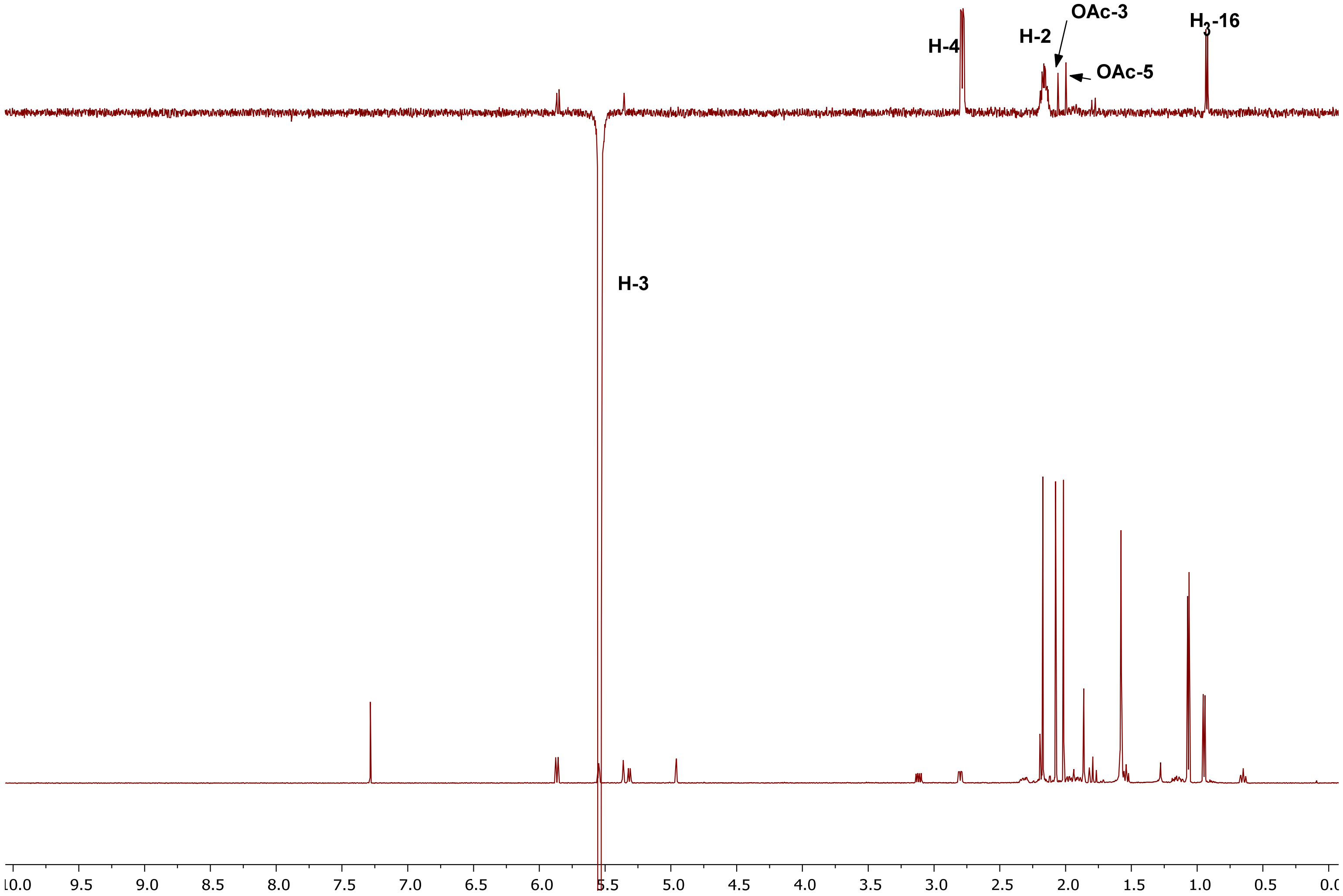

Figure S33f. 1D NOESY spectrum of compound **6**.

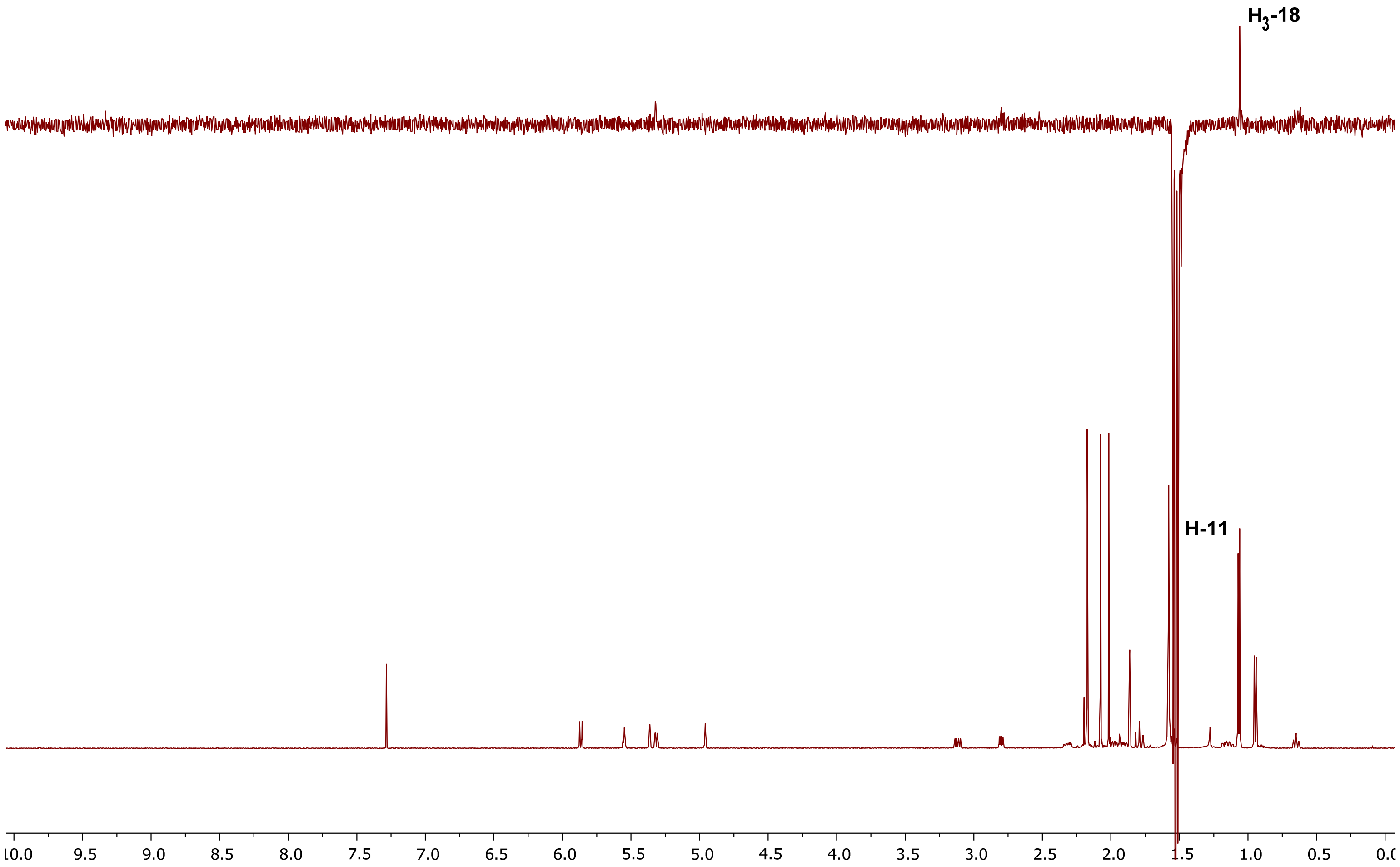

Figure S33g. 1D NOESY spectrum of compound **6**.

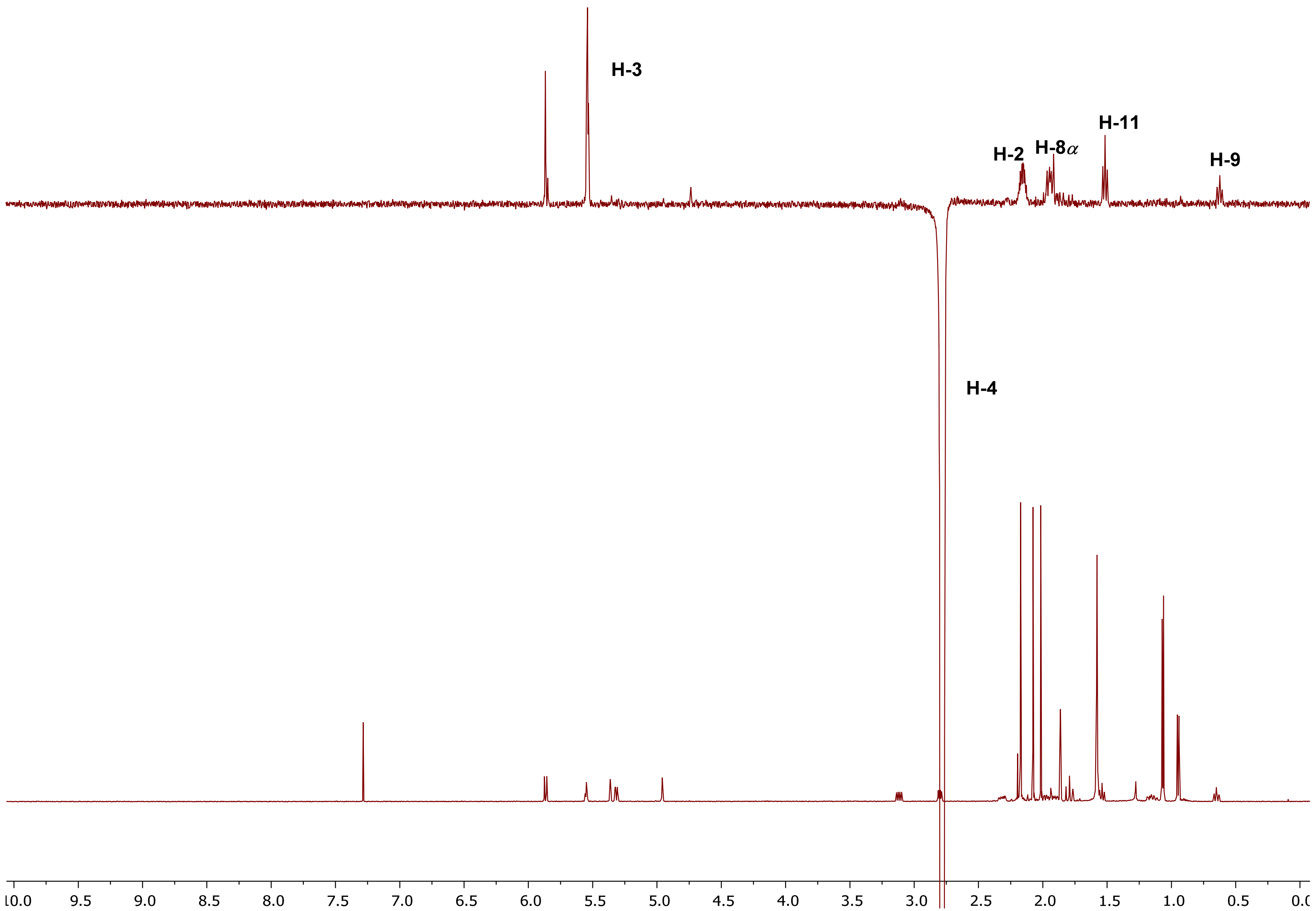

Figure S33h. 1D NOESY spectrum of compound **6**.

Elemental Composition Report

Single Mass Analysis

Tolerance = 5.0 mDa / DBE: min = -1.5, max = 80.0
Element prediction: Off
Number of isotope peaks used for i-FIT = 5

Monoisotopic Mass, Even Electron Ions
93 formula(e) evaluated with 3 results within limits (all results (up to 1000) for each mass)
Elements Used:
C: 0-30 H: 0-50 O: 0-15 23Na: 0-1

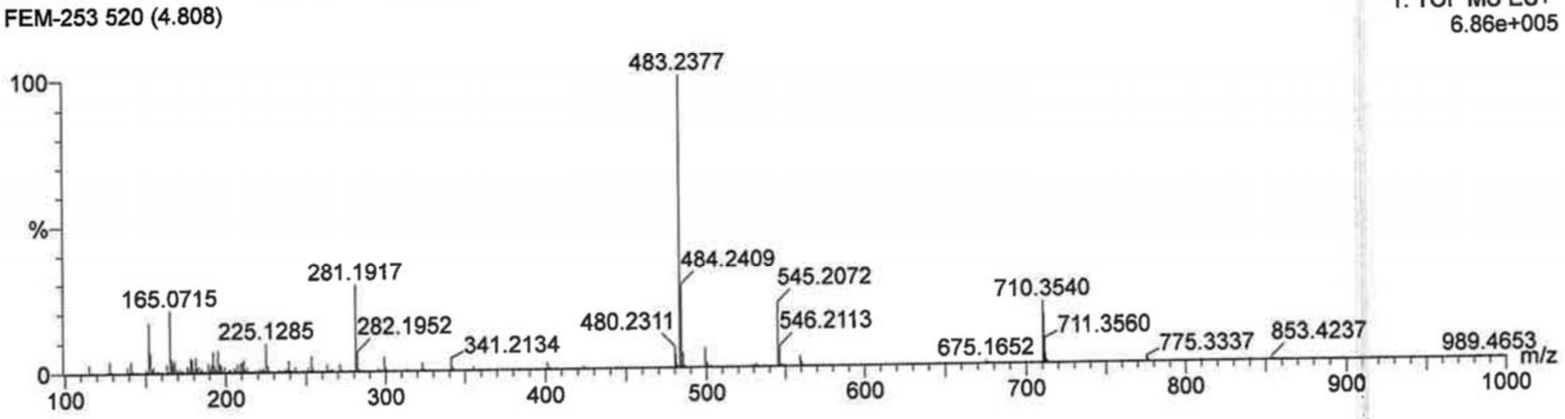

Minimum:				-1.5				
Maximum:		5.0	10.0	80.0				
Mass	Calc. Mass	mDa	PPM	DBE	i-FIT	Norm	Conf(%)	Formula
483.2377	483.2359	1.8	3.7	8.5	1058.8	0.001	99.86	C26 H36 O7 23Na
	483.2383	-0.6	-1.2	11.5	1065.4	6.598	0.14	C28 H35 O7
	483.2417	-4.0	-8.3	-0.5	1068.7	9.859	0.01	C19 H40 O12 23Na

Figure S34. HRMS of compound **6**.

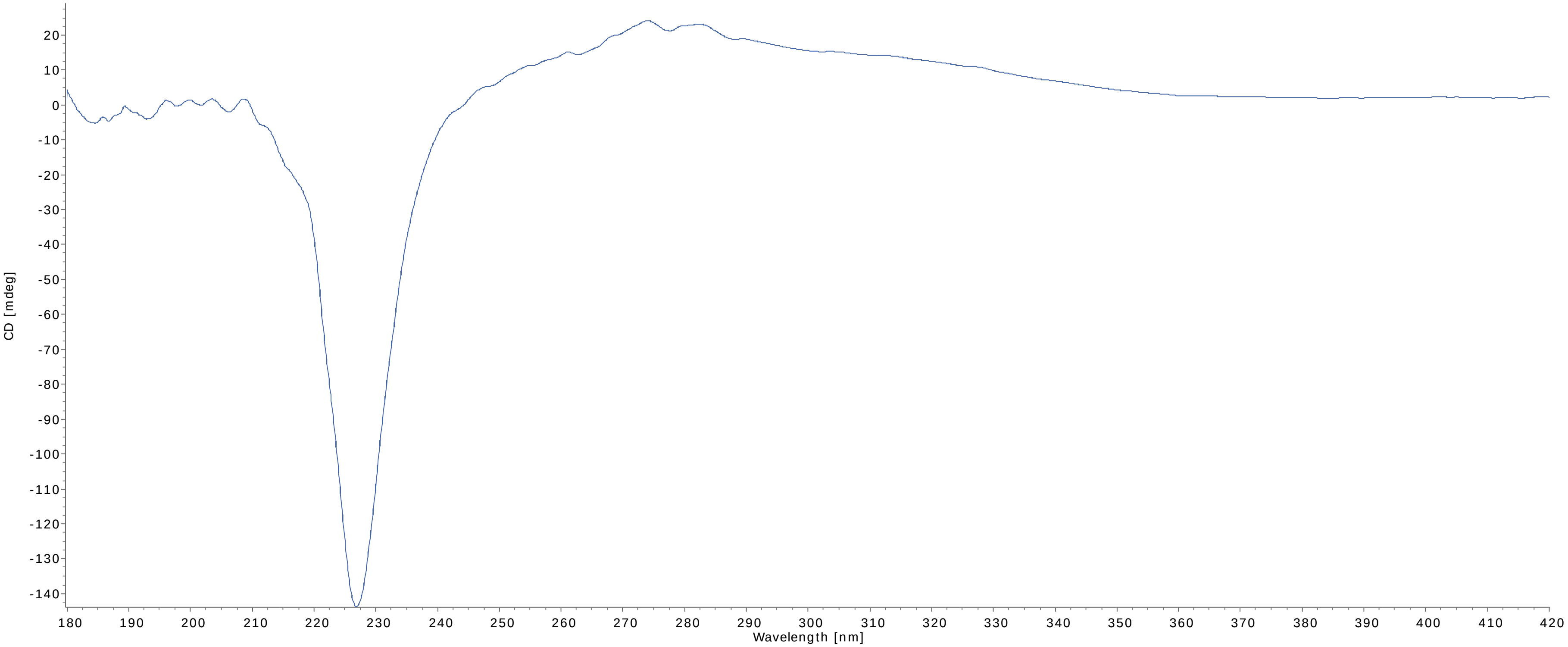

Figure S35. ECD of compound **6**.

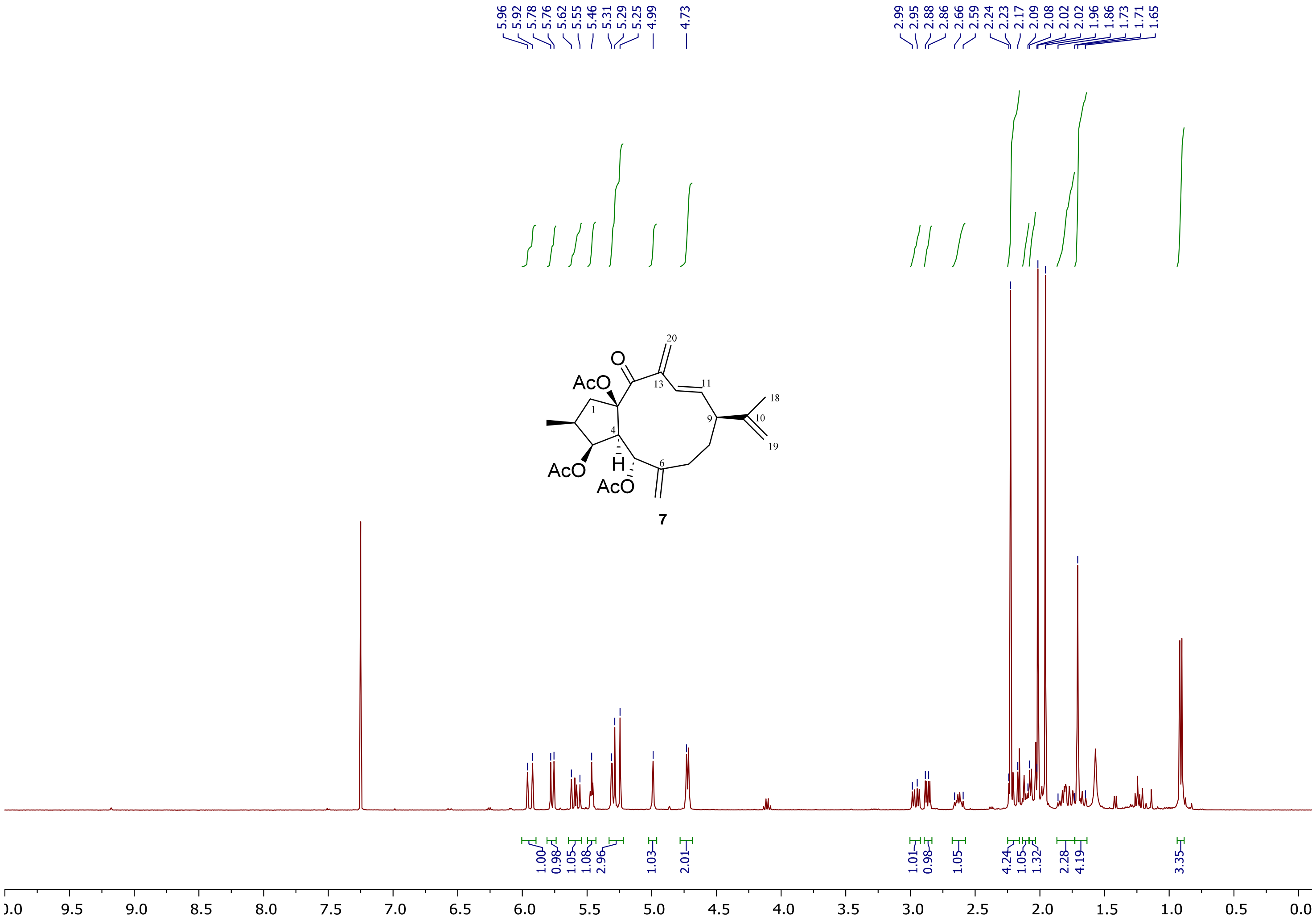

Figure S36. ^{1}H NMR spectrum (400 MHz) of compound **7** in $CDCl_3$.

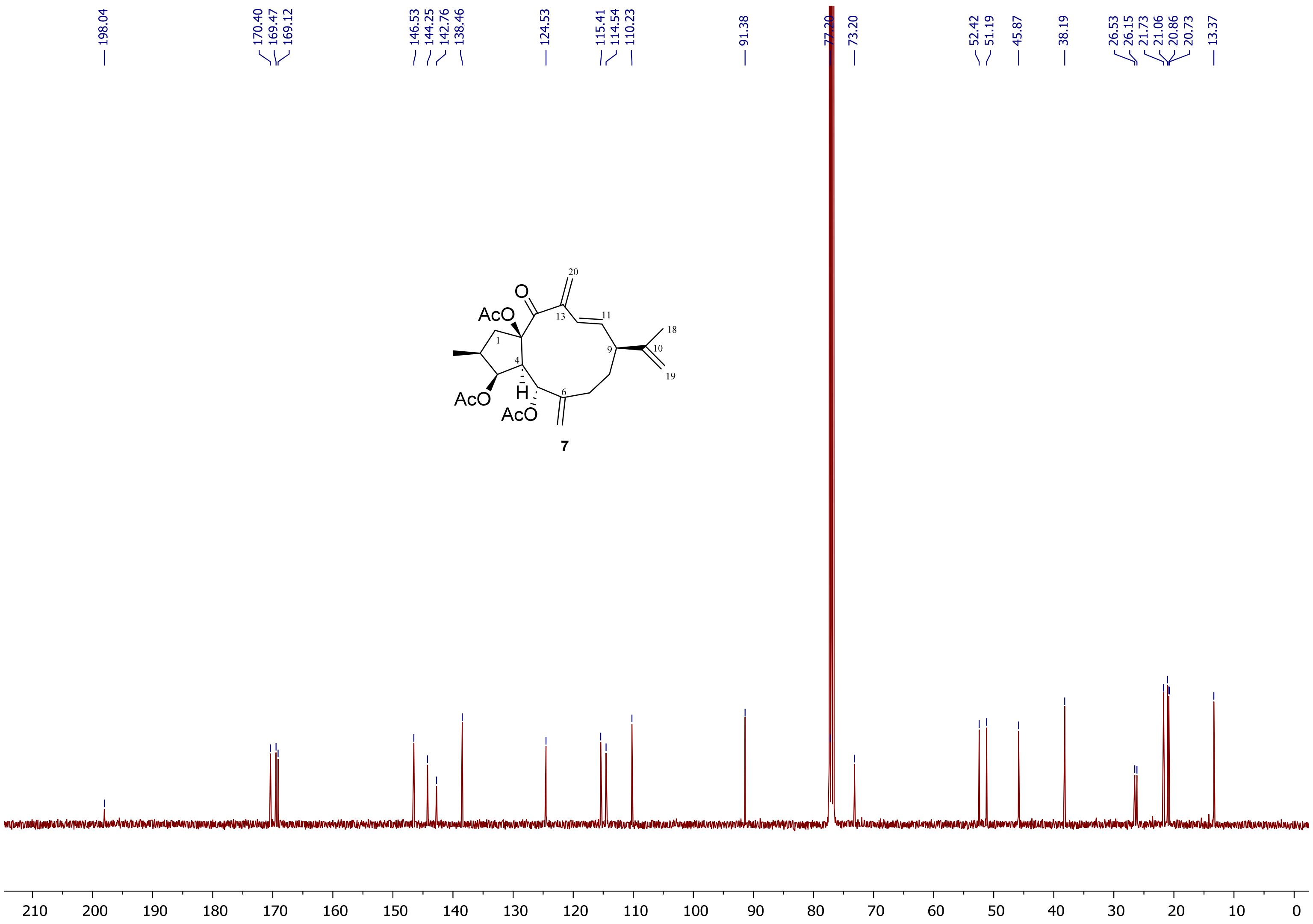

Figure S37. ^{13}C NMR spectrum (100 MHz) of compound **7** in $CDCl_3$.

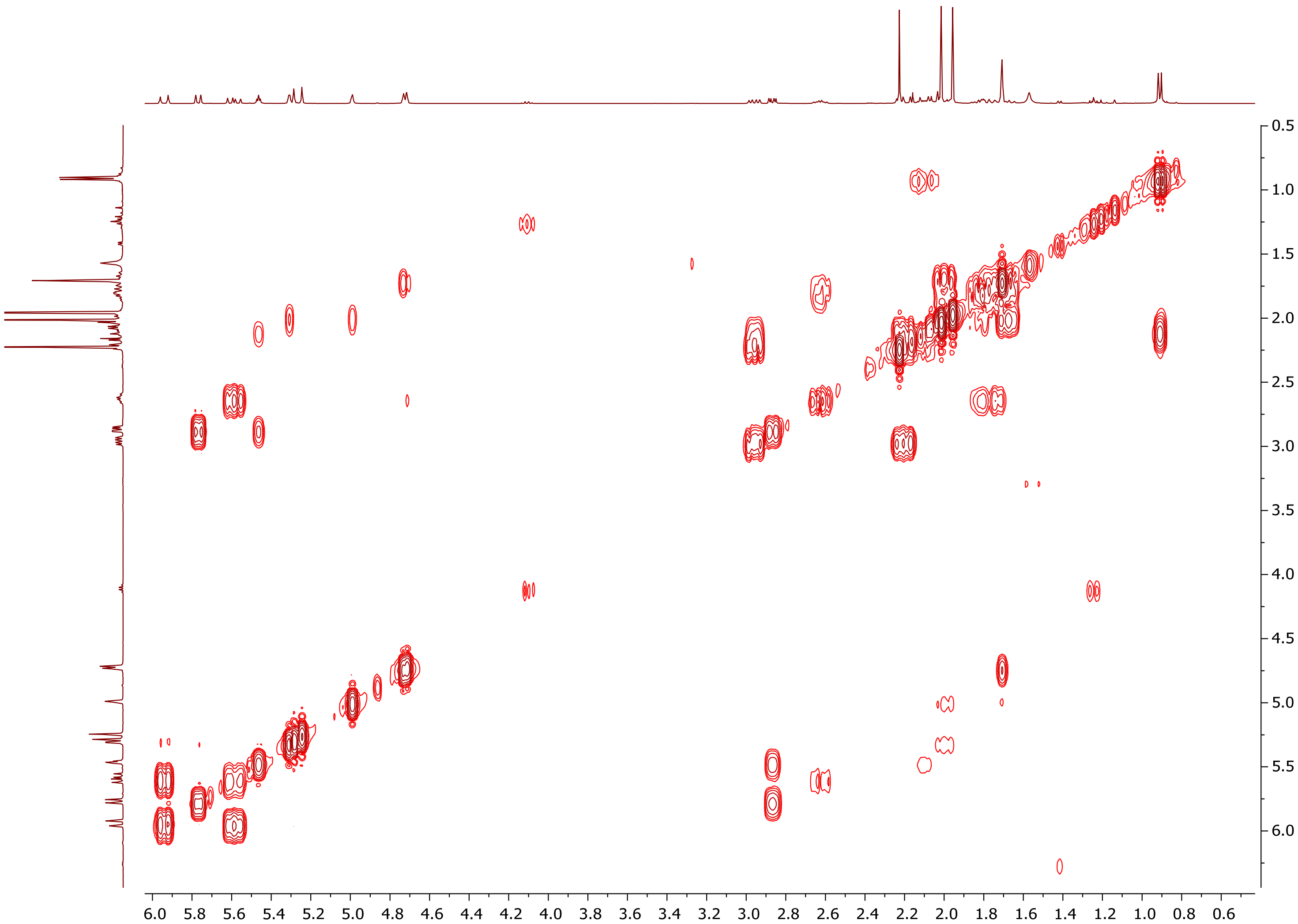

Figure S38. gCOSY spectrum of compound **7**.

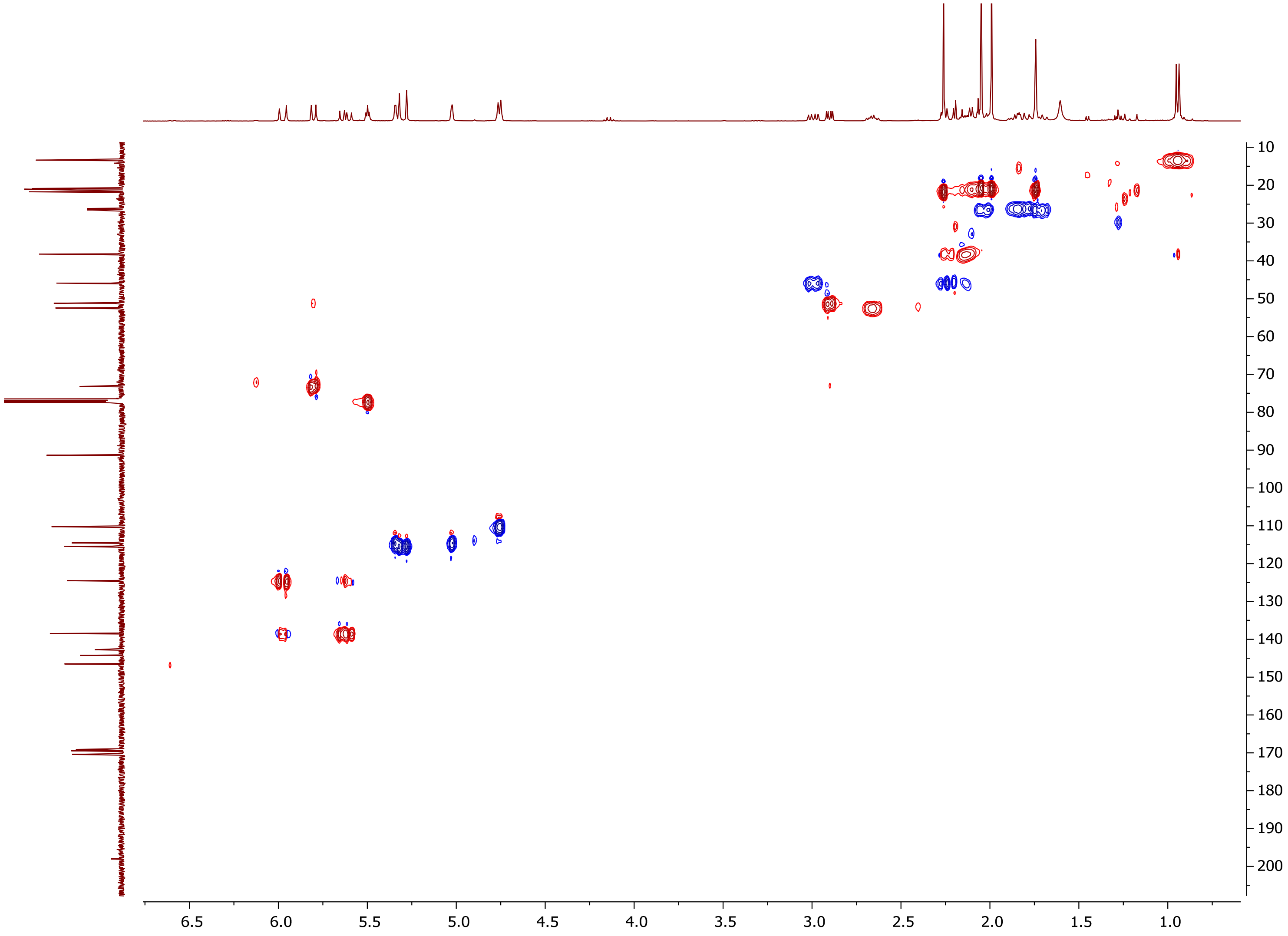

Figure S39. gHSQC spectrum of compound **7**.

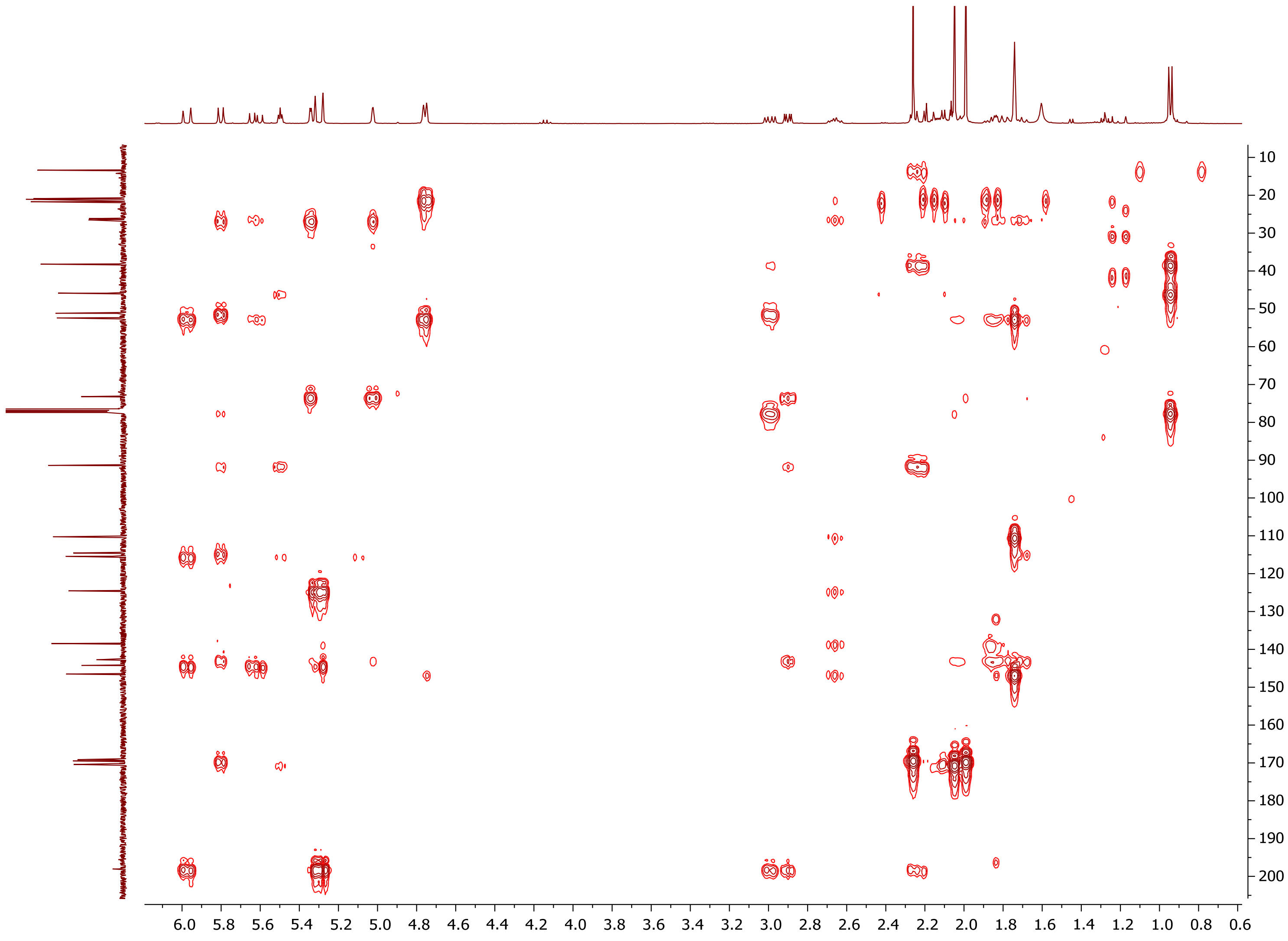

Figure S40. gHMBC spectrum of compound **7**.

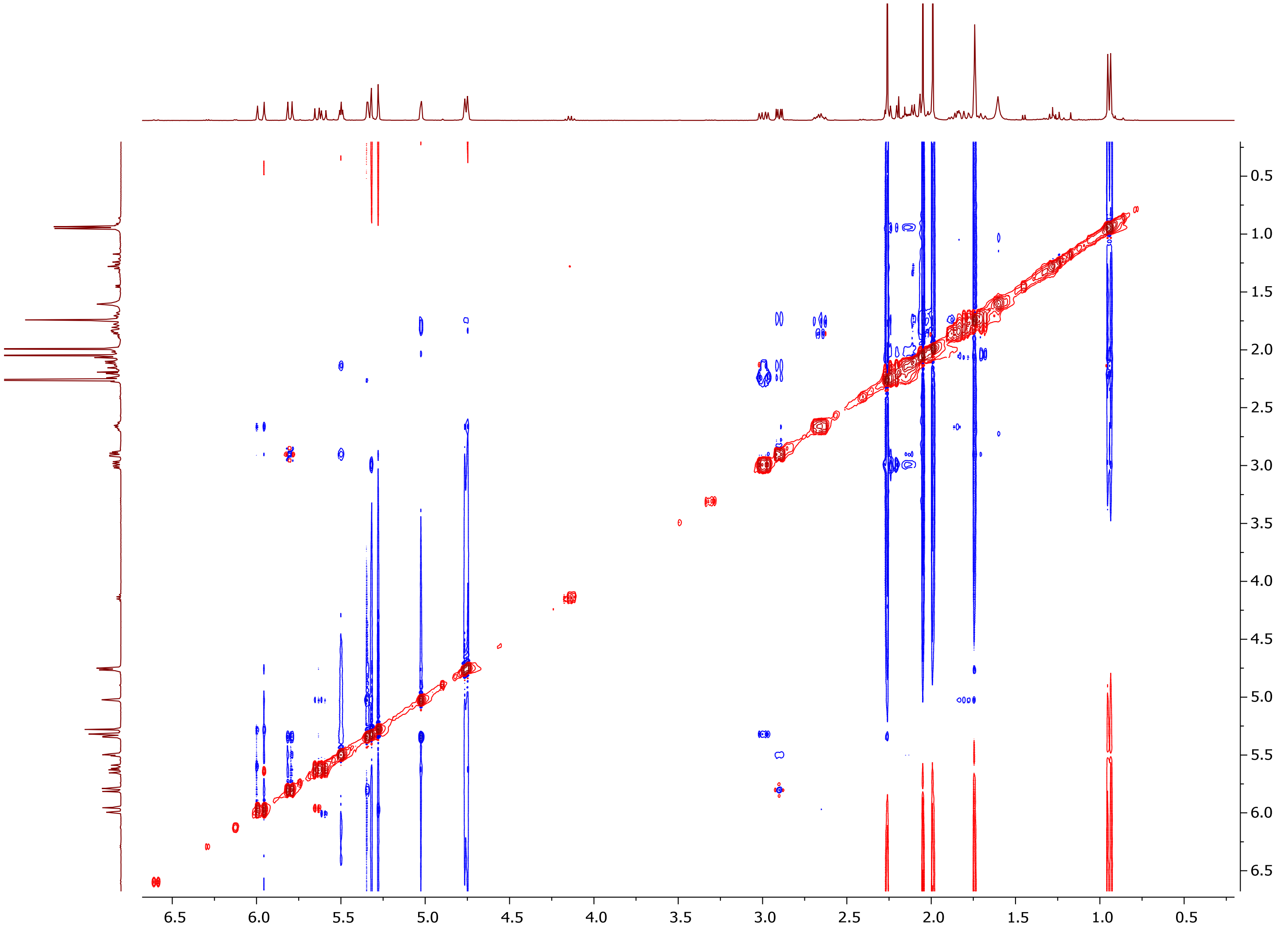

Figure S41. 2D NOESY spectrum of compound **7**.

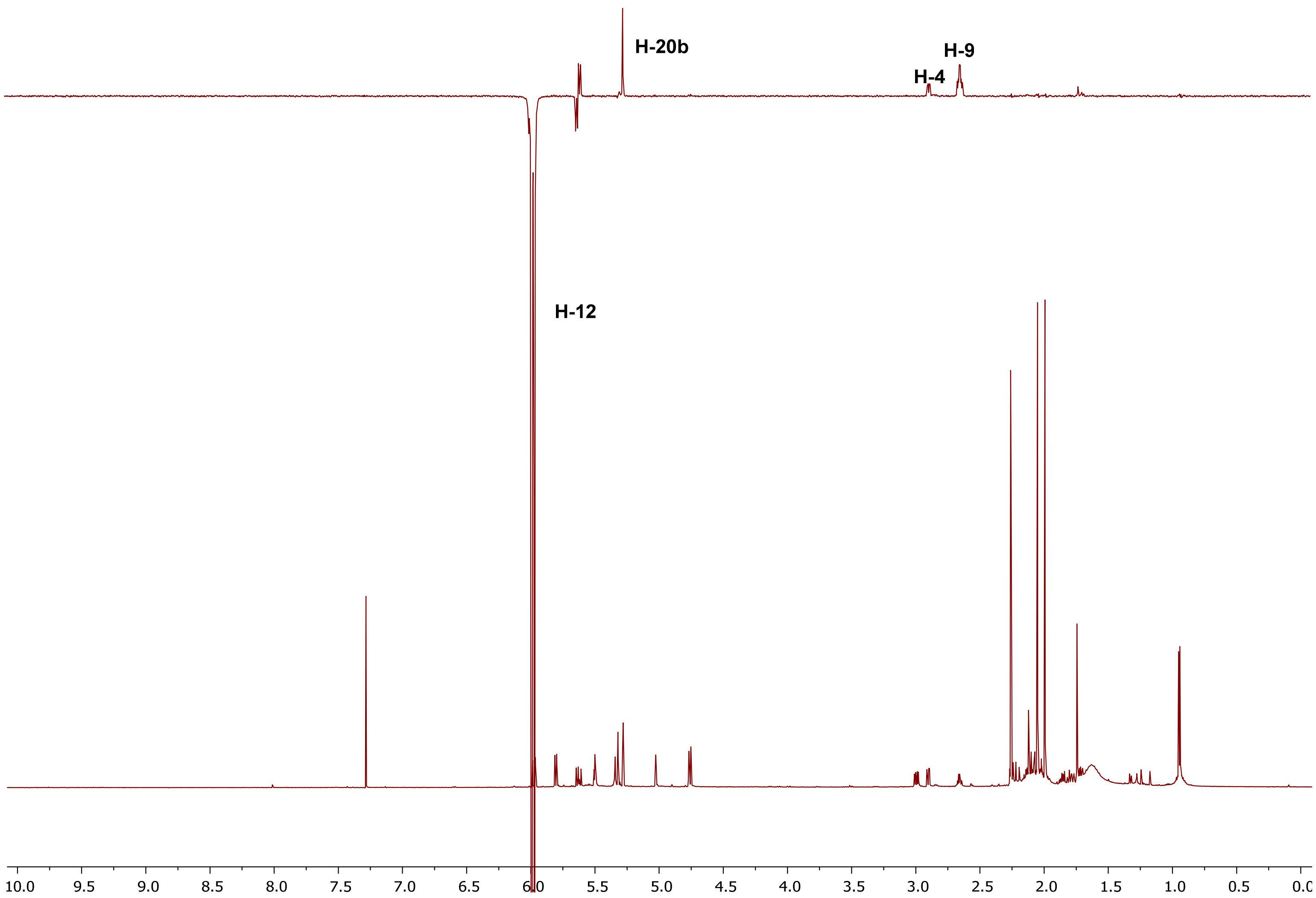

Figure S42a. 1D NOESY spectrum of compound **7**.

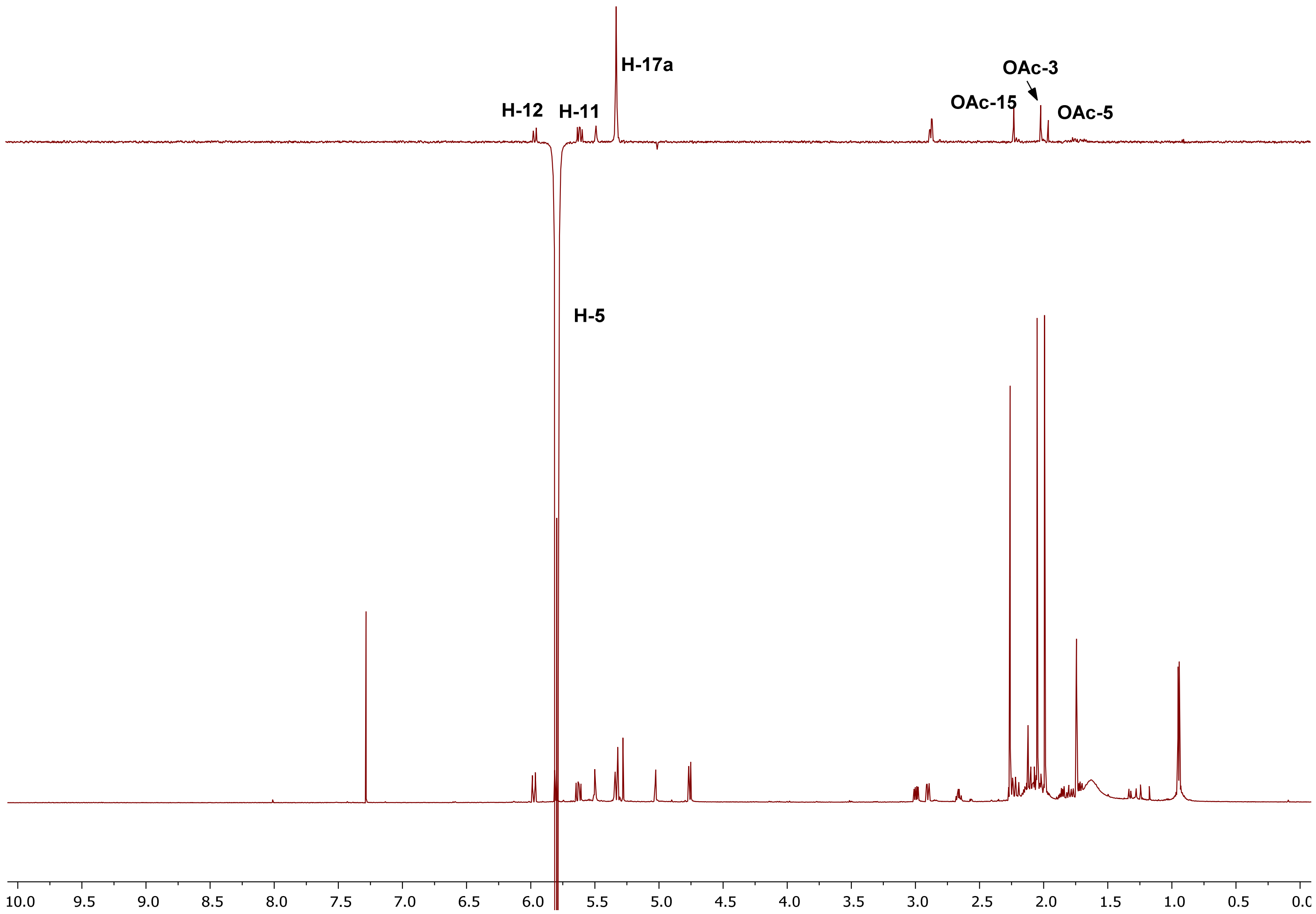

Figure S42b. 1D NOESY spectrum of compound **7**.

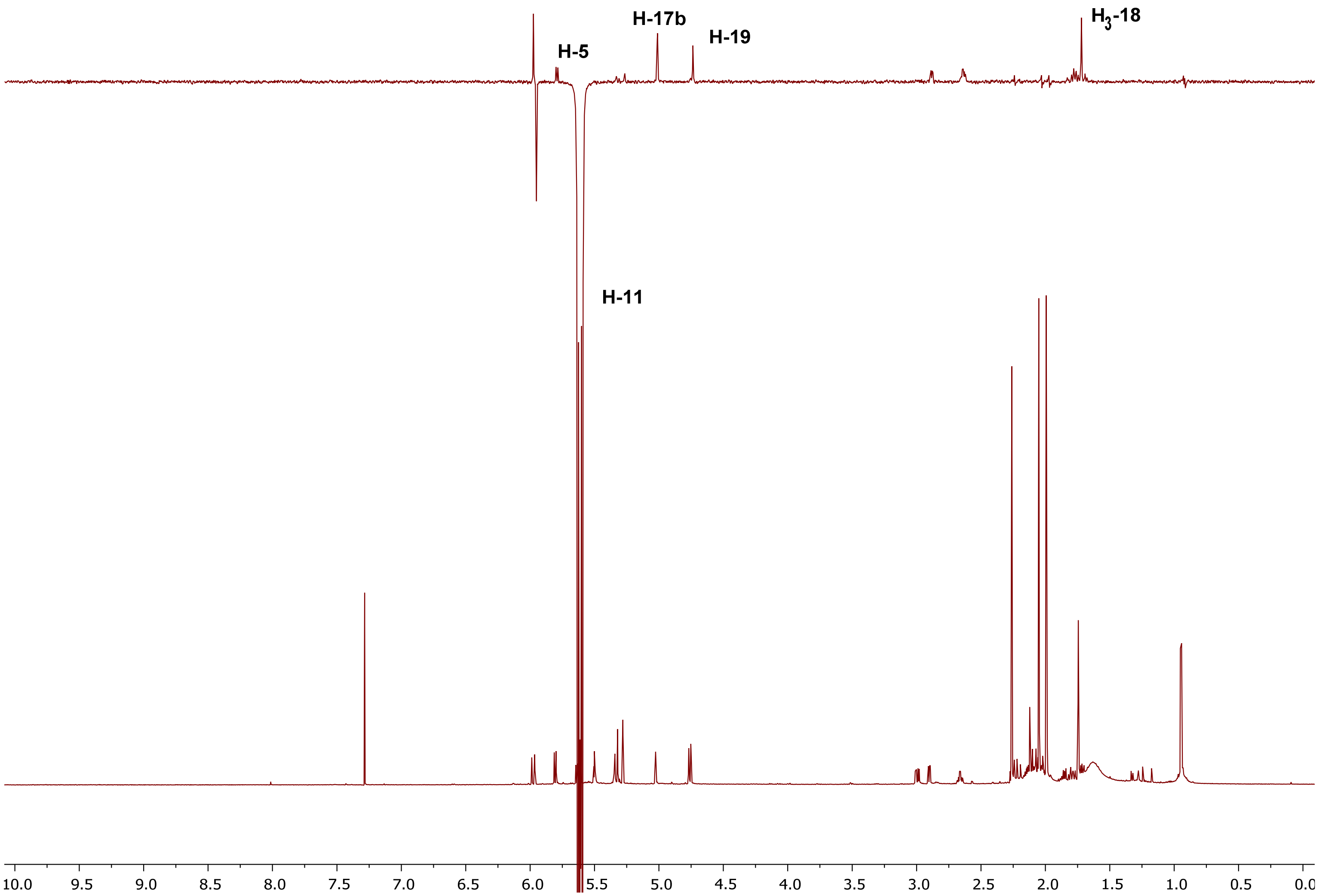

Figure S42c. 1D NOESY spectrum of compound **7**.

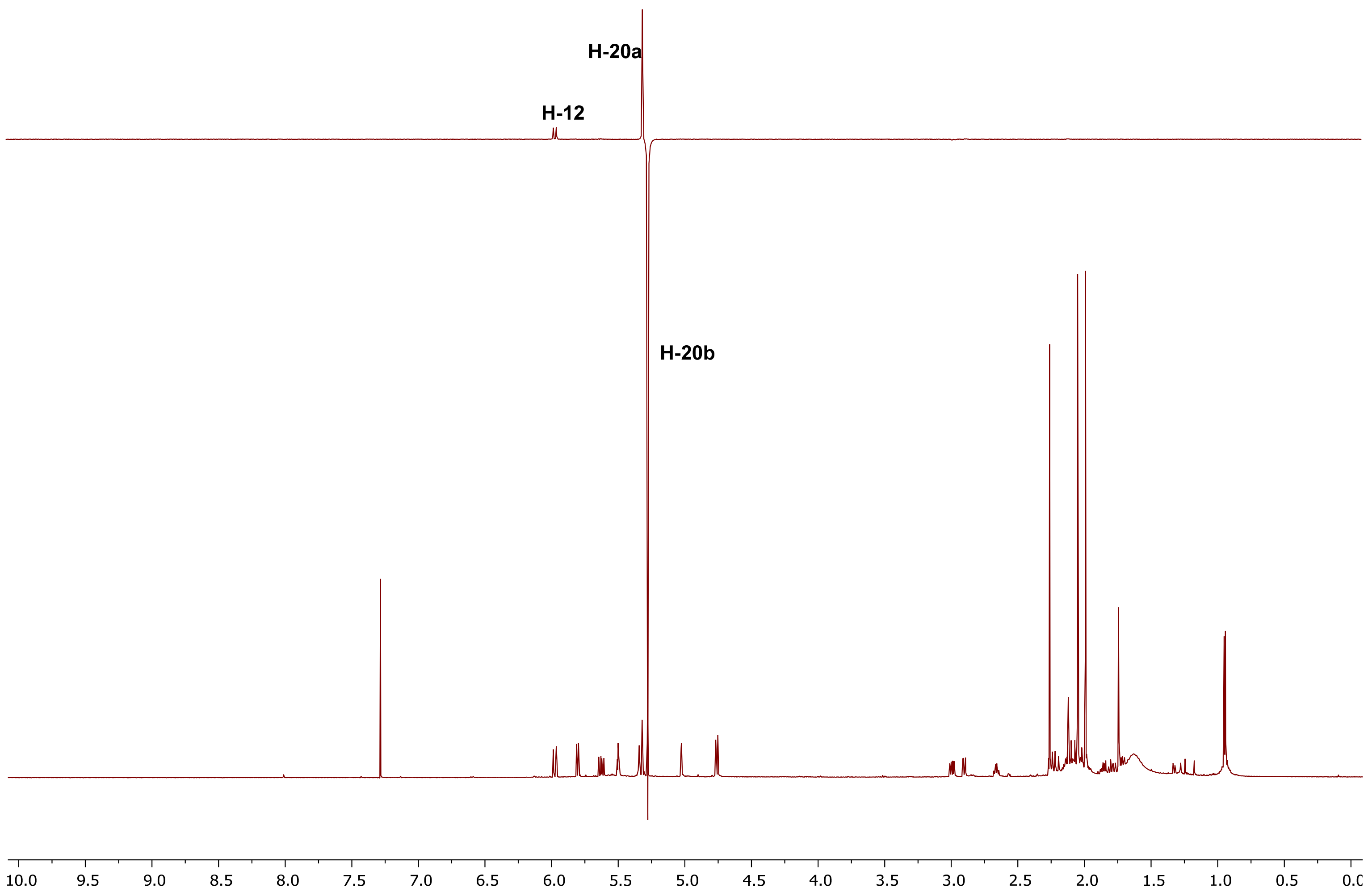

Figure S42d. 1D NOESY spectrum of compound **7**.

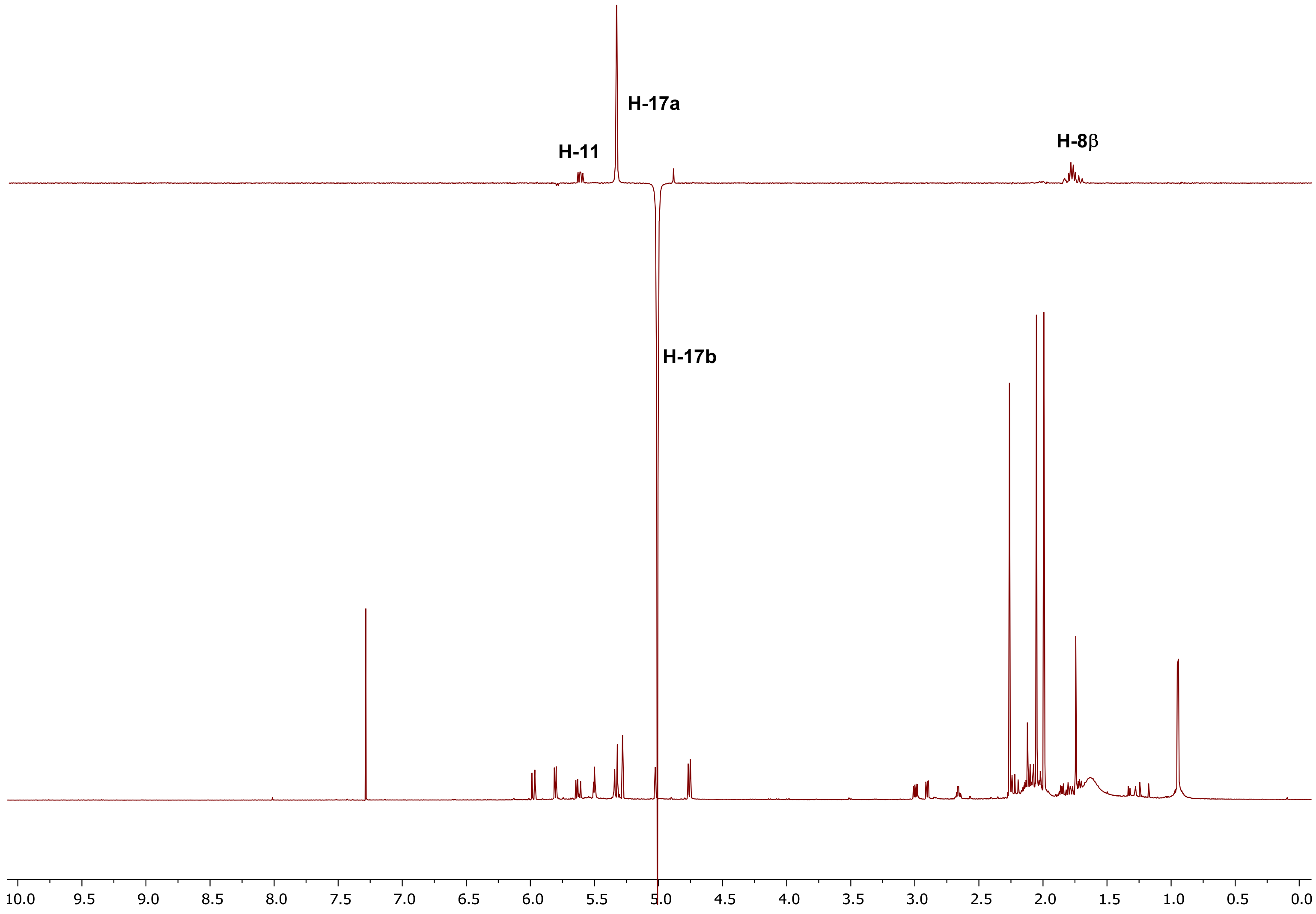

Figure S42e. 1D NOESY spectrum of compound **7**.

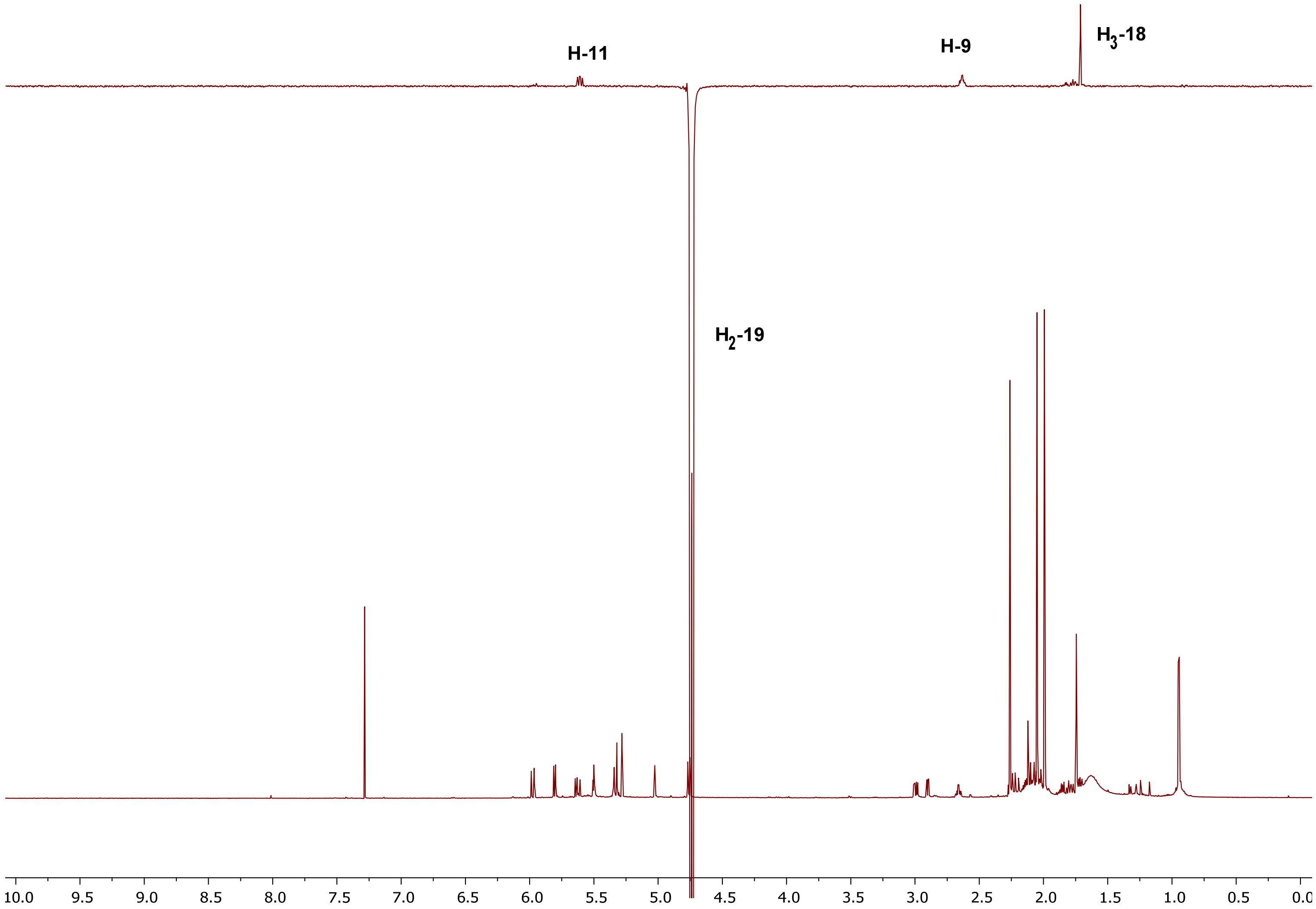

Figure S42f. 1D NOESY spectrum of compound **7**.

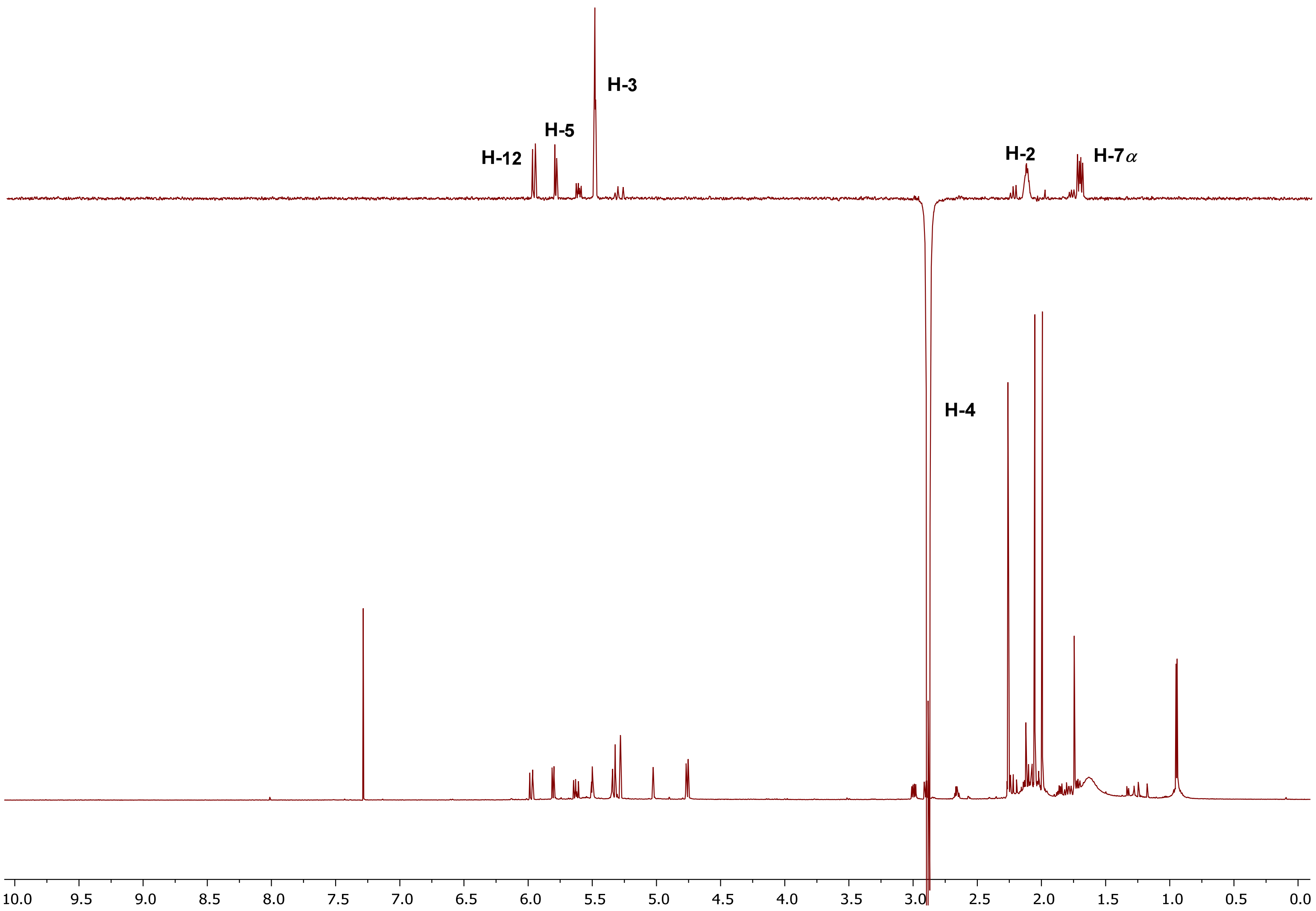

Figure S42g. 1D NOESY spectrum of compound **7**.

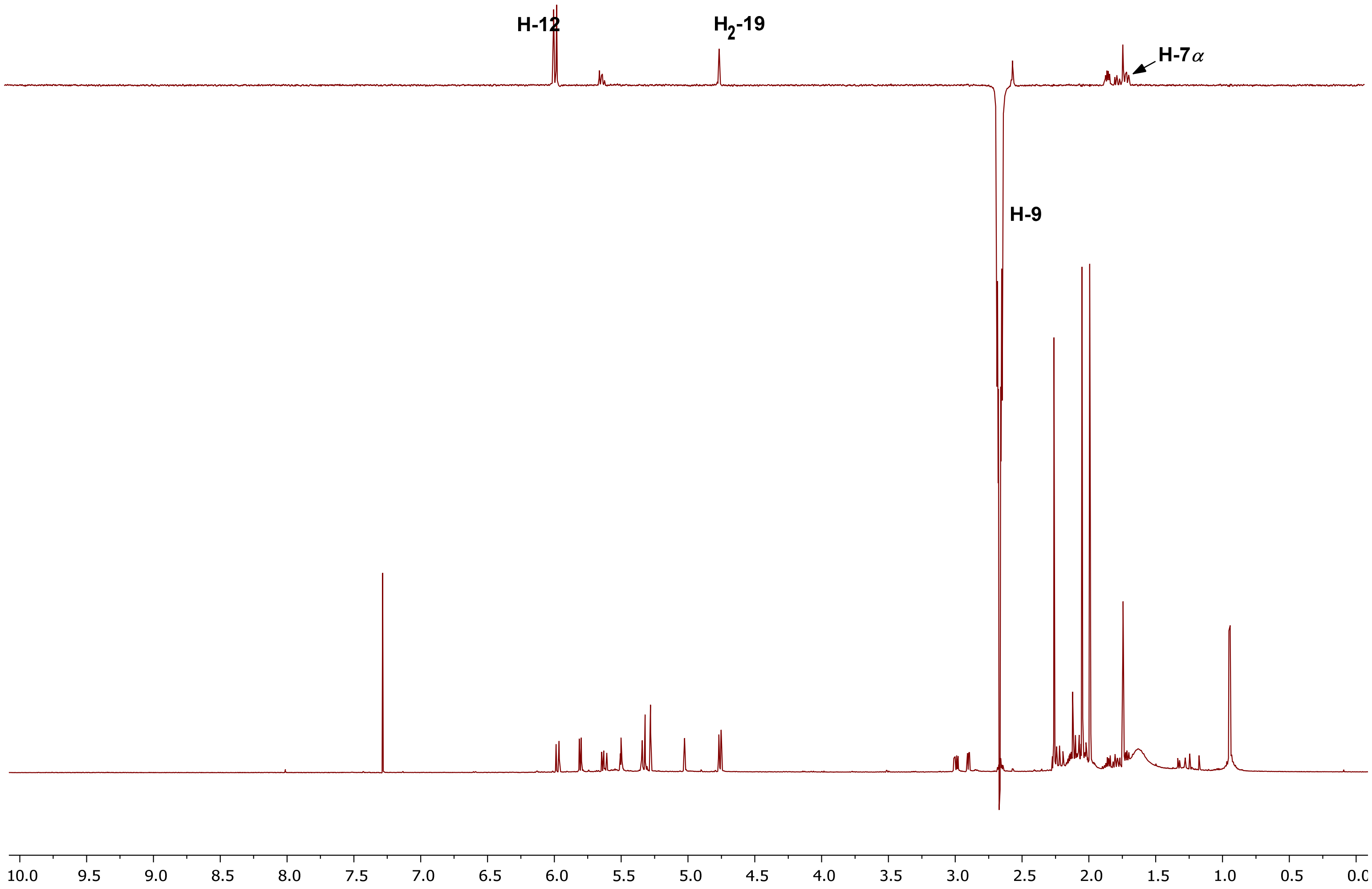

Figure S42h. 1D NOESY spectrum of compound **7**.

Elemental Composition Report

Single Mass Analysis

Tolerance = 5.0 mDa / DBE: min = -1.5, max = 50.0
Element prediction: Off
Number of isotope peaks used for i-FIT = 5

Monoisotopic Mass, Even Electron Ions
411 formula(e) evaluated with 7 results within limits (up to 10 closest results for each mass)
Elements Used:
C: 1-500 H: 0-1000 O: 0-200 Na: 0-1 K: 0-1
242_946-980_sTREP-EB12-MSe2pos 109 (2.026)
1: TOF MS ES+

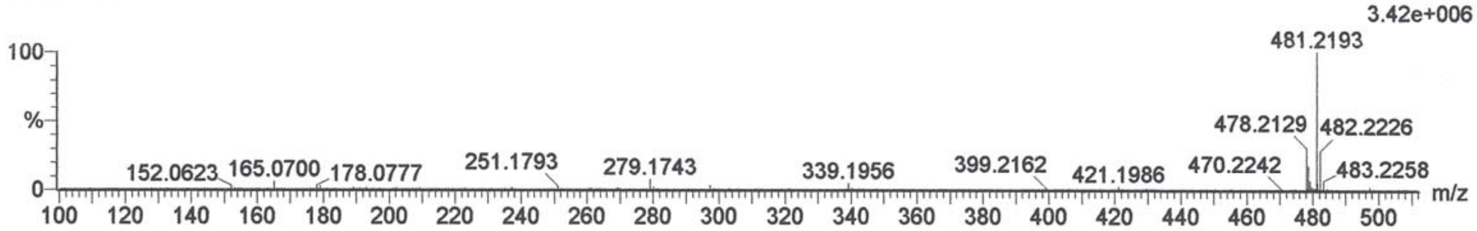

		mDa	PPM	DBE				
Minimum:				-1.5				
Maximum:		5.0	10.0	50.0				

Mass	Calc. Mass	mDa	PPM	DBE	i-FIT	Norm	Conf(%)	Formula
481.2193	481.2202	-0.9	-1.9	9.5	1638.8	0.147	86.29	C26 H34 O7 Na
	481.2204	-1.1	-2.3	4.5	1654.8	16.067	0.00	C23 H38 O8 K
	481.2180	1.3	2.7	1.5	1654.5	15.837	0.00	C21 H39 O8 Na K
	481.2168	2.5	5.2	21.5	1646.3	7.586	0.05	C35 H29 O2
	481.2226	-3.3	-6.9	12.5	1640.7	2.003	13.50	C28 H33 O7
	481.2145	4.8	10.0	13.5	1655.5	16.811	0.00	C30 H34 O3 K
	481.2143	5.0	10.4	18.5	1645.2	6.464	0.16	C33 H30 O2 Na

Figure S43. HRMS of compound **7**.

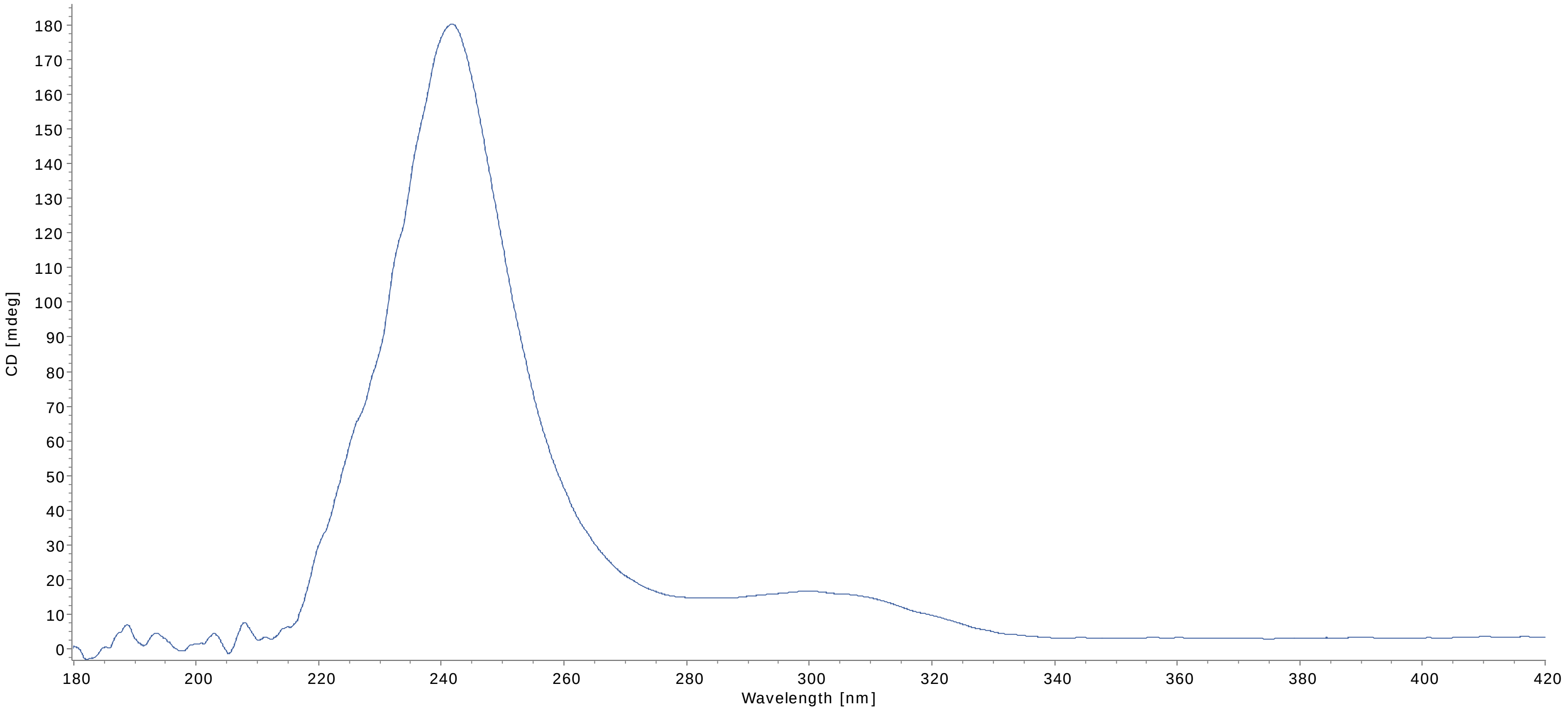

Figure S44. ECD of compound **7**.

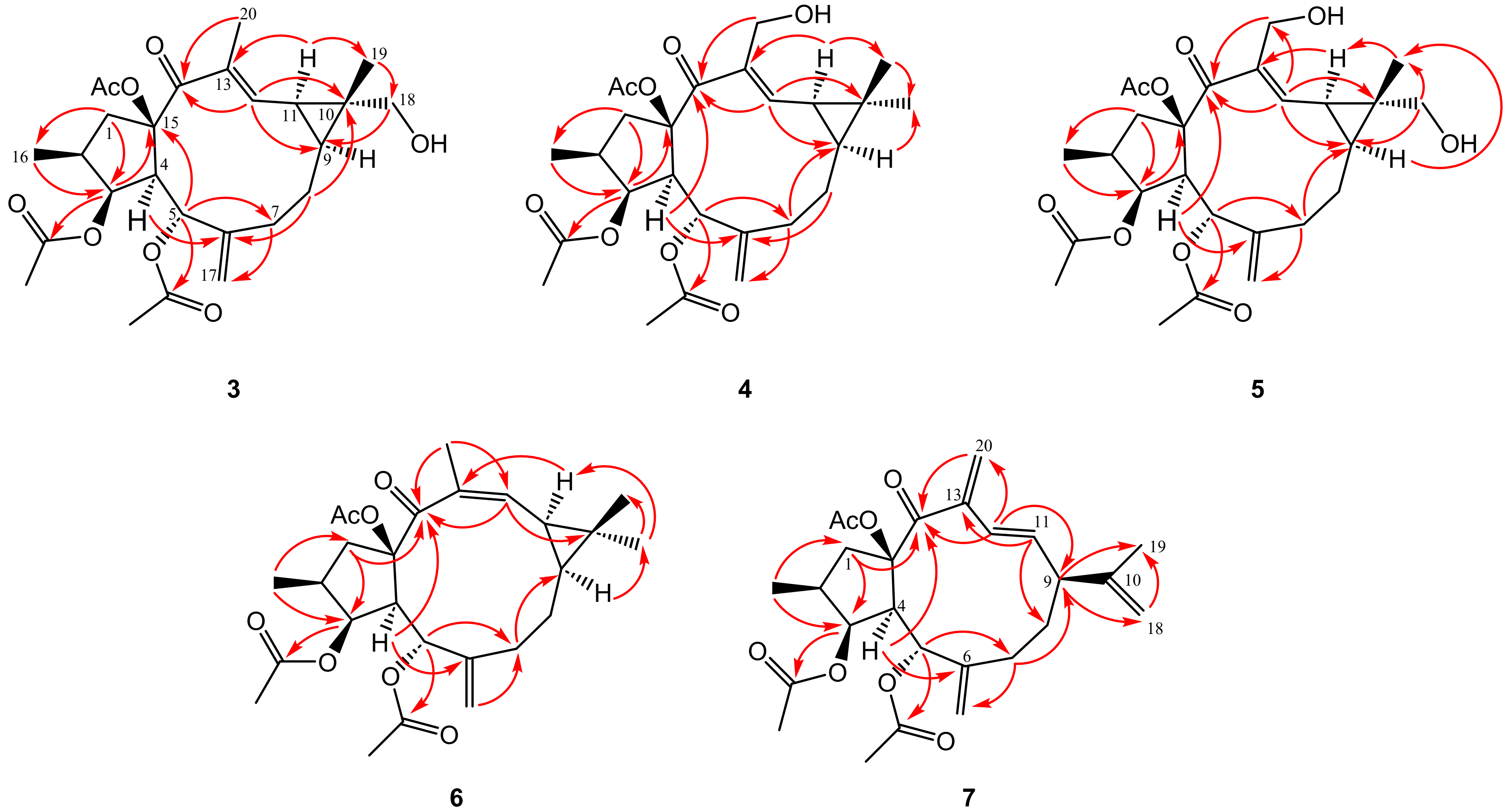

Figure S45. Selected HMBC correlations (arrow) for compounds **3-7**.
